# Annotation of suprachromosomal families reveals uncommon types of alpha satellite organization in pericentromeric regions of hg38 human genome assembly

**DOI:** 10.1016/j.gdata.2015.05.035

**Published:** 2015-06-05

**Authors:** V.A. Shepelev, L.I. Uralsky, A.A. Alexandrov, Y.B. Yurov, E.I. Rogaev, I.A. Alexandrov

**Affiliations:** a Institute of Molecular Genetics, Russian Academy of Sciences, Kurchatov sq. 2, Moscow 123182, Russia; b Department of Genomics and Human Genetics, Vavilov Institute of General Genetics, Russian Academy of Sciences, Moscow 119991, Russia; c Center for Brain Neurobiology and Neurogenetics, Institute of Cytology and Genetics, Siberian Branch of the Russian Academy of Sciences, Novosibirsk 630090, Russia; d Research Center of Mental Health, Russian Academy of Medical Sciences, Zagorodnoe sh. 2, Moscow 113152, Russia; e Department of Psychiatry, Brudnick Neuropsychiatric Research Institute, University of Massachusetts Medical School, Worcester, MA 01604, USA; f Faculty of Bioengineering and Bioinformatics, Lomonosov Moscow State University, Moscow 119234, Russia

**Keywords:** Centromeres, Alpha satellite, Suprachromosomal families, Higher-order repeats, hg38 human genome assembly

## Abstract

Centromeric alpha satellite (AS) is composed of highly identical higher-order DNA repetitive sequences, which make the standard assembly process impossible. Because of this the AS repeats were severely underrepresented in previous versions of the human genome assembly showing large centromeric gaps. The latest hg38 assembly (GCA_000001405.15) employed a novel method of approximate representation of these sequences using AS reference models to fill the gaps. Therefore, a lot more of assembled AS became available for genomic analysis. We used the PERCON program previously described by us to annotate various suprachromosomal families (SFs) of AS in the hg38 assembly and presented the results of our primary analysis as an easy-to-read track for the UCSC Genome Browser. The monomeric classes, characteristic of the five known SFs, were color-coded, which allowed quick visual assessment of AS composition in whole multi-megabase centromeres down to each individual AS monomer. Such comprehensive annotation of AS in the human genome assembly was performed for the first time. It showed the expected prevalence of the known major types of AS organization characteristic of the five established SFs. Also, some less common types of AS arrays were identified, such as pure R2 domains in SF5, apparent J/R and D/R mixes in SF1 and SF2, and several different SF4 higher-order repeats among reference models and in regular contigs. No new SFs or large unclassed AS domains were discovered. The dataset reveals the architecture of human centromeres and allows classification of AS sequence reads by alignment to the annotated hg38 assembly. The data were deposited here: http://genome.ucsc.edu/cgi-bin/hgTracks?db=hg38&hgt.customText=https://dl.dropboxusercontent.com/u/22994534/AS-tracks/human-GRC-hg38-M1SFs.bed.bz2.

**Table:** 

Specifications	
Organism/cell line/tissue	*Homo sapiens*
Sex	Both
Sequencer or array type	hg38 human genome assembly
Data format	Analyzed
Experimental factors	N/A
Experimental features	N/A
Consent	N/A
Sample source location	N/A

## 1 Direct link to deposited data

Deposited data are available here: http://genome.ucsc.edu/cgi-bin/hgTracks?db=hg38&hgt.customText=https://dl.dropboxusercontent.com/u/22994534/AS-tracks/human-GRC-hg38-M1SFs.bed.bz2.

## 2 Materials and methods

### 2.1 A general layout of AS sequences in hg38 assembly

Centromeric regions of human chromosomes in hg38 assembly [1] (GCA_000001405.15) can be divided in two main parts. One is a functional homogeneous core of each centromere which consists of thousands of copies of ~ 98% identical higher-order repeats (HORs) composed of 2–20 divergent copies of an ~ 170 bp AS monomer [2], [3]. As a rule, HORs are different on different chromosomes, but 3 non-homologous pairs of chromosomes share almost identical or very similar HORs (the so-called “paired domains” 13/21, 14/22 and 5/19) [3]. Each core is flanked by layers after layers of sequence formed by divergent monomeric or dimeric arrays devoid of homogeneous HORs [3], [4], [5], [6]. These layers are composed of slightly different types of monomers and represent the “dead” remnants of the centromeres of our pre-great ape ancestors, which had no chromosome-specific HORs, but rather monomeric or dimeric AS identical in all chromosomes with the possible exception of the Y [3], [6], [7]. The farther from the “live” homogeneous core, the older and more divergent the dead layers are [6] and more signs of “post-mortal” damage such as deletions, inversions and insertions of mobile elements they display [3], [6]. The structure of the flanking pericentromeric regions is more or less symmetrical and each specific layer is often present on both sides of homogeneous core, which performs the centromeric function and forms a kinetochore [6]. The dead divergent layers cannot function as a centromere, but form pericentromeric heterochromatin [8].

With the exception of the Y chromosome, functional HOR arrays can be classed into three “new” suprachromosomal families (SFs 1, 2 and 3), each residing on a number of chromosomes. The older non-HOR AS is divided into the two large groups SF5 and SF4. SF5 is evolutionarily younger and immediately ancestral to the new families. On most chromosomes it directly flanks the functional HOR arrays [3]. SF4 group contains all the older layers of non-HOR AS. Recently it has been subdivided into a number of SFs, most of which have not yet received formal names pending finalization of a new classification system. They are called dead AS layers and are color-coded [6]. Here we refer to the old SF4 as the SF4 + umbrella group, which includes the yellow layer (SF4 proper) and all the older layers defined in [6]. The new SF 1–3, SF5 and SF4 + groups are all composed of their own classes of monomers ([3] and Table 1) recognizable by the PERCON program [7]. In this work, we do not annotate the colored layers within SF4 + (monomeric group M1 +), as their classification has yet to be completed.

**Table 1:** Classification of monomeric types in live and dead AS layers.

Functional state, location	Old classification^a^		New classification [6]		Ancestral arrangement^b^	Age group
	SF	Monomer class, (type)	SF/colored layer	Monomer class, (type)		
Live SFs Core centromere	SF1	J1(A) J2(B)	SF1	J1(A) J2(B)	Dimeric	New
	SF2	D1(B) D2(A)	SF2	D1(B) D2(A)	Dimeric	New
	SF3	W1(B) W2(B) W3(B) W4(A) W5(A)	SF3	W1(B) W2(B) W3(B) W4(A) W5(A)	Pentameric	New
Dead SFs/layers Peripheral centromere	SF5	R1(B) R2(A)	Blue (SF5)	R1(B) R2(A)	Irregular	Old
	SF4 +	M1 + (A)	Yellow (SF4)	M1(A)	Monomeric	Old
			Yellow-striped (SF6)	V1(A)	Monomeric	Old
			Olive-green (SF?)	H1(A) H2(A)	Dimeric	Ancient
			Red (SF?)	H3(A)	Monomeric	Ancient
			Gray (SF?)	H4(A)	Monomeric	Ancient

The table summarizes the data reviewed in [3] and reported in [6].

a Used in this paper.

b In live domains ancestral arrangement can only be observed or deduced from monomer order within a HOR unit.

In previous assemblies of the human genome, most of the HOR AS was absent and the core was occupied by a centromeric gap. In the latest hg38 assembly, the gap has been filled with so-called “reference models”, which are somewhat arbitrary representations of AS HOR domains. Reference models are not real DNA sequences like traditional GenBank contigs, but instead are collections of all WGS reads, that match a certain HOR, put into a contig by the stochastic approach of using a generative Markov process, which is not expected to recreate the true long-range linear order across the entire array [1], [9]. They can however be very helpful in mapping the AS deep sequencing or WGS reads to the human genome assembly.

Due to the complex pattern of intra- and inter-chromosomal identities in the pericentromeric regions of the acrocentric chromosomes 13, 14, 21 and 22, the mapping protocol used for the new assembly was apparently unable to determine which reference model belonged to which chromosome and what were the precise locations of the AS sequences on the chromosomes. Thus, all the HOR domains, which are present on at least one of these chromosomes, were put together in a single block, and this block was placed into the former centromeric gap on each chromosome. The same block of 13 reference models arranged in about the same order appears on all four chromosomes, but individual reference models have different names on every chromosome. Note that this block includes two live centromeres (paired domains 13/21 and 14/22), of which only one is actually present on any particular chromosome. Also, the identical sets of 3 AS reference models (of which only one is alive) appear on chromosomes 5 and 19 (paired domain 5/19), and the live model from this set also appears on chromosome 1 where the HOR is very similar to 5/19 paired domain and apparently cannot be distinguished by reference model assembly process (see Tables 2 and S1).

**Table 2:** The list of unique AS reference models in hg38 assembly.

#	Chrom	Name	Size (bp)	SF	State	HOR symbol^a^[3]
1	chr1	GJ211836.1	198,076	3		
2	chr1	GJ211837.1	278,512	3		
3	chr1	GJ211855.1	63,597	3		
4	chr1	GJ211857.1	83,495	3		
5	chr1, 5, 19^b^	GJ212202.1	2,282,185	1	Live	D1Z7/D5Z2/D19Z3
6	chr2	GJ211860.1	1,902,412	2	Live	D2Z1
7	chr3	GJ211866.1	461,128	1,5		
8	chr3	GJ211867.1	13,936	1,5		
9	chr3	GJ211871.1	2,102,155	1	Live	D3Z1
10	chr4	GJ211881.1	2,031,890	2	Live	D4Z1
11	chr5	GJ211882.1^a^	83,162	5		
12	chr5	GJ211883.1	227,563	5		
13	chr5	GJ211884.1	264,463	5		
14	chr5	GJ211886.1	46,345	5		
15	chr5	GJ211887.1	142,630	1		
16	chr5, 19^b^	GJ211904.2	53,672	5		
17	chr5, 19^b^	GJ211906.2	338,504	5		
18	chr6	GJ211907.1	1,276,046	1	Live	D6Z1
19	chr7	GJ211908.1	2,658,581	1	Live	D7Z1
20	chr7	GJ212194.1	150,232	5		
21	chr8	GJ211909.1	1,843,521	2	Live	D8Z2
22	chr9	GJ211929.1	2,128,923	2	Live	D9Z4
23	chr10	GJ211930.1	249,218	1		
24	chr10	GJ211932.1	1,561,440	1	Live	D10Z1
25	chr10	GJ211933.1	48,180	1		
26	chr10	GJ211936.1	47,701	1		
27	chr11	GJ211938.1	11,969	5		
28	chr11	GJ211943.1	3,251,982	3	Live	D11Z1
29	chr11	GJ211948.1	82,575	3		
30	chr12	GJ211949.1	47,204	1		
31	chr12	GJ211954.1	2,349,957	1	Live	D12Z3
32	chr13, 14, 21, 22^b^	GJ211955.2	22,537	4 +		
33	chr13, 14, 21, 22^b^	GJ211961.2	88,022	4 +		
34	chr13, 14, 21, 22^b^	GJ211962.2	54,133	4 +		
35	chr13, 14, 21, 22^b^	GJ211963.2^c^	63,535	4 +		
36	chr13, 14, 21, 22^b^	GJ211965.2	20,670	5		
37	chr13, 14, 21, 22^b^	GJ211967.2	6670	4 +		
38	chr13, 14, 21, 22^b^	GJ211968.2	3245	4 +		
39	chr13, 14, 21, 22^b^	GJ211969.2	22,561	4 +		
40	chr13, 14, 21, 22^b^	GJ211972.2	1,134,211	2	Live	D14Z9/D22Z?
41	chr13, 14, 21, 22^b^	GJ211986.2	1198	4 +		
42	chr13, 14, 21, 22^b^	GJ211991.2	632,586	2	Live	D13Z1/D21Z1
43	chr13, 14, 21, 22^b^	GJ212205.1	340	1		
44	chr13, 14, 21, 22^b^	GJ212206.1	340	1		
45	chr15	GJ212036.1	415,278	4 +		
46	chr15	GJ212042.1	855,957	4 +		
47	chr15	GJ212045.1	1,370,146	2	Live	D15Z3
48	chr16	GJ212046.1	23,302	2		
49	chr16	GJ212051.1	1,928,003	1	Live	D16Z2
50	chr17	GJ212053.1	381,239	3		
51	chr17	GJ212054.1^c^	3,371,615	3	Live	D17Z1
52	chr17	GJ212055.1	49,431	3		
53	chr18	GJ212060.1	319,478	2		
54	chr18	GJ212062.1	4,763,584	2	Live	D18Z1
55	chr18	GJ212066.1	93,042	2		
56	chr18	GJ212067.1	39,636	2		
57	chr18	GJ212069.1	76,958	2		
58	chr18	GJ212071.1^c^	21,409	2		
59	chr20	GJ212091.1	150,723	2		
60	chr20	GJ212093.1	1,886,394	2	Live	D20Z2
61	chr20	GJ212095.1	47,956	2,5		
62	chr20	GJ212105.1	80,766	4 +		
63	chr20	GJ212107.1^c^	78,875	4 +		
64	chr20	GJ212117.1	120,944	5		
65	chrX	GJ212192.1	3,806,963	3	Live	DXZ1
66	chrY	GJ212193.1	227,095	4 +	Live	DYZ3

a Identity of reference models marked as “live” with the known live HORs of respective chromosomes was verified by BLASTing the sequences in our HOR list in [3] to the first 10,000 bp of respective reference model. In all cases multiple hits of 93% or higher were obtained.

b Only one representative member of a group of identical reference models is listed. For complete list, see Supplementary Table S1.

c Corrected versions of these reference models were obtained from K. Miga and used for analysis.

### 2.2 AS classification used by PERCON in the context of the human genome

AS was classed into five suprachromosomal families (SFs 1–3, SF4 + and SF5) according to monomeric classes in the sequence (Table 1), as described earlier [7]. Of those, SFs 1–3 are the new families of homogeneous HORs residing in functional centromeres in all autosomes and the X. In many chromosomes, on the periphery of the live HOR domain, much smaller domains formed by different new family HORs may also be present [3], [10]. These could be the remnants of formerly functional centromeres, which have been recently replaced by other new family HOR domains and have been heavily deleted since their death. Such damaged dead centromeric domains are expected to appear on both sides of a live centromere and to be somewhat less homogeneous and less regular. On the other hand, they could be just occasional amplifications of a piece of AS, the HORs which have never had centromeric function which we termed pseudocentromeres. If such pseudocentromeric HOR has amplified a piece of AS residing in a segment duplication (SD) or a piece of some atypical border sequence, it may appear as an AS domain with unexpected location or composition. Also, if a piece of a damaged old centromere is amplified, it may once again appear as homogeneous and regular as a live centromere. Minor HOR domains may belong to the same SF as the live HOR on a given chromosome (e.g. D18Z1 and D18Z2) or to a different SF (e.g. D1Z7 and D1Z5) [3], [11]. Sometimes, a peripheral small HOR domain may contain just a slight variation of the live HOR. Such variants are usually 93%–97% identical to the main HOR. At least in one case (D17Z1-B [12]), such divergent variant has been demonstrated to be active as a centromere in some individuals (centromeric epiallele) [13]. The new families have only a very small proportion of non-HOR AS presumably represented by stray pieces and domain border sequences.

SF4 + and SF5 groups are mostly formed by divergent non-HOR AS, which represents the dead centromeres of our primate ancestors [3], [6], [7]. SF5 is the youngest dead SF located distally, right next to homogeneous cores [2], [3], [9]. Usually, SF5 domains are formed by irregular alternation of R1 and R2 monomers and contain no HORs [14]. However, several exceptions were reported, such as low copy-number HOR domains on chromosomes 4, 7, 5, 19 and acrocentrics [15], [16], [21]. These low copy number HORs were perceived as occasional small scale amplifications in a recombination-prone tandem array of a dead centromere [15], i.e. pseudocentromeres that have never had centromeric function. However, it has to be tested if such HORs might occasionally play the role of transient short-lived centromeres. Here, we have found several more low copy number SF5 HORs among reference models (Table 3). Finally, SF4 + classification group represents all the more distal and older dead families. As described in [6], it contains SF4 proper, which is next and distal to SF5, pooled with all the other yet older and more distal families including SF6 and others which have not been named yet and are called dead layers and are color-coded (see Table 1). SF4 + sequences are divergent and contain no live HORs except for the relic Y chromosome-specific HOR family, which belongs to SF4 proper, reportedly one of the smaller functional HOR domains in human genome [2]. However, PERCON annotation revealed many more SF4 + HORs both among AS reference models and regular contigs (see Tables 2, S1, and S3 and Discussion section).

**Table 3:** Pure R2 regions in hg38 assembly.

SF	Location	Position in hg38	Contig	Size^a^ (bp)	R2%	B-box %	HORs on dot-matrix
SF5	6q11.1	chr6:61,326,977–61,336,104	AMYH02013791.1^b^	9127	78	2	No HOR
SF5	6q11.1	chr6:61,428,794–61,437,937	FP325349.3^b^	9143	78	2	No HOR
SF5	7p11.2	chr7:57,939,175–57,953,728	AC138789.1	10,294	94	0	No HOR
SF5	7q11.21	chr7:62,536,194–62,564,614	AC019063.4	24,011	83	2	No HOR
SF5	10p11.1	chr10:39,432,620–39,442,102	ABBA01020709.1	6981	70	0	No HOR
SF5	11p11.12	chr11:48,806,070–48,814,307	AC127495.2	8237	91	0	No HOR
SF5	12q11	chr12:37,632,794–37,639,361	AC119042.9	6567	75	0	No HOR
SF5	16p11.1	chr16:36,001,814–36,022,913	AC109490.3	21,099	94	0	No HOR, duplication 4.8 kb, identity 97.5%
SF5	16p11.1	chr16:36,079,689–36,090,000	FP325312.10	10,311	93	0	No HOR
SF5	20q11.1	chr20:29,908,640–30,038,347	ABBA01018540.1, GJ212117.1	128,442	80	0	HOR 1.4 kb
SF5	20q11.1	chr20:30,088,752–30,140,826	FP565326.9	51,870	83	0	HOR 1.4 kb^c^
SF5	Xq11.1	chrX:62,611,837–62,642,074	BX544875.1	30,237	90	3	No HOR

a Size has been corrected to exclude L1-repeats and gaps.

b These contigs are partially segment duplications of each other.

c This HOR is about 97% identical to the one in GJ212117.

The 12 monomeric classes recognized by PERCON group into 2 ancestral types, A and B (see Table 1), which may be differentiated by several variable nucleotide positions in 35–51 region of the monomer (the A/B box) [14], [17]. In the B type, this region binds the well studied CENP-B protein and in the A type it reportedly binds a certain pJalpha protein the identity of which has not been established.

### 2.3 PERCON program

The PERCON program (formerly various parts of it were called the PERCON, DIST and BREVN) was utilized for AS classification as described in [7]. It was executed in a number of steps. The first step was the identification of AS performed in two phases. Phase 1 implemented a fast database homology search with the aid of octanucleotide dictionaries. Briefly, the distance ro = f/f (random) between two fragments was calculated where f = log((N1 + N2) / 2nc); N1 and N2 were the sizes of octanucleotide dictionaries of the compared fragments; nc was the size of the intersection dictionary shared between the two fragments, and f (random) was the f value calculated for a random sequence. The ASC consensus monomer (see below) was used for generation of the N2 dictionary. The threshold value of ro = 0.6 was determined experimentally, above which no AS sequences retained in a sample. Ro = 0 for identical sequences. At phase 2, all the sequences, which cleared the threshold, were checked by an automatic dot matrix procedure. The program produced a quantitative parameter roughly reflecting the probability of a random generation of the best diagonal observed in a given dot matrix. The sequences which cleared a certain experimentally determined threshold were retained for further analysis.

The second step was AS monomer identification and SF classification. Monomers were identified by repeated alignment to the same AS consensus test sequence (ASC; shown in Fig. S1), a modified version of ALPHA-ALL consensus derived from consensus sequences of all 12 known monomeric classes [14]. Position 1 in the monomer by tradition was arbitrarily assigned to the first nucleotide of the BamHI site in chromosome X-specific HOR DXZ1 [18]. The N positions in the A/B box of ALPHA-ALL were set to the A configuration as shown in Fig. S1. The repeated alignment was performed by a modified Smith–Waterman–Gotoh algorithm [19], [20] and was stopped when relative alignment score (rs) of the monomers obtained became 0.29 or less. rs was calculated as an alignment score divided by reward for a match multiplied by the length of alignment. The alignment score was the number of matches multiplied by reward for a match minus the number of gaps multiplied by a gap opening penalty minus the number of nucleotides in gaps multiplied by a penalty for gap extension minus a number of mismatches multiplied by a penalty for mismatch. At the very ends of the monomer the alignment was not always precise and small gaps of up to 5 bp often separated the adjacent monomers. This did not affect monomer classification. Next, every monomer was classed into one of the 12 known standard monomer classes (J1, J2, D1, D2, W1–W5, R1, R2 and M1 [3]) or defined as unclassed (Um) or random (Xm) by a simple Bayesian classification procedure that utilized consensus matrices of the 12 classes of monomers together with the random matrix (shown in Fig. S1). The program estimated the probability of a hypothesis that a given monomer belonged to one of the known monomeric classes and, if it met the threshold (typically 0.9), assigned the classification. Otherwise, the monomer was deemed “unclassed” (e.g. chimeric monomers where half belongs to one class and half to another class). Altogether, 14 groups were identified by PERCON. Long sequences were processed in consecutive 5 kb windows, which overlapped by about 200 bp.

Independently, the region of the A/B box (positions 35–51) was classed in every monomer in the sequence (A box, B box, X for random, U for unclassed and Q if, in the truncated monomer, the box region was not present). Classification was performed by Bayesian classifier in the same way as for the whole monomers. The matrices for the A and B boxes were generated by summation of all the consensus matrices of individual monomeric classes that belonged to type A and type B (shown in Fig. S1). Note that the A/B classification did not assess the functionality of the B-box in CENP-B binding—it just determined to which ancestral type the monomer belonged. In some rare cases, the box classification and the monomer classification may contradict each other (e.g. R2 monomer which has a B-box). At least some studied instances of such monomers are hybrids with the box region coming from one class and the rest of the monomer from the other (data not shown).

PERCON is available for download at: https://github.com/alrsat/PERCON.

### 2.4 UCSC Browser Track

The track was created by PERCON program developed by V.A. Shepelev and I.A. Alexandrov [7]. AS monomers were identified by PERCON similarity search, extracted and distributed into the classes characteristic of the 5 SFs by a Bayesian classifier. Program output contained detailed information on AS monomers, including monomeric class, result of independent typing of the A/B box, genomic coordinates and strand orientation, which were used for the annotation track. For incomplete monomers with length less than 140 bp, the monomer class was shown in lowercase letters; for longer monomers in uppercase letters. These data generated for hg38 human genome assembly were transformed into a Browser Extensible Display (BED) format suitable for viewing as a custom annotation track in the UCSC Human Genome Browser (http://genome.ucsc.edu/). To convert PERCON output to BED format we wrote an AWK script (available at https://github.com/enigene/prcn2BED) which also color-codes the monomers according to the monomeric type. After using the prcn2BED, the resulting BED file was processed by a second AWK script (available at https://github.com/enigene/remisct) to remove duplicate segments resulting from overlap of the 5 kb windows. Of two overlapping monomers, the longer one remained intact and the shorter one was trimmed. The resulting annotation track is available at http://genome.ucsc.edu/cgi-bin/hgTracks?db=hg38&hgt.customText=https://dl.dropboxusercontent.com/u/22994534/AS-tracks/human-GRC-hg38-M1SFs.bed.bz2. The track is self-explanatory, as in the full-view mode each monomer is marked with respect to its monomeric class and the A/B type according to Table 1.

By using the Table Browser, the track data can be analyzed in text format and filtered or transformed to generate various statistics. For instance, different classes of monomers can be counted per individual chromosome or chromosomal region. Also, the overlaps with other tracks can be created and retrieved as a new track, DNA sequence, or in text format.

### 2.5 Overall statistics of AS in hg38 assembly

**Fig. 1:**
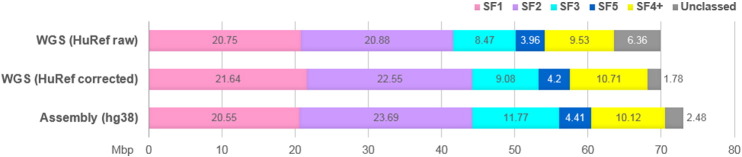
Comparison of AS SF profiles of hg38 human genome assembly and HuRef WGS dataset. The figure plots the SF content of the two datasets in Mb per haploid genome (3 × 10^9^ bp). For WGS dataset (1 million reads), the number of AS monomers identified by PERCON was multiplied by the average length of a monomer in this dataset (146 bp) and normalized to the genome size (shown as “HuRef raw”). The same amount of AS divided in proportions obtained in the same sample with bad ends trimmed and filtered for monomers 140 bp or longer (average monomer length 168 bp) is shown as “HuRef corrected” (see Fig. S2 for details). For the assembly, the length of all monomers identified by PERCON in each category was summarized directly from PERCON track using the Table Browser. In both datasets, the real amounts are slightly underestimated in a similar manner, as small gaps which PERCON often leaves between monomers due to imperfect alignment of the ends are not taken into account.

The overall statistics of AS was collected from the UCSC Browser Track using Table Browser and analyzed to control how the track data corresponded to what was known from other methods and sources. The overall detection of AS by PERCON (70.1 Mbp, 2.30%) did not differ significantly from that of RepeatMasker (http://repeatmasker.org/), as used in UCSC Browser RepeatMasker Track (70.8 Mbp, 2.32%). RepeatMasker records that had at least 98% overlap with the PERCON track constituted 69.5 Mbp or 98.2% of total RepeatMasker detection. RepeatMasker records that had no overlap with PERCON AS SF track constituted 3628 bp or 0.01% of total detection. The latter records were all small fragments shorter than one monomer. The size of DNA occupied by monomers of each SF determined in hg38 assembly is shown in Fig. 1 where it is compared to the data obtained in the analysis of WGS database. One million HuRef WGS reads (PRJNA19621) obtained by random DNA fragmentation were processed by PERCON in the same way as described previously for analysis of the BAC ends [7]. There was no dramatic difference in SF profiles between the sets, except the proportion of unclassed monomers in WGS (6%) was predictably higher than in the assembly (2.5%). Both a large number of truncated monomers at the ends of WGS reads and the low quality of sequence at the ends of Sanger reads contributed to this difference (Fig. S2). To correct for these factors we evaluated the effect of trimming the bad ends using the LUCY program with default parameters [22] and of filtering the dataset for monomers 140 bp or longer (such monomers are marked with upper case letters in PERCON output). The results are shown in Fig. S2, where it can be seen that each step reduced both the number of unclassed monomers and the total AS detection. To combine good detection with more accurate SF measurements we used the SF proportions obtained in the double-filtered sample to divide the AS amount obtained in the unfiltered sample. After such correction (see legend to Fig. S2 for more details) the proportion of unclassed monomers dropped to 2% and the WGS data appeared to be in fairly good concordance with the assembly. The only significant difference was a larger SF3 in the assembly. The reasons for this discrepancy we did not investigate. A more detailed discussion of the possible sources of unclassed monomers was provided in [7]. The proportions of SFs in human genome shown in Fig. 1 are, as much as we know, the first accurate estimate obtained in a non-biased sample. The results differ significantly from the ones in [7] which was our previous attempt to SF-class a large sample of AS fragments. The difference is presumably due to restriction enzyme bias in the older sample.

The above statistics suggest that monomeric maps for most human AS sequences can be obtained without running PERCON, but simply by finding the same exact sequence or a very similar copy of it in the hg38 assembly.

### 2.6 Annotation of AS HOR reference models

For quick reference, we also provide tables of SF-annotated AS reference models as they appear in the assemblies of individual human chromosomes. Although annotation of individual HORs is beyond the scope of this work, we indicate the known live HORs, which are the largest reference models (except for the Y chromosome). Altogether 109 reference models are used in hg38 assembly (Table S1). After correction for identical models in different chromosomes, 66 unique models remain (Table 2). Of these, 18 unique models represent 22 live centromeres of autosomes, as chromosomes 13/21, 14/22 and 1/5/19 share the same live reference models. Two additional models represent live centromeres of sex chromosomes. All of them are classed in SFs as previously reported for respective live centromeres and can be recognized by high identity to the known centromeric sequences from the list shown in [3] (see Tables 2 and S1). Of the remaining 46 models, 24 contain variant live HORs, dead HORs or pseudocentromeres of the new families (SF1-3), and 10 contain SF5 HORs which were not known to form live centromeres and were traditionally perceived as pseudocentromeres. Previously, such HORs were reported on chromosomes 4, 5, 7, 19 and acrocentrics [15], [16], [21]. However, recent evidence shows that live centromeres of orangutan are likely formed by SF5 HORs [23], so some SF5 HOR domains may in fact be dead centromeres. Finally, 12 other unique pericentromeric reference models were classed as SF4 +. Except for the live SF4 + centromere of chromosome Y, SF4 + HORs were not widely reported in man. The only example known to us was the pTRA-2 AS clone [21] which was classed as SF4 [3] and shown to form a cluster of 75 HORs in the short arm of chromosome 21 [24] (see the Discussion section below). In particular, SF4 + HORs are present in chromosomes 15, 20 and the 13/14/21/22 group.

Due to some problem in the reference model assembly process, 5 unique reference models (marked in Tables 2 and S1) were assembled incorrectly, with a reverse order of monomers in a HOR (K. Miga, personal communication). The corrected versions of these reference models were obtained from K. Miga and used throughout our analysis.

Note that non-HOR AS is not supposed to appear in reference models.

Evaluation of HORs in the sequences listed in Table 3, Table 4 was performed using the dot-matrix construction program from http://www.vivo.colostate.edu/molkit/dnadot/, Gepard program [25] or the REVN program written by V.A. Shepelev [26].

**Table 4:** SF1/SF5 and SF2/SF5 mixed AS regions in hg38 assembly.

SF	Location	Position in hg38	Contig	Size^a^ (bp)	SF1%	SF2%	SF5%	HORs on dot-matrix
SF2/SF5	2q21.2	chr2:132,237,392–132,247,263	AC097532.3	9871	0	22	58	No HOR
SF1/SF5	3p11.1	chr3:90,482,385–90,722,299	ABBA01004652.1, AEKP01209350.1, ABBA01004653.1, AEKP01209353.1, ABBA01004654.1, ABBA01004655.1, ABBA01004656.1	229,441	40	0	41	No HOR
SF1/SF5	3p11.1 3q11.1	chr3:90,772,554–91,233,510	GJ211866.1	460,956	48	0	35	HOR 1.7 kb
SF1/SF5	3q11.1	chr3:91,233,782–91,247,547	GJ211867.1	13,765	57	0	31	No HOR other than AB dimer, identity ~ 93%
SF1/SF5	3q11.1	chr3:91,247,775–91,286,183	ABBA01000927.1, ABBA01000928.1, ABBA01000929.1, ABBA01000930.1, ABBA01000931.1	17,430	50	0	32	HOR 1.7 kb^b^
SF1/SF5	3q11.1	chr3:93,716,246–93,725,946	ABBA01026974.1	9700	35	0	49	No HOR
SF1/SF5	6q11.1	chr6:60,230,028–60,241,613	AC244258.2	11,401	28	0	53	No HOR
SF1/SF5	6q11.1	chr6:61,371,445–61,427,364	AEKP01189806.1, AEKP01189805.1, AEKP01189804.1, AEKP01189803.1, AEKP01189802.1, FP325349.3	55,519	30	0	57	No HOR
SF2/SF5	7q11.1	chr7:61,096,433–61,103,082	AC142121.2, AC017075.8	6649	0	38	46	No HOR
SF1/SF5	8p11.1	chr8:43,940,231–43,965,733	AC127507.4, AC144576.3	22,886	15	0	74	No HOR
SF1/SF5	8q11.1	chr8:45,946,092–45,971,262	AC118650.5	22,549	16	0	70	No HOR
SF2/SF5	9p11.2	chr9:40,556,928–40,565,104	AMYH02020868.1, FP325318.4	7524	0	40	46	No HOR
SF2/SF5	9p11.2	chr9:40,862,745–40,873,147	AL353626.5	10,402	0	28	50	No HOR
SF1/SF5	10p11.1	chr10:39,548,571–39,555,979	ABBA01020707.1	7408	35	0	52	No HOR
SF1/SF5	12p11.1	chr12:34,686,342–34,715,037	AC144535.4, AUXG01000432.1	28,658	60	0	30	No HOR
SF2/SF5	16p11.2	chr16:34,219,066–34,252,724	AC136932.4, ABBA01017803.1	30,263	0	35	36	No HOR
SF2/SF5	20q11.1	chr20:28,509,094–28,556,877	GJ212095.1	47,783	0	27	54	HOR 1.9 kb
SF2/SF5	22q11.1	chr22:15,965,313–15,972,300	AC145543.3	6987	0	41	34	No HOR

a Size has been corrected to exclude L1-repeats and gaps.

b Same HOR as in GJ211866.

## 3 Discussion

Inspection of the AS assembly track shows that PERCON adequately and comprehensively classes AS monomers and that they are organized predominantly into the major known modes characteristic of the known SFs. No long arrays of unclassed monomers are observed. SF1 and SF2 sequences are uniformly arranged in arrays with J1J2 and D1D2 dimeric periodicities, the remnants of W1W2W3W4W5 pentameric order can be discerned in SF3 sequences, and SF5 clusters demonstrate irregular alternation of R1 and R2 monomers. Although HORs are not annotated in the track, these repeats can be easily seen in SF3 and SF5 due to reiteration of complex patterns of W1-5 or R1 and R2 monomeric classes. In more dull dimeric sequences, HORs can often be seen due to some irregularity which occurs once or twice in a HOR, like the occasional Um, R1 or R2 monomer often found in SF2 HORs, or some other breach of dimeric periodicity like D1D1 dimer in D18Z2. In most cases, these structural peculiarities faithfully reflect the features of individual HORs reported in literature, but often the reiteration of HORs in reference models appears to be imperfect or even dramatically disturbed. Whether this reflects the true genomic arrangement or some problem in the algorithm of reference model formation remains to be investigated.

We noted a few previously unreported or poorly-reported minor modes of AS organization, as follows: (i) relatively long clusters composed entirely or almost entirely of R2 with very few or no R1; (ii) mixed occurrence of J1 and J2 monomers alternating with R1 and R2 over relatively long regions, or the same kind of pattern with D1D2/R1R2 alternation; (iii) SF4 + HORs, which appear to be no less common than long known SF5 HORs. Below we will briefly comment on these unusual modes.

Pure R2 clusters were predicted by our scenario of SF5 formation and of the origin of the new families [3], [6], [7], [14], but were actually found only recently due to their relative rarity [23]. We proposed that, in the common ancestor of orangutan and man, the centromeres were formed by pure R2 arrays and this was the last generation of type A centromeres in the human lineage. These centromeres, like all previous AS centromeres, had no CENP-B binding sites. At some point, R1 (type B) monomer, which had a CENP-B binding site, but was otherwise very similar to R2, formed due to several point mutations. The presence of CENP-B endowed this monomer with an ability to spread by irregular transposition-like process only within live R2 centromeres resulting in fixation of the B-box which ruined the regularity. These irregular AB centromeres were less efficient than regular ones and were rescued in African apes by amplification of AB type HORs of the new families, which restored regularity. In the orangutan branch, the regularity was restored by amplification of diverse SF5 HORs (different in different chromosomes), some of which were pure R2, some had both R1 and R2 in an irregular pattern, and some had R1R2 inner dimers [23]. The non-HOR pure R2 clusters listed in Table 3 are likely the pieces of pure R2 arrays which removed from the bulk centromeres due to some chromosomal rearrangements (and therefore died) before the R1 invasion and were thus immune to R1 insertion. The same logic suggests that the gradient of R1 density in human SF5 arrays documented in Table S2 reflects the gradual displacement and death of irregular SF5 domains and can be perceived as a clock timing the R1 invasion. Presumably, the few R1-rich SF5 regions in the human genome (Table S2) are the ones which were functional (and thus exposed to R1 invasion) for the longest time. The large pure R2 HOR domain in chromosome 20 (GJ212117 and the adjacent regular contig FP565326) would hint that regular SF5 centromeres may have played some role in the early evolution of African apes before they were replaced by the new families. For such old, dead HORs, one would expect somewhat decreased homogeneity and the presence in chimpanzee and gorilla genomes. We found inter-HOR identity of 98.5% and no high identity hits in great ape WGS datasets (data not shown). Thus, the HOR is unlikely to be a relic of an era of SF5 centromeres, but is probably a more recent pseudocentromere which appeared via amplification of a piece of a dead pure R2 array.

Mixed SF1/SF5 and SF2/SF5 domains documented in Table 4 occupy relatively large space in the assembly because they are amplified in chromosomes 3 and 20, while non-HOR mixed regions are a tiny fraction. Potentially, three explanations of such mixes are feasible. They could be: (i) former pure SF1 or SF2 domains that were heavily deleted with formation of a large number of hybrid monomers (e.g. half D1, half D2) which are usually classed as Um or either R1 or R2, depending on configuration of the A/B box in the monomer (data not shown). Solitary (one per HOR) apparent SF5, but actually hybrid D1/D2 monomers are often present in SF2 HORs (data not shown); (ii) mixes of genuine new SF and SF5 monomers, which perhaps formed on the border of SF5 and live centromeres by recombination across the border, and; (iii) clusters formed by early versions of D and J monomers, which differed from their R type progenitors much less than the later “mature” versions that formed most of the HORs in respective SFs. (i) and (iii) should be regarded as “apparent” or “false” mixes and classed as respective new SF. (ii) is a “genuine” mix. All three types can potentially be HOR-amplified, which would greatly enlarge their genomic proportion. The (iii) seems to be true at least in mixed HOR arrays in chromosomes 3 and 20, as most of R1 and R2 monomers in respective HORs possess some of the mutations characteristic of J1 and J2 or D1 and D2, respectively (see detailed analysis in Supplementary note 1). This makes the arrays only “apparent mixes” and allows classing them as SF1 and SF2, respectively. High homogeneity and absence in African apes indicate that both mixed HOR arrays are likely pseudocentromeres. However, as both have divergent ancestral internal periodicities (see Supplementary note 1), these pseudocentromeres may have been formed by recent amplification of more ancient, dead HOR centromere material. Options (i) and (ii) may apply to other mixed domains which have no clear HORs. They warrant careful detailed investigation.

So far, only two different non-orthologous live Y chromosome SF4 + HORs were known (DYZ3 in man and BACs AC195625, AC156580, AC163730, AC163738 in chimpanzee) [27], [28]. Also, evidence for live SF4 + HORs in gibbons has been reported [6], [29], [30]. Additionally, the D21Z5 SF4 + HOR, also known as pTRA-2, (X55367, corresponds to GJ212124) was convincingly demonstrated on the sequence level [21], [24]. The latter appears to be a segmental duplication also present on other acrocentric chromosomes. In the human assembly, in addition to DYZ3, we class 12 reference models as SF4 +. Eight of them are part of the group assigned to chromosomes 13/14/21/22, but not all of them necessarily reside on all of these chromosomes. For instance, another member of this group, the SF2 D13Z1/D21Z1 live HOR forms the live centromeres of chromosomes 13 and 21, but is not massively present in chromosomes 14 and 22 [3]. The same applies to chromosomes 14/22 live HOR which is absent in chromosomes 13 and 21. Also, not all of them are confirmed by regular contigs (see Table S2). Another four SF4 + models reside on chromosomes 15 and 20 (2 on each). The GJ212105 HOR is also located in regular contigs ABBA01015870 and AL837517 in the same area on 20q. The latter contig has 54 kb HOR domain with HOR length 1872 bp. The high HOR similarity of 99.9%-100% is very unusual, which implies recent amplification. The large size of chromosome 15 reference models (GJ212036 and GJ212042) raises an interesting possibility that they are dead SF4 + HOR centromeres. Such centromeres were reported only in gibbons [30], but not in great apes, with the notable exceptions of human and chimpanzee Y chromosomes. In these cases, however, the SF4 + centromeres function in chromosomes that do not have more recent generations of AS, such as SF5 or the new families, which presumably would compete for centromeric function more efficiently than SF4 + [6]. Arguably, in chromosome 15, which has SF2 functional centromere and some SF5 as well, the SF4 + HOR domains may only be the dead centromeres that date back to the time before the great apes, when SF5 and the new families did not exist. However, high homogeneity (99%) of these HORs, both in the reference models and in regular contigs (listed in Table S3) and their absence in genomes of chimpanzee and gorilla (data not shown) argue that they are recent mega-scale pseudocentromeres.

Our theoretical scenario of AS centromere evolution [6] states that only a functional centromere can stably maintain the mega-amplified state and homogeneity characteristic of live centromeres due to the involvement of hypothetical kinetochore-associated recombination machine (KARM). There are at least two possibilities to reconcile the existence of mega-scale pseudocentromeres in the short arm of chromosome 15 with this view. First, it could be just a rare occasion of a very large and very recently amplified array, which is now experiencing shrinking and degradation that will become evident with time. The second, and more intriguing, possibility is that the short arms of acrocentric chromosomes possess their own special recombination machine involved in amplification and homogenization of the arrays of ribosomal RNA genes located there [31]. The machinery could also be used by some DNA repeats in the surrounding heterochromatin, which would contribute to generation and maintenance of a heterochromatic milieu of the short arms of acrocentric chromosomes apparently required for proper functioning of the nucleolus organizer regions.

The following are the supplementary data related to this article.

Supplementary Figures S1 and S2, Supplementary Tables S2 and S3, and Supplementary Note 1.

Supplementary Table S1

## Conflict of interest

The authors declare that there are no conflicting interests.
