# Supplementary material for: Annotation of suprachromosomal families reveals uncommon types of alpha satellite organization in pericentromeric regions of hg38 human genome assembly
Source: Genom Data. 2015 Jun 5;5:139–46. doi: 10.1016/j.gdata.2015.05.035 (PMC4496801; doi:10.1016/j.gdata.2015.05.035)
Supplement: Supplementary file 1 — Supplementary Figures S1 and S2, Supplementary Tables S2 and S3, and Supplementary Note 1. [file mmc1.docx]

**Supplementary data**

**Table S1. SF annotation of complete set of AS reference models in hg38 human genome assembly.**

The blocks of 13 identical models that appear in chromosomes 13, 14, 21 and 22; 3 identical models in chromosomes 5 and 19; and one of the latter that also appears in chromosome 1 are marked blue. Reverse-assembled models, which were replaced by corrected versions, are marked. If you have the PERCON Browser track open, SF annotation of a reference model opens in a new window by clicking on its name.

**Table S2. R1 density gradient in SF5 arrays of hg38 assembly.**

This table compares the pure R2 arrays shown in Table 3 to a representative list of human SF5 domains. It can be seen that the density of R1 (B) monomers is very different in individual arrays and ranges from ~0% in pure R2 domains to ~60% in the most R1-rich domains with all the intermediate stages present. The table was made by manual inspection of a special track showing only R1 and R2 monomers. It shows all SF5 reference models and most of the more prominent non-HOR SF5 regions in hg38 assembly.

**Table S3.** ­­­**Presence of SF4+ HORs in AS reference models and genomic contigs.**

It is not known to what extent AS reference models are representative of the true genomic arrangement of respective sequences. Among others, we considered a possibility that some shorter reference models could be created artificially and represented not a tandem HOR region or regions, but a system of SDs one or several of which had an inner tandem duplication. Such tandem duplication would qualify a sequence as a HOR and could seed a reference model which brings together the dispersed copies of the SD and arranges their parts into an array. Therefore we wanted to make sure that SF4+ reference models really existed and sought to confirm them by genomic sequences. It appeared that a number of longer models could be confirmed by regular contigs (marked azure) and some, mostly shorter, could not (marked yellow), which left their nature open to investigation. Additionally, some models could be confirmed only by PacBio contigs (accession numbers starting with JSAF02, marked lilac) which also could possibly misrepresent genomic arrangement of tandem repeats, as their sequences were generated by SMRT technology using PacBio's Quiver algorithm to improve the base accuracy [1]. It is not known if this procedure could artificially decrease the divergence of neighboring HOR copies or even create false HORs, so the PacBio contigs were considered to be only a tentative confirmation. Note that artificial contraction of a non-AS HOR domain was demonstrated in clone CH17-41E14 as a result of using SMRT technology [2]. It is possible that an opposite process of artificial expansion could take place as well. Below, we present further details of Table S2 construction. Genomic contigs which contained regions highly identical to the sequences in AS reference models were identified by BLASTing HORs from a reference model to human contigs and HTGS databases. A copy of a HOR to use as a probe was picked up by finding a region in a reference model with regular diagonals and cutting out an appropriate sequence fragment. High-identity (95%-100%) hits were then searched for regular tandem HORs by dot-matrix using REVN program written by V.A. Shepelev [3]. If the sequences with high identity HORs were obtained, only such sequences were listed. If no contigs with regular HORs were found, the sequences with solitary or partial HOR copies were listed and an appropriate note was made. PacBio JSAF02 contigs were listed only if they contained tandem HORs and only if no regular contigs with tandem HORs were found. In some arrays, two or more kinds of variant HORs of different lengths were found intermingled and, therefore, only ragged diagonals were observed on the dot matrix. This was marked as “HOR-like structure”. Similar dot-matrix patterns are often found on the flanks of more regular HOR domains and they contain less perfect copies of the same HOR. As a rule, if good HOR arrays were present nearby, the imperfect HOR domains were not mentioned.

**Figure S1. Consensus sequences and matrices used in this work and/or utilized by PERCON program.**

1. The A/B box consensus matrices used in PERCON:
   - PERCON consensus matrix for AS box A (positions 35-51)
   - PERCON consensus matrix for AS box B (positions 35-51)
   - PERCON random matrix for AS box X (positions 35-51)
2. Consensus matrices for 13 monomeric classes used in PERCON. There is no exact correspondence between these consensus matrices and the consensus sequences shown in section C of this figure, as they were formed at different time points from the sets of monomers which were available at the time. The differences are slight and mainly involve “N” and “-“ in positions where different letters have about 50% frequency and may or may not reach the 50% threshold set for consensus derivation depending on exact composition of the set of monomers.
   - PERCON consensus matrix for AS J1 monomer
   - PERCON consensus matrix for AS J2 monomer
   - PERCON consensus matrix for AS D1 monomer
   - PERCON consensus matrix for AS D2 monomer
   - PERCON consensus matrix for AS W1 monomer
   - PERCON consensus matrix for AS W2 monomer
   - PERCON consensus matrix for AS W3 monomer
   - PERCON consensus matrix for AS W4 monomer
   - PERCON consensus matrix for AS W5 monomer
   - PERCON consensus matrix for AS R1 monomer
   - PERCON consensus matrix for AS R2 monomer
   - PERCON consensus matrix for AS M1 monomer
   - PERCON consensus matrix of Xm random monomer
3. Aligned consensus sequences of alpha satellite monomer classes used in this work. In cases where the sequences published in [4] and deposited in GenBank were modified, the position in GenBank sequence is shown first and the position in an updated sequence is shown after an arrow.
   - consensus A type AJ131207 – modified
   - consensus B type AJ131208
   - consensus J1 AJ130753
   - consensus J2 AJ130754 – modified
   - consensus D1 AJ130751
   - consensus D2 AJ130752
   - consensus W1 AJ130758
   - consensus W2 AJ130759
   - consensus W3 AJ130760
   - consensus W4 AJ130761
   - consensus W5 AJ130762
   - consensus R1 AJ130756
   - consensus R2 AJ130757
   - consensus M1 AJ130755

**Figure S2. Comparison of AS SF profiles of hg 38 human genome assembly and HuRef WGS dataset.**

This figure is an extended version of Figure 1. In addition to the latter, it shows both the effects of trimming of the bad ends by Lucy (“HuRef trimmed”; average monomer length 146 bp) and the results of filtering of the trimmed sample for monomers of 140 bp or longer (“HuRef trimmed & long monomers”; average monomer length 168 bp). It can be seen that each step reduced both the number of unclassed monomers and the total AS detection. To combine good detection with more accurate SF measurements, we used the SF proportions obtained in the double-filtered sample to divide the AS amount obtained in the unfiltered sample (“HuRef corrected”; average monomer length 146 bp). For comparison, the average monomer length in hg38 assembly was 164 bp. This value was not used in calculations, as the length of AS monomers in the assembly was summarized directly using the Table Browser.

**Supplementary note 1. Phylogenetic analysis of mixed SF HORs.**

**Figure S1**

A. The A/B box consensus matrices used in PERCON

PERCON consensus matrix of alpha satellite box A (positions 35-51)

# A C G T -

35 0 860 30 70 40

36 400 20 0 560 20

37 740 10 140 90 20

38 100 170 20 690 20

39 40 80 800 60 20

40 20 10 810 140 20

41 40 60 0 870 30

42 490 20 400 50 40

43 120 40 810 10 20

44 790 10 60 110 30

45 920 20 10 20 30

46 890 10 20 30 50

47 820 10 60 80 30

48 10 10 960 10 10

49 20 40 900 20 20

50 870 20 70 20 20

51 940 0 10 20 30

PERCON consensus matrix of alpha satellite box B (positions 35-51)

# A C G T -

35 40 540 10 400 10

36 0 10 10 970 10

37 10 10 0 970 10

38 10 980 0 10 0

39 50 40 820 80 10

40 20 20 10 950 0

41 10 10 20 950 10

42 10 60 920 10 0

43 0 20 960 20 0

44 1000 0 0 0 0

45 960 10 10 20 0

46 900 0 60 20 20

47 10 890 30 70 0

48 30 30 920 0 20

49 10 20 950 20 0

50 20 20 930 20 10

51 830 0 10 150 10

PERCON consensus matrix of alpha satellite box X (positions 35-51)

# A C G T -

35 25 25 25 25 0

36 25 25 25 25 0

37 25 25 25 25 0

38 25 25 25 25 0

39 25 25 25 25 0

40 25 25 25 25 0

41 25 25 25 25 0

42 25 25 25 25 0

43 25 25 25 25 0

44 25 25 25 25 0

45 25 25 25 25 0

46 25 25 25 25 0

47 25 25 25 25 0

48 25 25 25 25 0

49 25 25 25 25 0

50 25 25 25 25 0

51 25 25 25 25 0

B. Consensus matrices for 13 monomeric classes used in PERCON. There is no exact correspondence between these consensus matrices and the consensus sequences shown in section C of this figure, as they were formed at different time points from the sets of monomers which were available at the time. The differences are slight and mainly involve “N” and “-“ in positions where different letters have about 50% frequency and may or may not reach the 50% threshold set for consensus derivation depending on exact composition of the set of monomers.

PERCON consensus matrix of alpha satellite J1 monomer

# A C G T -

1 78 3 5 14 0

2 89 0 8 3 0

3 0 0 3 97 0

4 0 3 0 97 0

5 0 3 3 92 2

6 3 24 65 8 0

7 0 97 3 0 0

8 97 0 0 3 0

9 78 0 16 5 1

10 0 3 92 3 2

11 0 8 11 78 3

12 0 0 97 0 3

13 3 5 81 8 3

14 97 0 0 0 3

15 3 3 65 27 2

16 95 0 3 0 2

17 8 8 0 81 3

18 3 0 0 95 2

19 3 0 3 86 8

20 22 68 3 5 2

21 57 3 24 11 5

22 86 0 11 0 3

23 5 8 78 5 4

24 3 92 0 5 0

25 11 5 84 0 0

26 19 78 3 0 0

27 11 5 0 84 0

28 3 0 0 97 0

29 0 11 3 86 0

30 3 5 86 5 1

31 100 0 0 0 0

32 27 8 57 8 0

33 5 0 89 0 6

34 8 6 0 14 72

35 3 81 8 8 0

36 62 3 3 32 0

37 86 3 5 5 1

38 11 19 3 65 2

39 8 11 70 11 0

40 11 5 70 14 0

41 0 16 0 84 0

42 84 3 5 8 0

43 0 3 97 0 0

44 95 0 2 3 0

45 92 8 0 0 0

46 97 0 0 0 3

47 81 0 3 14 2

48 3 3 94 0 0

49 0 0 100 0 0

50 95 0 5 0 0

51 95 0 0 5 0

52 100 0 0 0 0

53 0 16 0 84 0

54 97 0 3 0 0

55 3 5 0 92 0

56 0 100 0 0 0

57 0 0 0 100 0

58 0 3 0 97 0

59 2 95 0 3 0

60 22 22 53 3 0

61 8 16 0 76 0

62 78 3 14 5 0

63 3 0 5 92 0

64 81 3 5 11 0

65 97 3 0 0 0

66 86 5 0 3 6

67 97 0 0 0 3

68 73 16 8 3 0

69 5 76 14 5 0

70 5 8 3 84 0

71 100 0 0 0 0

72 0 16 84 0 0

73 100 0 0 0 0

74 3 92 0 5 0

75 89 0 11 0 0

76 8 0 92 0 0

77 92 3 0 5 0

78 86 3 8 0 3

79 0 3 16 81 0

80 3 86 8 3 0

81 92 0 8 0 0

82 16 0 0 84 0

83 0 0 0 100 0

84 0 97 3 0 0

85 5 5 8 81 1

86 0 97 0 3 0

87 100 0 0 0 0

88 3 3 94 0 0

89 95 3 2 0 0

90 97 3 0 0 0

91 89 0 3 3 5

92 8 86 5 0 1

93 3 11 2 84 0

94 51 8 32 8 1

95 3 92 5 0 0

96 0 0 8 92 0

97 0 5 11 81 3

98 5 0 3 92 0

99 0 0 97 3 0

100 0 8 5 86 1

101 3 3 89 5 0

102 100 0 0 0 0

103 0 5 0 95 0

104 3 5 92 0 0

105 0 0 8 92 0

106 0 16 78 5 1

107 0 0 0 100 0

108 3 0 94 3 0

109 0 78 0 19 3

110 27 8 62 3 0

111 0 0 0 100 0

112 0 0 0 100 0

113 3 89 5 3 0

114 92 8 0 0 0

115 89 0 11 0 0

116 0 95 0 5 0

117 8 0 0 92 0

118 0 97 3 0 0

119 94 3 0 3 0

120 5 95 0 0 0

121 86 5 5 3 1

122 5 5 86 3 1

123 78 0 11 11 0

124 0 3 97 0 0

125 0 0 0 100 0

126 8 0 10 82 0

127 5 5 18 71 1

128 92 8 0 0 0

129 92 3 0 5 0

130 3 92 2 3 0

131 21 63 5 11 0

132 2 3 0 95 0

133 0 3 0 97 0

134 0 5 0 95 0

135 0 95 0 5 0

136 0 5 0 95 0

137 0 18 0 82 0

138 0 5 0 95 0

139 8 3 3 82 4

140 5 55 32 8 0

141 89 0 3 8 0

142 0 11 0 89 0

143 84 0 8 0 8

144 3 8 89 0 0

145 97 3 0 0 0

146 3 0 94 3 0

147 3 79 10 8 0

148 92 0 3 5 0

149 0 3 89 8 0

150 0 0 0 100 0

151 0 3 2 95 0

152 11 3 2 84 0

153 5 3 68 24 0

154 3 0 97 0 0

155 97 0 0 3 0

156 97 0 3 0 0

157 100 0 0 0 0

158 0 87 8 5 0

159 92 5 0 0 3

160 0 92 8 0 0

161 8 3 0 89 0

162 0 92 3 5 0

163 11 0 0 89 0

164 5 16 53 26 0

165 0 5 3 92 0

166 0 21 0 79 0

167 0 0 0 100 0

168 0 0 97 3 0

169 3 5 0 92 0

170 87 0 8 5 0

171 62 3 32 0 3

PERCON consensus matrix of alpha satellite J2 monomer

# A C G T -

1 86 0 3 11 0

2 33 0 61 6 0

3 3 6 2 89 0

4 0 89 8 3 0

5 6 0 0 94 0

6 0 0 100 0 0

7 0 97 3 0 0

8 94 3 0 3 0

9 83 6 8 3 0

10 3 8 83 6 0

11 3 6 5 86 0

12 6 0 89 3 2

13 3 6 83 3 5

14 89 0 3 8 0

15 0 0 0 100 0

16 97 0 0 3 0

17 14 3 2 81 0

18 6 6 2 86 0

19 0 6 0 94 0

20 6 0 86 8 0

21 3 0 97 0 0

22 91 6 0 3 0

23 6 89 2 3 0

24 0 86 6 8 0

25 6 6 7 81 0

26 6 92 2 0 0

27 5 14 0 81 0

28 0 0 0 100 0

29 11 0 0 89 0

30 0 3 94 3 0

31 92 0 5 3 0

32 3 0 94 3 0

33 0 0 100 0 0

34 2 92 0 6 0

35 2 92 0 6 0

36 0 0 0 100 0

37 0 3 0 97 0

38 0 97 0 3 0

39 0 0 94 0 6

40 0 0 3 97 0

41 6 0 0 94 0

42 0 0 100 0 0

43 0 6 91 3 0

44 97 0 0 0 3

45 100 0 0 0 0

46 92 0 6 0 2

47 0 97 0 3 0

48 0 0 100 0 0

49 0 0 100 0 0

50 0 0 97 3 0

51 83 0 3 11 3

52 0 0 0 0 100

53 0 0 0 97 3

54 0 0 0 97 3

55 0 0 0 100 0

56 0 83 0 14 3

57 3 6 0 89 2

58 0 0 0 100 0

59 0 97 0 0 3

60 75 11 8 6 0

61 3 3 0 94 0

62 61 3 22 14 0

63 0 3 5 92 0

64 83 6 3 8 0

65 94 6 0 0 0

66 17 22 8 53 0

67 0 14 69 17 0

68 0 0 0 3 97

69 3 89 0 8 0

70 3 8 0 89 0

71 89 3 2 6 0

72 0 8 92 0 0

73 97 0 3 0 0

74 6 75 6 11 2

75 92 0 5 3 0

76 6 0 92 0 2

77 89 0 5 6 0

78 86 0 11 3 0

79 3 3 94 0 0

80 97 0 0 0 3

81 89 3 5 3 0

82 0 0 3 97 0

83 3 0 0 97 0

84 0 94 0 6 0

85 0 8 0 92 0

86 3 94 3 0 0

87 94 3 0 3 0

88 6 0 94 0 0

89 25 0 0 75 0

90 78 11 11 0 0

91 89 3 5 3 0

92 14 83 3 0 0

93 0 3 3 94 0

94 6 8 3 83 0

95 5 89 0 6 0

96 3 14 0 83 0

97 6 0 0 94 0

98 0 3 0 97 0

99 0 0 94 6 0

100 3 0 6 86 5

101 0 3 94 3 0

102 28 6 6 53 7

103 0 0 6 94 0

104 0 0 100 0 0

105 0 3 0 97 0

106 3 3 86 8 0

107 0 0 3 97 0

108 3 3 94 0 0

109 2 31 0 67 0

110 92 0 5 3 0

111 0 0 0 100 0

112 0 0 3 89 8

113 0 100 0 0 0

114 94 0 6 0 0

115 94 0 3 3 0

116 3 78 16 3 0

117 3 0 3 94 0

118 0 100 0 0 0

119 97 0 0 3 0

120 6 86 5 3 0

121 94 6 0 0 0

122 0 0 100 0 0

123 89 3 2 6 0

124 3 0 94 3 0

125 6 0 5 89 0

126 0 3 30 67 0

127 6 8 86 0 0

128 97 3 0 0 0

129 92 0 3 3 2

130 0 92 5 3 0

131 14 53 19 14 0

132 6 0 2 92 0

133 0 8 0 92 0

134 3 86 3 8 0

135 3 97 0 0 0

136 0 6 2 92 0

137 0 8 0 92 0

138 0 3 3 94 0

139 69 0 8 19 4

140 0 11 81 6 2

141 92 0 0 6 2

142 8 53 11 22 6

143 97 0 3 0 0

144 0 8 92 0 0

145 94 0 0 3 3

146 0 0 100 0 0

147 5 92 0 3 0

148 94 0 6 0 0

149 3 0 97 0 0

150 58 0 0 39 3

151 0 6 5 89 0

152 0 0 14 86 0

153 0 0 19 78 3

154 0 6 91 3 0

155 94 0 0 6 0

156 92 0 8 0 0

157 100 0 0 0 0

158 8 92 0 0 0

159 86 6 2 6 0

160 0 97 0 3 0

161 3 8 0 86 3

162 0 75 19 0 6

163 0 6 0 94 0

164 3 0 8 89 0

165 0 3 5 92 0

166 0 11 0 89 0

167 0 0 0 100 0

168 0 3 94 3 0

169 0 6 0 94 0

170 28 0 69 0 3

171 6 8 83 0 3

PERCON consensus matrix of alpha satellite D1 monomer

# A C G T -

1 89 0 5 0 6

2 89 0 5 6 0

3 28 0 0 72 0

4 16 79 0 5 0

5 0 0 0 100 0

6 0 0 100 0 0

7 5 79 0 16 0

8 100 0 0 0 0

9 79 0 21 0 0

10 5 0 84 5 6

11 11 0 0 89 0

12 0 0 95 5 0

13 11 0 84 5 0

14 95 5 0 0 0

15 0 0 0 100 0

16 100 0 0 0 0

17 5 5 0 89 1

18 5 0 5 89 1

19 0 5 0 95 0

20 0 0 95 5 0

21 5 11 84 0 0

22 74 21 0 5 0

23 0 0 0 100 0

24 89 0 5 5 1

25 0 11 89 0 0

26 5 79 5 11 0

27 0 11 0 89 0

28 0 26 0 74 0

29 5 0 11 84 0

30 16 0 79 0 5

31 89 0 5 5 1

32 5 11 73 11 0

33 0 0 95 5 0

34 95 0 0 0 5

35 0 0 0 100 0

36 0 0 0 100 0

37 0 0 0 100 0

38 0 100 0 0 0

39 0 0 100 0 0

40 0 5 0 95 0

41 0 0 5 95 0

42 0 0 100 0 0

43 0 0 100 0 0

44 100 0 0 0 0

45 84 5 11 0 0

46 68 0 21 0 11

47 0 100 0 0 0

48 0 0 100 0 0

49 0 0 100 0 0

50 0 0 95 0 5

51 100 0 0 0 0

52 68 0 0 26 6

53 0 0 11 89 0

54 58 0 10 32 0

55 0 0 0 47 53

56 0 47 0 0 53

57 16 0 5 26 53

58 0 0 0 47 53

59 0 100 0 0 0

60 95 0 5 0 0

61 16 5 11 68 0

62 74 21 5 0 0

63 5 11 5 74 5

64 100 0 0 0 0

65 95 0 5 0 0

66 95 0 0 5 0

67 79 5 5 11 0

68 21 5 11 63 0

69 0 63 37 0 0

70 5 21 0 68 6

71 100 0 0 0 0

72 5 5 89 0 1

73 95 0 0 5 0

74 0 84 16 0 0

75 84 0 5 11 0

76 0 0 100 0 0

77 58 42 0 0 0

78 100 0 0 0 0

79 0 0 100 0 0

80 0 100 0 0 0

81 68 11 21 0 0

82 0 21 0 79 0

83 5 0 0 95 0

84 11 89 0 0 0

85 0 0 0 100 0

86 0 79 16 5 0

87 95 0 5 0 0

88 5 0 95 0 0

89 79 0 21 0 0

90 100 0 0 0 0

91 95 0 5 0 0

92 11 68 5 16 0

93 11 5 0 84 0

94 16 0 16 68 0

95 5 95 0 0 0

96 0 5 0 95 0

97 0 11 0 89 0

98 5 0 0 95 0

99 0 21 79 0 0

100 0 0 11 89 0

101 0 0 100 0 0

102 100 0 0 0 0

103 0 0 0 100 0

104 21 0 79 0 0

105 5 5 0 89 1

106 0 37 0 63 0

107 0 0 0 100 0

108 0 0 100 0 0

109 0 89 0 11 0

110 95 0 0 5 0

111 0 0 0 100 0

112 0 0 0 100 0

113 0 89 11 0 0

114 100 0 0 0 0

115 74 5 16 5 0

116 5 26 68 0 1

117 11 0 0 89 0

118 0 100 0 0 0

119 100 0 0 0 0

120 5 63 5 26 1

121 95 0 5 0 0

122 0 0 95 0 5

123 100 0 0 0 0

124 16 0 84 0 0

125 0 5 0 95 0

126 11 0 0 89 0

127 0 0 100 0 0

128 100 0 0 0 0

129 100 0 0 0 0

130 0 68 16 16 0

131 100 0 0 0 0

132 0 5 0 95 0

133 0 0 0 100 0

134 0 100 0 0 0

135 0 89 11 0 0

136 0 95 0 5 0

137 0 0 0 100 0

138 11 0 0 89 0

139 5 5 0 79 11

140 5 79 11 5 0

141 63 0 16 21 0

142 5 5 0 89 1

143 95 0 0 5 0

144 0 5 95 0 0

145 89 0 11 0 0

146 5 0 95 0 0

147 0 95 0 5 0

148 95 5 0 0 0

149 0 16 79 5 0

150 0 0 95 5 0

151 0 11 0 89 0

152 0 5 0 95 0

153 0 0 11 84 5

154 0 0 100 0 0

155 95 0 5 0 0

156 100 0 0 0 0

157 89 0 0 11 0

158 5 89 5 0 1

159 95 5 0 0 0

160 0 95 5 0 0

161 0 0 0 100 0

162 5 95 0 0 0

163 0 11 0 84 5

164 0 0 0 95 5

165 0 5 0 89 6

166 0 32 0 63 5

167 0 5 11 74 10

168 0 5 95 0 0

169 5 5 16 74 0

170 95 0 5 0 0

171 5 16 79 0 0

PERCON consensus matrix of alpha satellite D2 monomer

# A C G T -

1 5 5 0 89 1

2 79 0 21 0 0

3 0 11 0 89 0

4 5 79 11 5 0

5 0 0 0 100 0

6 5 0 95 0 0

7 5 5 79 11 0

8 100 0 0 0 0

9 84 0 5 11 0

10 11 5 84 0 0

11 0 5 0 89 6

12 0 0 100 0 0

13 5 0 95 0 0

14 79 0 16 0 5

15 0 95 0 5 0

16 79 0 16 5 0

17 0 0 0 100 0

18 0 0 0 100 0

19 5 0 0 95 0

20 0 32 68 0 0

21 11 5 84 0 0

22 95 0 5 0 0

23 11 5 79 5 0

24 5 95 0 0 0

25 11 0 84 5 0

26 5 89 5 0 1

27 0 0 0 95 5

28 0 0 0 95 5

29 11 0 0 89 0

30 0 53 47 0 0

31 89 11 0 0 0

32 0 0 74 26 0

33 0 0 95 5 0

34 5 79 5 5 6

35 0 100 0 0 0

36 0 11 0 89 0

37 84 0 0 16 0

38 16 16 0 68 0

39 0 0 95 5 0

40 0 0 74 26 0

41 5 0 0 95 0

42 0 0 84 0 16

43 68 0 26 5 1

44 79 0 16 5 0

45 100 0 0 0 0

46 100 0 0 0 0

47 89 0 11 0 0

48 5 0 95 0 0

49 0 0 89 11 0

50 84 0 16 0 0

51 100 0 0 0 0

52 100 0 0 0 0

53 0 5 0 95 0

54 86 0 14 0 0

55 0 5 0 95 0

56 0 100 0 0 0

57 0 0 0 100 0

58 0 0 0 100 0

59 5 90 0 5 0

60 19 71 0 10 0

61 19 76 0 0 5

62 81 10 9 0 0

63 0 0 0 100 0

64 100 0 0 0 0

65 95 5 0 0 0

66 81 19 0 0 0

67 100 0 0 0 0

68 90 0 10 0 0

69 0 100 0 0 0

70 0 14 19 67 0

71 90 0 10 0 0

72 0 0 95 5 0

73 95 0 5 0 0

74 10 90 0 0 0

75 100 0 0 0 0

76 0 0 100 0 0

77 100 0 0 0 0

78 100 0 0 0 0

79 0 0 95 5 0

80 0 86 14 0 0

81 95 5 0 0 0

82 0 0 5 95 0

83 0 0 5 95 0

84 0 100 0 0 0

85 0 0 0 100 0

86 0 90 10 0 0

87 90 0 5 5 0

88 0 0 95 5 0

89 95 0 0 0 5

90 100 0 0 0 0

91 100 0 0 0 0

92 0 100 0 0 0

93 10 0 4 86 0

94 19 0 5 76 0

95 33 19 48 0 0

96 0 14 5 81 0

97 0 0 0 100 0

98 0 10 0 90 0

99 5 5 90 0 0

100 9 5 0 86 0

101 0 0 100 0 0

102 90 5 0 5 0

103 5 0 0 95 0

104 0 0 100 0 0

105 0 0 0 100 0

106 5 0 95 0 0

107 0 0 0 100 0

108 0 5 95 0 0

109 5 10 4 81 0

110 62 33 0 5 0

111 5 90 0 5 0

112 0 14 0 86 0

113 0 100 0 0 0

114 90 0 5 5 0

115 71 0 24 5 0

116 0 86 14 0 0

117 0 5 0 95 0

118 90 5 5 0 0

119 100 0 0 0 0

120 24 62 14 0 0

121 81 0 19 0 0

122 5 0 95 0 0

123 95 0 0 5 0

124 10 0 90 0 0

125 0 0 5 95 0

126 10 0 9 81 0

127 10 5 85 0 0

128 90 0 10 0 0

129 100 0 0 0 0

130 0 95 0 5 0

131 19 81 0 0 0

132 10 0 0 90 0

133 0 14 0 86 0

134 0 24 0 76 0

135 0 81 14 5 0

136 0 0 0 100 0

137 14 5 5 76 0

138 5 5 0 90 0

139 5 0 0 95 0

140 0 19 62 14 5

141 90 0 5 5 0

142 9 24 0 67 0

143 81 0 19 0 0

144 0 24 76 0 0

145 90 5 5 0 0

146 10 0 90 0 0

147 10 86 0 0 4

148 95 5 0 0 0

149 5 5 80 10 0

150 0 5 5 90 0

151 0 0 0 100 0

152 5 5 0 90 0

153 0 0 19 81 0

154 5 0 90 5 0

155 95 0 5 0 0

156 100 0 0 0 0

157 95 0 5 0 0

158 5 90 0 5 0

159 81 0 14 5 0

160 0 100 0 0 0

161 5 0 0 95 0

162 5 95 0 0 0

163 0 5 0 90 5

164 0 0 5 95 0

165 0 0 0 100 0

166 0 0 0 95 5

167 0 0 0 95 5

168 5 10 85 0 0

169 0 0 0 100 0

170 38 0 52 5 5

171 14 5 81 0 0

PERCON consensus matrix of alpha satellite W1 monomer

# A C G T -

1 87 0 6 7 0

2 100 0 0 0 0

3 13 0 0 87 0

4 13 80 7 0 0

5 0 0 0 100 0

6 7 0 93 0 0

7 0 100 0 0 0

8 93 0 7 0 0

9 73 7 20 0 0

10 0 0 93 7 0

11 0 0 7 93 0

12 7 0 93 0 0

13 0 7 93 0 0

14 100 0 0 0 0

15 0 0 0 100 0

16 100 0 0 0 0

17 0 0 0 100 0

18 0 0 0 100 0

19 0 7 6 87 0

20 7 0 93 0 0

21 0 0 93 7 0

22 87 0 6 7 0

23 0 93 7 0 0

24 0 67 0 27 6

25 0 0 7 93 0

26 0 100 0 0 0

27 0 0 0 100 0

28 0 93 0 7 0

29 0 0 0 100 0

30 0 7 93 0 0

31 93 0 0 7 0

32 0 0 100 0 0

33 20 7 73 0 0

34 93 0 7 0 0

35 7 0 6 87 0

36 0 0 0 100 0

37 0 7 0 93 0

38 0 93 0 7 0

39 0 0 100 0 0

40 0 0 0 100 0

41 0 0 0 100 0

42 0 0 100 0 0

43 0 7 93 0 0

44 100 0 0 0 0

45 93 0 0 7 0

46 100 0 0 0 0

47 0 93 0 7 0

48 0 0 100 0 0

49 0 0 100 0 0

50 7 0 93 0 0

51 93 0 0 7 0

52 13 0 7 80 0

53 86 7 0 7 0

54 100 0 0 0 0

55 67 13 0 0 20

56 7 80 0 13 0

57 0 33 7 60 0

58 0 0 27 73 0

59 0 100 0 0 0

60 60 40 0 0 0

61 0 93 7 0 0

62 67 27 6 0 0

63 0 0 47 53 0

64 93 0 7 0 0

65 93 0 0 7 0

66 0 0 0 0 100

67 0 0 0 0 100

68 0 0 0 0 100

69 0 100 0 0 0

70 0 0 0 100 0

71 80 0 20 0 0

72 73 27 0 0 0

73 100 0 0 0 0

74 7 80 13 0 0

75 60 0 40 0 0

76 0 7 86 7 0

77 93 0 7 0 0

78 100 0 0 0 0

79 20 0 80 0 0

80 0 100 0 0 0

81 93 0 0 0 7

82 13 7 7 73 0

83 7 0 6 87 0

84 0 93 7 0 0

85 0 7 6 87 0

86 0 80 20 0 0

87 93 7 0 0 0

88 0 0 100 0 0

89 100 0 0 0 0

90 93 0 0 0 7

91 53 33 0 7 7

92 7 87 6 0 0

93 6 7 0 87 0

94 0 0 0 100 0

95 0 100 0 0 0

96 0 0 0 100 0

97 0 0 0 100 0

98 0 33 7 60 0

99 7 7 86 0 0

100 0 0 0 100 0

101 0 7 93 0 0

102 100 0 0 0 0

103 0 13 0 87 0

104 0 0 100 0 0

105 7 7 0 80 6

106 6 7 0 87 0

107 0 0 7 93 0

108 0 7 93 0 0

109 0 93 0 7 0

110 100 0 0 0 0

111 0 0 0 100 0

112 7 0 0 93 0

113 0 93 0 7 0

114 100 0 0 0 0

115 100 0 0 0 0

116 20 80 0 0 0

117 0 0 7 93 0

118 0 80 13 7 0

119 73 20 7 0 0

120 0 100 0 0 0

121 93 7 0 0 0

122 0 0 100 0 0

123 73 0 0 27 0

124 0 0 100 0 0

125 7 7 6 80 0

126 0 0 7 93 0

127 0 0 100 0 0

128 100 0 0 0 0

129 100 0 0 0 0

130 0 100 0 0 0

131 0 93 0 7 0

132 0 0 0 100 0

133 0 0 7 93 0

134 0 60 13 27 0

135 0 93 0 7 0

136 0 13 0 87 0

137 0 7 0 93 0

138 0 0 0 73 27

139 0 0 0 27 73

140 0 33 67 0 0

141 87 0 0 13 0

142 7 0 20 73 0

143 100 0 0 0 0

144 0 0 100 0 0

145 0 0 0 100 0

146 0 0 0 100 0

147 0 100 0 0 0

148 93 7 0 0 0

149 0 13 87 0 0

150 7 27 66 0 0

151 0 0 0 93 7

152 0 0 0 100 0

153 0 7 0 93 0

154 0 20 80 0 0

155 86 7 0 7 0

156 87 0 6 7 0

157 93 0 0 7 0

158 0 93 0 7 0

159 80 0 20 0 0

160 0 87 13 0 0

161 0 13 0 87 0

162 7 93 0 0 0

163 0 13 0 87 0

164 13 0 0 87 0

165 0 0 13 87 0

166 0 20 0 80 0

167 7 13 7 73 0

168 0 7 86 7 0

169 0 20 7 73 0

170 87 0 13 0 0

171 0 0 100 0 0

PERCON consensus matrix of alpha satellite W2 monomer

# A C G T -

1 67 0 33 0 0

2 100 0 0 0 0

3 0 11 0 89 0

4 0 100 0 0 0

5 0 0 0 100 0

6 11 0 89 0 0

7 0 100 0 0 0

8 100 0 0 0 0

9 89 0 11 0 0

10 0 0 100 0 0

11 0 0 0 100 0

12 0 0 100 0 0

13 0 0 89 11 0

14 100 0 0 0 0

15 0 0 0 100 0

16 100 0 0 0 0

17 0 0 0 100 0

18 0 0 0 100 0

19 0 0 0 100 0

20 0 0 89 11 0

21 0 0 100 0 0

22 100 0 0 0 0

23 0 67 11 11 11

24 0 100 0 0 0

25 100 0 0 0 0

26 0 100 0 0 0

27 0 0 0 100 0

28 0 22 11 67 0

29 0 0 11 89 0

30 0 0 100 0 0

31 22 0 0 78 0

32 11 0 78 0 11

33 0 0 89 11 0

34 0 100 0 0 0

35 0 100 0 0 0

36 0 0 0 100 0

37 0 0 0 100 0

38 0 100 0 0 0

39 0 44 56 0 0

40 0 0 0 100 0

41 0 0 0 100 0

42 0 89 0 11 0

43 0 0 100 0 0

44 100 0 0 0 0

45 100 0 0 0 0

46 100 0 0 0 0

47 0 100 0 0 0

48 0 0 89 0 11

49 0 0 89 11 0

50 0 11 89 0 0

51 0 0 0 100 0

52 100 0 0 0 0

53 0 0 0 100 0

54 100 0 0 0 0

55 0 11 0 89 0

56 0 89 11 0 0

57 0 11 0 89 0

58 0 0 0 100 0

59 0 100 0 0 0

60 89 0 11 0 0

61 0 100 0 0 0

62 89 0 11 0 0

63 0 0 0 100 0

64 33 56 11 0 0

65 89 11 0 0 0

66 89 11 0 0 0

67 100 0 0 0 0

68 11 56 0 33 0

69 0 100 0 0 0

70 0 0 0 100 0

71 100 0 0 0 0

72 11 0 89 0 0

73 100 0 0 0 0

74 0 89 11 0 0

75 89 11 0 0 0

76 0 0 100 0 0

77 100 0 0 0 0

78 100 0 0 0 0

79 11 0 89 0 0

80 0 100 0 0 0

81 78 22 0 0 0

82 0 0 0 100 0

83 0 11 0 89 0

84 0 100 0 0 0

85 0 0 0 100 0

86 0 100 0 0 0

87 100 0 0 0 0

88 0 0 100 0 0

89 100 0 0 0 0

90 100 0 0 0 0

91 44 0 11 44 1

92 0 44 56 0 0

93 0 0 0 100 0

94 0 0 0 100 0

95 0 44 0 56 0

96 0 44 0 56 0

97 0 89 11 0 0

98 11 0 0 89 0

99 0 0 100 0 0

100 0 11 0 89 0

101 0 0 100 0 0

102 100 0 0 0 0

103 0 0 0 100 0

104 0 0 100 0 0

105 100 0 0 0 0

106 0 67 11 22 0

107 0 0 0 100 0

108 0 11 89 0 0

109 0 100 0 0 0

110 100 0 0 0 0

111 0 0 0 100 0

112 0 0 0 100 0

113 0 100 0 0 0

114 89 11 0 0 0

115 100 0 0 0 0

116 0 89 11 0 0

117 0 0 11 89 0

118 0 100 0 0 0

119 100 0 0 0 0

120 0 100 0 0 0

121 89 0 11 0 0

122 0 11 89 0 0

123 100 0 0 0 0

124 0 0 100 0 0

125 0 0 33 67 0

126 0 0 0 100 0

127 0 0 100 0 0

128 100 0 0 0 0

129 100 0 0 0 0

130 0 100 0 0 0

131 67 22 0 11 0

132 56 33 0 11 0

133 0 0 0 100 0

134 0 89 0 11 0

135 0 100 0 0 0

136 0 0 0 100 0

137 11 0 44 44 1

138 0 33 0 67 0

139 0 0 11 89 0

140 0 0 100 0 0

141 100 0 0 0 0

142 0 0 11 89 0

143 33 0 67 0 0

144 0 0 100 0 0

145 89 11 0 0 0

146 0 0 100 0 0

147 0 100 0 0 0

148 100 0 0 0 0

149 0 0 100 0 0

150 0 0 0 100 0

151 0 0 11 89 0

152 0 0 0 100 0

153 0 0 0 100 0

154 0 0 100 0 0

155 100 0 0 0 0

156 100 0 0 0 0

157 100 0 0 0 0

158 0 100 0 0 0

159 0 11 11 78 0

160 0 89 11 0 0

161 0 0 0 100 0

162 0 100 0 0 0

163 0 0 0 100 0

164 0 0 0 100 0

165 0 0 0 100 0

166 0 67 0 33 0

167 0 0 0 100 0

168 0 11 33 56 0

169 0 0 0 100 0

170 22 0 78 0 0

171 0 0 100 0 0

PERCON consensus matrix of alpha satellite W3 monomer

# A C G T -

1 75 17 8 0 0

2 67 0 0 33 0

3 0 0 0 100 0

4 0 100 0 0 0

5 0 8 0 92 0

6 0 0 100 0 0

7 0 92 0 8 0

8 100 0 0 0 0

9 100 0 0 0 0

10 8 0 92 0 0

11 0 8 17 67 8

12 0 0 100 0 0

13 0 0 100 0 0

14 92 0 0 0 8

15 0 8 0 83 9

16 92 0 0 0 8

17 0 8 0 83 9

18 8 0 75 17 0

19 0 0 0 100 0

20 8 0 92 0 0

21 0 0 100 0 0

22 100 0 0 0 0

23 8 92 0 0 0

24 0 92 8 0 0

25 0 0 0 100 0

26 0 92 0 8 0

27 0 0 0 100 0

28 0 8 33 58 1

29 0 8 0 92 0

30 0 0 100 0 0

31 100 0 0 0 0

32 100 0 0 0 0

33 0 8 92 0 0

34 92 0 8 0 0

35 0 0 0 100 0

36 0 0 8 92 0

37 0 0 0 100 0

38 0 100 0 0 0

39 8 8 58 25 1

40 8 8 0 83 1

41 0 0 0 100 0

42 0 0 100 0 0

43 0 0 100 0 0

44 100 0 0 0 0

45 100 0 0 0 0

46 92 0 0 8 0

47 8 92 0 0 0

48 0 0 100 0 0

49 0 0 100 0 0

50 8 0 92 0 0

51 67 0 0 33 0

52 25 0 8 67 0

53 25 50 0 25 0

54 92 8 0 0 0

55 0 0 0 100 0

56 0 92 0 0 8

57 0 0 0 100 0

58 0 8 0 92 0

59 0 100 0 0 0

60 92 8 0 0 0

61 0 100 0 0 0

62 83 8 8 0 1

63 0 0 42 58 0

64 83 0 17 0 0

65 100 0 0 0 0

66 100 0 0 0 0

67 100 0 0 0 0

68 75 0 25 0 0

69 0 83 17 0 0

70 0 0 0 100 0

71 100 0 0 0 0

72 92 8 0 0 0

73 100 0 0 0 0

74 0 100 0 0 0

75 75 0 17 8 0

76 0 0 100 0 0

77 100 0 0 0 0

78 92 0 0 8 0

79 0 0 100 0 0

80 0 100 0 0 0

81 100 0 0 0 0

82 0 0 0 100 0

83 0 0 8 92 0

84 0 100 0 0 0

85 0 0 0 100 0

86 0 100 0 0 0

87 92 0 8 0 0

88 0 0 100 0 0

89 83 0 17 0 0

90 100 0 0 0 0

91 100 0 0 0 0

92 0 92 8 0 0

93 0 0 0 100 0

94 25 8 25 42 0

95 0 83 0 17 0

96 0 0 0 100 0

97 0 8 0 92 0

98 0 0 8 92 0

99 0 0 100 0 0

100 0 0 8 92 0

101 0 0 100 0 0

102 92 0 8 0 0

103 0 0 8 92 0

104 0 0 100 0 0

105 0 17 0 83 0

106 0 0 0 100 0

107 0 0 0 100 0

108 0 0 100 0 0

109 0 25 0 75 0

110 42 0 58 0 0

111 0 0 0 100 0

112 0 0 0 100 0

113 0 100 0 0 0

114 58 33 8 0 1

115 92 8 0 0 0

116 0 83 8 8 1

117 0 8 0 92 0

118 0 67 0 33 0

119 25 67 8 0 0

120 33 67 0 0 0

121 100 0 0 0 0

122 0 0 100 0 0

123 100 0 0 0 0

124 33 0 67 0 0

125 0 0 0 100 0

126 8 0 0 92 0

127 8 0 67 25 0

128 83 8 0 8 1

129 100 0 0 0 0

130 8 83 8 0 1

131 25 0 0 75 0

132 0 0 0 100 0

133 0 0 0 100 0

134 0 75 17 8 0

135 0 100 0 0 0

136 0 8 0 92 0

137 0 25 8 67 0

138 0 0 0 100 0

139 0 8 8 83 1

140 0 25 75 0 0

141 100 0 0 0 0

142 17 33 8 42 0

143 100 0 0 0 0

144 0 0 100 0 0

145 92 8 0 0 0

146 0 0 100 0 0

147 8 83 0 8 1

148 100 0 0 0 0

149 0 0 100 0 0

150 0 67 0 33 0

151 0 0 0 100 0

152 17 42 0 33 8

153 0 0 0 100 0

154 0 0 100 0 0

155 100 0 0 0 0

156 92 8 0 0 0

157 100 0 0 0 0

158 0 92 0 8 0

159 58 42 0 0 0

160 0 75 0 25 0

161 0 0 8 83 9

162 0 92 0 0 8

163 0 0 0 100 0

164 0 8 0 92 0

165 0 0 0 100 0

166 0 0 0 100 0

167 0 17 8 75 0

168 0 75 25 0 0

169 0 17 8 75 0

170 100 0 0 0 0

171 0 8 92 0 0

PERCON consensus matrix of alpha satellite W4 monomer

# A C G T -

1 83 8 0 8 1

2 83 0 17 0 0

3 0 0 0 100 0

4 0 92 8 0 0

5 0 8 0 92 0

6 8 25 67 0 0

7 0 100 0 0 0

8 100 0 0 0 0

9 92 0 8 0 0

10 0 0 100 0 0

11 0 0 8 92 0

12 0 0 100 0 0

13 0 0 100 0 0

14 92 8 0 0 0

15 0 92 0 8 0

16 83 0 17 0 0

17 0 0 0 100 0

18 0 0 0 100 0

19 0 0 0 100 0

20 8 0 92 0 0

21 0 0 100 0 0

22 92 8 0 0 0

23 0 0 100 0 0

24 0 33 67 0 0

25 0 0 100 0 0

26 0 100 0 0 0

27 0 0 0 100 0

28 0 0 0 100 0

29 0 8 8 83 1

30 0 25 75 0 0

31 83 0 0 17 0

32 0 0 100 0 0

33 0 0 100 0 0

34 17 75 0 8 0

35 0 83 0 8 9

36 0 0 0 100 0

37 0 0 100 0 0

38 0 0 8 92 0

39 0 8 92 0 0

40 0 8 83 8 1

41 0 0 0 100 0

42 0 8 92 0 0

43 0 8 92 0 0

44 100 0 0 0 0

45 100 0 0 0 0

46 100 0 0 0 0

47 100 0 0 0 0

48 0 8 92 0 0

49 0 0 100 0 0

50 83 8 8 0 1

51 92 0 0 8 0

52 75 0 0 25 0

53 8 0 0 83 9

54 92 0 0 8 0

55 0 8 0 92 0

56 0 92 8 0 0

57 0 0 8 92 0

58 0 0 8 92 0

59 0 100 0 0 0

60 67 8 0 25 0

61 0 100 0 0 0

62 75 17 8 0 0

63 8 8 8 75 1

64 92 0 0 8 0

65 100 0 0 0 0

66 100 0 0 0 0

67 83 0 8 8 1

68 75 8 17 0 0

69 0 100 0 0 0

70 0 8 17 75 0

71 92 0 8 0 0

72 0 25 75 0 0

73 100 0 0 0 0

74 0 0 25 75 0

75 75 0 25 0 0

76 0 0 100 0 0

77 100 0 0 0 0

78 100 0 0 0 0

79 0 0 100 0 0

80 0 100 0 0 0

81 100 0 0 0 0

82 0 8 0 92 0

83 0 0 0 100 0

84 8 75 17 0 0

85 0 0 0 100 0

86 0 100 0 0 0

87 100 0 0 0 0

88 0 0 100 0 0

89 100 0 0 0 0

90 100 0 0 0 0

91 100 0 0 0 0

92 0 100 0 0 0

93 0 0 17 83 0

94 50 0 25 25 0

95 0 100 0 0 0

96 0 0 0 100 0

97 0 17 0 83 0

98 0 0 0 100 0

99 0 0 100 0 0

100 8 0 8 83 1

101 0 0 100 0 0

102 100 0 0 0 0

103 0 0 8 92 0

104 0 0 100 0 0

105 100 0 0 0 0

106 0 0 0 100 0

107 8 0 8 83 1

108 0 0 100 0 0

109 0 100 0 0 0

110 100 0 0 0 0

111 0 8 0 92 0

112 0 0 0 100 0

113 8 92 0 0 0

114 75 0 25 0 0

115 92 8 0 0 0

116 0 83 17 0 0

117 0 0 0 100 0

118 8 83 8 0 1

119 83 17 0 0 0

120 0 92 0 8 0

121 83 0 17 0 0

122 0 8 92 0 0

123 100 0 0 0 0

124 0 8 92 0 0

125 0 0 0 100 0

126 0 0 0 100 0

127 0 0 100 0 0

128 92 0 8 0 0

129 92 0 0 0 8

130 0 75 25 0 0

131 83 8 8 0 1

132 8 0 0 92 0

133 0 0 0 100 0

134 0 100 0 0 0

135 0 100 0 0 0

136 8 17 0 75 0

137 42 0 0 58 0

138 0 0 0 100 0

139 17 0 8 67 8

140 0 8 92 0 0

141 100 0 0 0 0

142 17 8 8 67 0

143 100 0 0 0 0

144 0 17 83 0 0

145 100 0 0 0 0

146 0 0 100 0 0

147 0 100 0 0 0

148 92 8 0 0 0

149 0 0 100 0 0

150 8 0 17 75 0

151 0 0 0 100 0

152 0 0 0 100 0

153 0 17 83 0 0

154 0 17 58 25 0

155 100 0 0 0 0

156 100 0 0 0 0

157 83 0 0 17 0

158 0 100 0 0 0

159 92 0 0 8 0

160 17 83 0 0 0

161 8 0 0 92 0

162 0 100 0 0 0

163 0 0 0 100 0

164 0 0 0 100 0

165 0 0 8 92 0

166 0 25 17 58 0

167 0 0 17 83 0

168 0 0 100 0 0

169 0 0 0 92 8

170 58 8 25 8 1

171 0 0 100 0 0

PERCON consensus matrix of alpha satellite W5 monomer

# A C G T -

1 86 7 0 7 0

2 100 0 0 0 0

3 7 0 0 93 0

4 0 93 7 0 0

5 0 0 0 100 0

6 0 0 100 0 0

7 7 86 0 7 0

8 71 0 21 7 1

9 93 0 0 7 0

10 7 0 71 21 1

11 0 0 14 86 0

12 0 0 100 0 0

13 7 7 86 0 0

14 93 0 0 7 0

15 0 0 93 7 0

16 93 7 0 0 0

17 7 0 0 93 0

18 7 0 0 93 0

19 0 0 0 100 0

20 0 0 100 0 0

21 0 7 93 0 0

22 100 0 0 0 0

23 14 71 7 0 8

24 0 71 0 29 0

25 0 0 79 21 0

26 0 100 0 0 0

27 7 0 0 93 0

28 7 0 0 93 0

29 0 0 0 100 0

30 0 0 93 0 7

31 100 0 0 0 0

32 0 0 100 0 0

33 0 0 100 0 0

34 7 86 0 0 7

35 0 93 7 0 0

36 0 0 0 100 0

37 86 0 14 0 0

38 0 50 0 50 0

39 0 21 64 14 1

40 0 0 100 0 0

41 0 7 0 93 0

42 86 7 7 0 0

43 0 7 93 0 0

44 0 7 0 93 0

45 93 0 7 0 0

46 86 7 7 0 0

47 93 0 0 7 0

48 0 0 100 0 0

49 0 0 100 0 0

50 86 0 14 0 0

51 93 0 0 7 0

52 93 0 7 0 0

53 0 14 29 57 0

54 100 0 0 0 0

55 71 0 29 0 0

56 0 100 0 0 0

57 0 0 0 100 0

58 0 0 7 93 0

59 0 100 0 0 0

60 86 14 0 0 0

61 0 7 0 93 0

62 50 36 14 0 0

63 0 7 7 86 0

64 100 0 0 0 0

65 100 0 0 0 0

66 93 0 7 0 0

67 100 0 0 0 0

68 79 0 14 7 0

69 0 86 14 0 0

70 21 36 0 43 0

71 86 0 14 0 0

72 36 0 64 0 0

73 100 0 0 0 0

74 0 100 0 0 0

75 64 0 36 0 0

76 0 0 100 0 0

77 71 0 0 29 0

78 100 0 0 0 0

79 0 0 100 0 0

80 0 100 0 0 0

81 93 0 0 7 0

82 0 14 0 86 0

83 0 0 0 100 0

84 0 100 0 0 0

85 29 7 0 64 0

86 0 100 0 0 0

87 100 0 0 0 0

88 0 0 100 0 0

89 93 0 7 0 0

90 86 14 0 0 0

91 93 0 0 7 0

92 100 0 0 0 0

93 0 14 0 86 0

94 29 0 0 71 0

95 0 100 0 0 0

96 0 0 0 100 0

97 0 0 0 100 0

98 14 0 14 71 1

99 0 0 93 7 0

100 0 7 14 79 0

101 0 0 100 0 0

102 100 0 0 0 0

103 0 29 0 71 0

104 7 14 79 0 0

105 100 0 0 0 0

106 0 7 0 93 0

107 0 0 21 79 0

108 0 0 100 0 0

109 79 14 7 0 0

110 21 0 79 0 0

111 0 0 0 100 0

112 0 0 0 100 0

113 0 14 29 57 0

114 93 7 0 0 0

115 93 0 0 7 0

116 0 100 0 0 0

117 0 0 0 100 0

118 21 79 0 0 0

119 100 0 0 0 0

120 0 79 21 0 0

121 93 0 7 0 0

122 0 0 100 0 0

123 100 0 0 0 0

124 0 0 100 0 0

125 0 100 0 0 0

126 7 0 0 93 0

127 0 0 100 0 0

128 100 0 0 0 0

129 79 0 21 0 0

130 0 100 0 0 0

131 93 7 0 0 0

132 0 0 7 93 0

133 7 0 21 71 1

134 0 79 7 14 0

135 0 93 7 0 0

136 0 0 0 100 0

137 0 0 0 100 0

138 7 0 7 86 0

139 29 7 35 29 0

140 0 0 100 0 0

141 86 0 14 0 0

142 0 0 0 100 0

143 0 0 100 0 0

144 7 0 86 7 0

145 79 21 0 0 0

146 0 0 100 0 0

147 0 86 0 7 7

148 100 0 0 0 0

149 0 7 86 7 0

150 7 0 7 86 0

151 0 0 0 100 0

152 0 0 0 100 0

153 7 71 7 14 1

154 21 43 36 0 0

155 100 0 0 0 0

156 93 0 7 0 0

157 100 0 0 0 0

158 0 93 0 7 0

159 93 7 0 0 0

160 0 100 0 0 0

161 93 0 0 7 0

162 0 100 0 0 0

163 0 0 14 86 0

164 7 0 0 93 0

165 0 0 0 100 0

166 0 36 0 64 0

167 0 0 7 93 0

168 0 7 93 0 0

169 0 14 0 86 0

170 100 0 0 0 0

171 0 7 93 0 0

PERCON consensus matrix of alpha satellite R1 monomer

# A C G T -

1 88 0 0 12 0

2 88 6 0 6 0

3 6 0 0 94 0

4 6 88 0 6 0

5 0 6 12 82 0

6 0 6 94 0 0

7 6 88 6 0 0

8 76 6 0 18 0

9 41 0 35 24 0

10 6 6 76 12 0

11 12 12 17 59 0

12 6 0 88 6 0

13 6 0 94 0 0

14 94 0 0 6 0

15 6 0 0 94 0

16 94 0 6 0 0

17 0 6 0 94 0

18 0 0 0 100 0

19 6 0 6 88 0

20 6 0 88 6 0

21 6 0 88 6 0

22 88 0 0 6 6

23 24 41 35 0 0

24 6 41 12 41 0

25 6 6 53 35 0

26 6 82 0 6 6

27 6 6 0 88 0

28 0 0 0 100 0

29 6 0 6 82 6

30 6 6 82 6 0

31 100 0 0 0 0

32 35 0 65 0 0

33 0 0 95 5 0

34 30 60 5 5 0

35 20 70 0 10 0

36 0 5 0 95 0

37 5 0 0 95 0

38 0 100 0 0 0

39 16 0 37 42 5

40 0 0 5 95 0

41 0 5 5 84 6

42 0 0 100 0 0

43 0 0 95 5 0

44 100 0 0 0 0

45 95 0 0 5 0

46 90 0 0 5 5

47 0 85 0 15 0

48 20 5 70 0 5

49 5 5 85 5 0

50 5 5 90 0 0

51 90 0 0 5 5

52 75 0 5 10 10

53 5 0 0 95 0

54 100 0 0 0 0

55 5 5 0 90 0

56 0 95 0 5 0

57 0 0 15 85 0

58 5 0 0 95 0

59 20 75 0 5 0

60 95 5 0 0 0

61 5 90 0 5 0

62 80 20 0 0 0

63 10 0 5 85 0

64 85 0 5 5 5

65 90 5 5 0 0

66 95 0 5 0 0

67 100 0 0 0 0

68 80 10 5 5 0

69 0 75 5 15 5

70 0 0 0 100 0

71 100 0 0 0 0

72 5 5 90 0 0

73 95 0 5 0 0

74 5 90 0 5 0

75 95 5 0 0 0

76 10 0 90 0 0

77 95 0 0 5 0

78 85 0 5 5 5

79 0 5 95 0 0

80 5 80 0 15 0

81 100 0 0 0 0

82 0 0 0 100 0

83 0 5 0 95 0

84 0 95 0 5 0

85 10 5 0 85 0

86 0 95 5 0 0

87 90 0 5 5 0

88 0 0 100 0 0

89 95 5 0 0 0

90 100 0 0 0 0

91 100 0 0 0 0

92 5 80 10 5 0

93 10 10 0 80 0

94 10 10 5 75 0

95 0 95 0 5 0

96 0 0 0 100 0

97 0 0 0 100 0

98 0 0 0 100 0

99 5 0 90 5 0

100 5 0 0 95 0

101 0 0 95 5 0

102 100 0 0 0 0

103 0 0 0 100 0

104 5 5 85 5 0

105 15 0 5 80 0

106 0 0 95 5 0

107 0 5 20 75 0

108 10 10 80 0 0

109 0 95 5 0 0

110 100 0 0 0 0

111 0 0 0 100 0

112 0 0 0 100 0

113 10 85 5 0 0

114 100 0 0 0 0

115 85 5 0 10 0

116 0 100 0 0 0

117 10 0 0 90 0

118 0 100 0 0 0

119 100 0 0 0 0

120 5 85 10 0 0

121 80 10 10 0 0

122 0 0 100 0 0

123 90 10 0 0 0

124 5 5 85 5 0

125 0 16 0 84 0

126 5 5 0 90 0

127 0 0 100 0 0

128 100 0 0 0 0

129 95 0 0 5 0

130 10 80 5 5 0

131 5 85 5 5 0

132 10 0 0 90 0

133 10 5 0 85 0

134 0 50 0 50 0

135 5 95 0 0 0

136 0 5 0 95 0

137 0 0 10 90 0

138 0 5 5 90 0

139 0 0 10 90 0

140 10 15 75 0 0

141 90 5 5 0 0

142 5 0 5 90 0

143 80 0 15 5 0

144 5 20 75 0 0

145 89 0 5 5 1

146 0 11 89 0 0

147 5 89 5 0 1

148 100 0 0 0 0

149 0 5 90 0 5

150 5 5 0 90 0

151 5 5 0 90 0

152 0 0 5 95 0

153 0 0 5 90 5

154 0 0 95 5 0

155 100 0 0 0 0

156 100 0 0 0 0

157 100 0 0 0 0

158 10 85 0 5 0

159 75 10 5 10 0

160 0 80 5 15 0

161 0 5 0 95 0

162 0 95 5 0 0

163 0 10 10 80 0

164 0 0 0 100 0

165 0 5 0 95 0

166 0 10 5 85 0

167 0 10 0 85 5

168 20 5 65 10 0

169 20 10 0 70 0

170 95 5 0 0 0

171 10 10 70 10 0

PERCON consensus matrix of alpha satellite R2 monomer (v2)

# A C G T -

1 91 1 4 3 1

2 95 1 2 1 1

3 3 2 1 93 1

4 1 86 1 11 1

5 1 1 1 96 1

6 5 2 87 5 1

7 5 89 2 3 1

8 93 4 1 1 1

9 89 1 7 2 1

10 7 2 86 4 1

11 5 2 2 90 1

12 4 1 90 4 1

13 3 5 82 9 1

14 90 1 4 4 1

15 1 37 1 60 1

16 93 1 4 1 1

17 3 3 1 92 1

18 2 2 3 92 1

19 2 2 1 94 1

20 11 2 76 10 1

21 3 5 86 5 1

22 84 1 2 12 1

23 3 5 78 13 1

24 8 65 7 19 1

25 13 14 64 8 1

26 4 85 1 9 1

27 3 1 1 94 1

28 1 2 1 95 1

29 1 1 2 95 1

30 4 3 88 4 1

31 89 1 5 4 1

32 8 1 84 6 1

33 4 1 90 4 1

34 5 83 3 8 1

35 3 87 1 8 1

36 5 3 1 90 1

37 67 3 22 7 1

38 7 7 1 84 1

39 7 1 86 5 1

40 5 1 75 18 1

41 3 3 1 92 1

42 25 1 69 4 1

43 15 2 80 2 1

44 94 1 3 1 1

45 95 1 1 2 1

46 94 1 1 2 2

47 90 1 3 5 1

48 3 9 81 6 1

49 8 4 85 2 1

50 90 1 5 3 1

51 91 1 3 4 1

52 91 1 5 2 1

53 1 2 1 95 1

54 95 2 1 1 1

55 3 4 1 91 1

56 2 92 2 3 1

57 1 4 1 93 1

58 2 1 1 95 1

59 1 93 1 4 1

60 91 4 2 2 1

61 4 71 2 22 1

62 93 2 2 2 1

63 3 3 2 90 2

64 87 1 5 5 2

65 85 1 12 1 1

66 95 1 2 1 1

67 96 1 1 1 1

68 94 1 2 2 1

69 3 82 9 5 1

70 4 5 2 88 1

71 92 1 4 2 1

72 4 11 82 2 1

73 93 1 4 1 1

74 4 86 5 4 1

75 92 1 4 2 1

76 2 1 95 1 1

77 96 1 1 1 1

78 95 1 2 1 1

79 6 1 87 5 1

80 3 86 2 8 1

81 94 1 2 2 1

82 1 2 1 95 1

83 1 3 1 94 1

84 2 93 1 3 1

85 1 1 1 96 1

86 1 94 3 1 1

87 87 6 1 5 1

88 6 2 88 3 1

89 93 1 3 2 1

90 95 1 2 1 1

91 95 1 1 2 1

92 3 91 2 3 1

93 5 2 3 89 1

94 25 5 3 66 1

95 3 89 2 5 1

96 3 2 2 92 1

97 1 2 1 95 1

98 1 2 1 95 1

99 4 2 88 5 1

100 3 3 2 91 1

101 3 1 92 3 1

102 91 2 2 4 1

103 2 2 1 94 1

104 4 1 92 2 1

105 4 2 1 92 1

106 2 3 69 25 1

107 1 2 3 93 1

108 6 2 90 1 1

109 1 90 1 7 1

110 86 2 3 8 1

111 1 2 1 95 1

112 1 1 1 96 1

113 2 93 3 1 1

114 92 3 1 3 1

115 92 3 2 2 1

116 2 91 2 4 1

117 3 1 1 94 1

118 8 85 2 4 1

119 93 3 2 1 1

120 2 92 3 2 1

121 94 1 3 1 1

122 6 2 88 3 1

123 88 1 5 5 1

124 6 1 88 4 1

125 7 3 2 87 1

126 2 1 2 94 1

127 4 2 92 1 1

128 94 1 2 2 1

129 95 1 2 1 1

130 12 78 3 6 1

131 34 51 4 9 2

132 3 3 1 92 1

133 5 5 2 87 1

134 4 40 2 53 1

135 2 92 2 3 1

136 1 2 1 95 1

137 2 3 1 93 1

138 1 2 1 95 1

139 2 3 1 92 2

140 3 18 75 3 1

141 92 1 3 3 1

142 3 5 2 88 2

143 93 1 2 3 1

144 3 3 92 1 1

145 92 2 2 3 1

146 5 2 91 1 1

147 3 88 3 5 1

148 90 4 2 3 1

149 3 3 91 2 1

150 4 3 2 89 2

151 2 2 1 94 1

152 2 1 2 94 1

153 2 1 4 90 3

154 2 2 94 1 1

155 95 1 1 2 1

156 96 1 1 1 1

157 94 1 1 3 1

158 3 90 3 3 1

159 89 5 2 3 1

160 3 87 5 3 2

161 4 2 3 90 1

162 2 90 4 3 1

163 1 6 2 90 1

164 1 1 1 96 1

165 1 2 1 95 1

166 1 6 1 91 1

167 1 4 4 89 2

168 4 3 88 4 1

169 8 4 1 86 1

170 86 1 6 5 2

171 5 2 88 4 1

PERCON consensus matrix of alpha satellite M1 monomer

# A C G T -

1 86 0 0 14 0

2 94 3 3 0 0

3 0 0 3 97 0

4 3 92 3 0 2

5 0 0 0 100 0

6 5 3 86 5 1

7 5 81 5 8 1

8 97 0 0 3 0

9 95 0 5 0 0

10 14 3 81 0 2

11 5 3 5 86 1

12 5 0 92 3 0

13 3 5 89 3 0

14 97 0 0 3 0

15 5 8 0 86 1

16 100 0 0 0 0

17 3 3 2 92 0

18 2 3 0 95 0

19 3 3 2 92 0

20 0 5 86 8 1

21 5 0 84 11 0

22 84 0 11 5 0

23 11 0 81 5 3

24 11 68 5 16 0

25 19 41 32 8 0

26 5 81 3 11 0

27 3 8 0 89 0

28 0 0 0 100 0

29 0 0 5 95 0

30 5 3 84 5 3

31 68 8 3 19 2

32 3 5 92 0 0

33 8 8 84 0 0

34 7 85 0 8 0

35 8 87 0 5 0

36 3 8 2 87 0

37 90 3 2 5 0

38 3 15 0 82 0

39 10 3 41 46 0

40 0 3 90 5 2

41 0 0 0 100 0

42 5 0 85 10 0

43 13 3 84 0 0

44 92 0 5 3 0

45 100 0 0 0 0

46 97 0 0 3 0

47 90 3 3 0 4

48 3 3 91 3 0

49 8 5 85 0 2

50 100 0 0 0 0

51 92 3 0 5 0

52 87 0 5 5 3

53 0 8 0 92 0

54 97 0 3 0 0

55 2 3 0 95 0

56 2 95 0 3 0

57 5 3 0 92 0

58 0 0 8 92 0

59 0 100 0 0 0

60 90 5 0 3 2

61 7 85 0 8 0

62 87 0 5 8 0

63 3 0 0 97 0

64 97 0 3 0 0

65 97 0 3 0 0

66 100 0 0 0 0

67 97 0 0 3 0

68 87 3 3 5 2

69 0 95 0 5 0

70 8 5 0 87 0

71 92 0 5 3 0

72 3 72 22 3 0

73 92 0 3 5 0

74 3 90 4 3 0

75 92 0 5 3 0

76 3 0 94 3 0

77 97 0 3 0 0

78 92 3 2 3 0

79 18 10 69 3 0

80 8 77 7 8 0

81 85 3 3 8 1

82 8 5 0 85 2

83 3 0 0 95 2

84 0 87 3 8 2

85 3 5 0 92 0

86 3 0 97 0 0

87 92 0 3 5 0

88 5 3 92 0 0

89 100 0 0 0 0

90 90 3 2 5 0

91 95 5 0 0 0

92 0 90 5 5 0

93 2 3 0 95 0

94 5 0 3 92 0

95 0 97 0 3 0

96 5 5 0 90 0

97 0 3 0 97 0

98 3 5 0 90 2

99 5 5 82 8 0

100 0 5 0 95 0

101 0 3 94 3 0

102 79 5 8 8 0

103 0 5 0 95 0

104 13 0 87 0 0

105 0 5 3 92 0

106 10 0 87 3 0

107 3 3 2 92 0

108 8 5 84 3 0

109 3 90 4 3 0

110 90 0 2 8 0

111 0 3 2 95 0

112 2 3 0 95 0

113 5 92 3 0 0

114 92 5 0 3 0

115 21 10 0 69 0

116 5 85 2 8 0

117 3 10 0 87 0

118 2 95 0 3 0

119 97 3 0 0 0

120 0 87 5 8 0

121 87 3 2 8 0

122 0 3 87 10 0

123 85 0 5 8 2

124 10 0 82 5 3

125 0 3 2 95 0

126 0 3 0 97 0

127 8 10 79 0 3

128 100 0 0 0 0

129 100 0 0 0 0

130 10 77 5 8 0

131 10 67 3 10 10

132 2 8 0 90 0

133 26 0 0 74 0

134 0 0 0 100 0

135 5 90 2 3 0

136 0 5 0 95 0

137 0 8 0 92 0

138 5 5 0 90 0

139 0 3 0 90 7

140 0 18 77 5 0

141 87 3 5 3 2

142 7 3 0 90 0

143 5 3 5 87 0

144 0 5 95 0 0

145 84 3 0 13 0

146 0 0 97 3 0

147 2 95 0 3 0

148 100 0 0 0 0

149 3 5 92 0 0

150 0 3 0 87 10

151 0 0 0 100 0

152 0 0 0 100 0

153 5 0 18 77 0

154 0 3 97 0 0

155 90 3 2 5 0

156 90 0 5 5 0

157 77 5 5 13 0

158 10 82 0 5 3

159 69 8 0 21 2

160 8 72 15 5 0

161 0 5 3 92 0

162 5 82 13 0 0

163 0 3 2 95 0

164 0 0 0 100 0

165 0 5 3 92 0

166 0 0 0 100 0

167 3 8 0 87 2

168 8 16 71 3 2

169 3 5 0 92 0

170 92 0 8 0 0

171 0 5 92 3 0

PERCON consensus matrix of Xm random monomer

# A C G T -

1 25 25 25 25 0

2 25 25 25 25 0

3 25 25 25 25 0

4 25 25 25 25 0

5 25 25 25 25 0

6 25 25 25 25 0

7 25 25 25 25 0

8 25 25 25 25 0

9 25 25 25 25 0

10 25 25 25 25 0

11 25 25 25 25 0

12 25 25 25 25 0

13 25 25 25 25 0

14 25 25 25 25 0

15 25 25 25 25 0

16 25 25 25 25 0

17 25 25 25 25 0

18 25 25 25 25 0

19 25 25 25 25 0

20 25 25 25 25 0

21 25 25 25 25 0

22 25 25 25 25 0

23 25 25 25 25 0

24 25 25 25 25 0

25 25 25 25 25 0

26 25 25 25 25 0

27 25 25 25 25 0

28 25 25 25 25 0

29 25 25 25 25 0

30 25 25 25 25 0

31 25 25 25 25 0

32 25 25 25 25 0

33 25 25 25 25 0

34 25 25 25 25 0

35 25 25 25 25 0

36 25 25 25 25 0

37 25 25 25 25 0

38 25 25 25 25 0

39 25 25 25 25 0

40 25 25 25 25 0

41 25 25 25 25 0

42 25 25 25 25 0

43 25 25 25 25 0

44 25 25 25 25 0

45 25 25 25 25 0

46 25 25 25 25 0

47 25 25 25 25 0

48 25 25 25 25 0

49 25 25 25 25 0

50 25 25 25 25 0

51 25 25 25 25 0

52 25 25 25 25 0

53 25 25 25 25 0

54 25 25 25 25 0

55 25 25 25 25 0

56 25 25 25 25 0

57 25 25 25 25 0

58 25 25 25 25 0

59 25 25 25 25 0

60 25 25 25 25 0

61 25 25 25 25 0

62 25 25 25 25 0

63 25 25 25 25 0

64 25 25 25 25 0

65 25 25 25 25 0

66 25 25 25 25 0

67 25 25 25 25 0

68 25 25 25 25 0

69 25 25 25 25 0

70 25 25 25 25 0

71 25 25 25 25 0

72 25 25 25 25 0

73 25 25 25 25 0

74 25 25 25 25 0

75 25 25 25 25 0

76 25 25 25 25 0

77 25 25 25 25 0

78 25 25 25 25 0

79 25 25 25 25 0

80 25 25 25 25 0

81 25 25 25 25 0

82 25 25 25 25 0

83 25 25 25 25 0

84 25 25 25 25 0

85 25 25 25 25 0

86 25 25 25 25 0

87 25 25 25 25 0

88 25 25 25 25 0

89 25 25 25 25 0

90 25 25 25 25 0

91 25 25 25 25 0

92 25 25 25 25 0

93 25 25 25 25 0

94 25 25 25 25 0

95 25 25 25 25 0

96 25 25 25 25 0

97 25 25 25 25 0

98 25 25 25 25 0

99 25 25 25 25 0

100 25 25 25 25 0

101 25 25 25 25 0

102 25 25 25 25 0

103 25 25 25 25 0

104 25 25 25 25 0

105 25 25 25 25 0

106 25 25 25 25 0

107 25 25 25 25 0

108 25 25 25 25 0

109 25 25 25 25 0

110 25 25 25 25 0

111 25 25 25 25 0

112 25 25 25 25 0

113 25 25 25 25 0

114 25 25 25 25 0

115 25 25 25 25 0

116 25 25 25 25 0

117 25 25 25 25 0

118 25 25 25 25 0

119 25 25 25 25 0

120 25 25 25 25 0

121 25 25 25 25 0

122 25 25 25 25 0

123 25 25 25 25 0

124 25 25 25 25 0

125 25 25 25 25 0

126 25 25 25 25 0

127 25 25 25 25 0

128 25 25 25 25 0

129 25 25 25 25 0

130 25 25 25 25 0

131 25 25 25 25 0

132 25 25 25 25 0

133 25 25 25 25 0

134 25 25 25 25 0

135 25 25 25 25 0

136 25 25 25 25 0

137 25 25 25 25 0

138 25 25 25 25 0

139 25 25 25 25 0

140 25 25 25 25 0

141 25 25 25 25 0

142 25 25 25 25 0

143 25 25 25 25 0

144 25 25 25 25 0

145 25 25 25 25 0

146 25 25 25 25 0

147 25 25 25 25 0

148 25 25 25 25 0

149 25 25 25 25 0

150 25 25 25 25 0

151 25 25 25 25 0

152 25 25 25 25 0

153 25 25 25 25 0

154 25 25 25 25 0

155 25 25 25 25 0

156 25 25 25 25 0

157 25 25 25 25 0

158 25 25 25 25 0

159 25 25 25 25 0

160 25 25 25 25 0

161 25 25 25 25 0

162 25 25 25 25 0

163 25 25 25 25 0

164 25 25 25 25 0

165 25 25 25 25 0

166 25 25 25 25 0

167 25 25 25 25 0

168 25 25 25 25 0

169 25 25 25 25 0

170 25 25 25 25 0

171 25 25 25 25 0

C. Aligned consensus sequences of alpha satellite monomer classes used in this work. Where the sequences published in [4] and deposited in GenBank were modified, the position in GenBank sequence is shown first and the position in an updated sequence is shown after an arrow.

>cons_A_type_AJ131207|modified|12-🡪G|44A🡪deleted

AATCTGCAAGTGGANATTTGGAGCGCTTTGAGGCCTATGGTGGAAAAGGAAATATCTTCACATAAAAACTAGACAGAAGCATTCTCAGAAACTTCTTTGTGATGTGTGCATTCAACTCACAGAGTTGAACCTTTCTTTTGATAGAGCAGTTTNGAAACACTCTTTTTGTAG

>cons_B_type_AJ131208

AATCTGCAAGTGGATATTTGGACCNCTTTGAGGCCTTCGTTGGAAACGGGAATATCTTCACATAAAANCTAGACAGAAGCATTCTCAGAAACTTCTTTGTGATGTNTGCATTCAACTCACAGAGTTGAACNTTCCTTTTGATAGAGCAGNTTTGAAACACTCTTTTTGTAG

>cons_J1_AJ130753

AATTTGCAAGTGGAGATTTCAAGCGCTTTGAGGTCAATGGTAGAAAAGGAAATATCTTCGTATAAAAACTAGACAGAATCATTCTCAGAAACTNCTTTGTGATGTGTGCGTTCAACTCACAGAGTTTAACCTTTCTTTTCATAGAGCAGTTNGGAAACACTCTGTTTGTAA

>cons_J2_AJ130754|modified|19C🡪T|20A🡪G|63N🡪T|64N🡪A|65T🡪A|66G🡪T|67A🡪G|79A🡪G|80C🡪A|88T🡪G|89A🡪T|101T🡪G|102A🡪T|108T🡪G|109C🡪T|130N🡪C|138A🡪T|139TA|141C🡪A|142T🡪C|149A🡪G|150T🡪A|169G🡪T|170A🡪G

AGTCTGCAAGTGGATATTTGGACCTCTTTGAGGCCTTCGTTGGAAACGGGA-TTTCTTCATATAATG-CTAGACAGAAGAATTCTCAGTAACTTCTTTGTGTTGTGTGTATTCAACTCACAGAGTTGAACCTTCCTTTAGACAGAGCAGATTTGAAACACTCTTTTTGTGG

>cons_D1_AJ130751

AATCTGCAAGTGGATATTTGGATAGCTTTGAGGATTTCGTTGGAAACGGGAATATCNTCATATAAAATCTAGACAGAAGCATTCTCAGAAACTTCTTTGTGATGTNTGCATTCAAGTCACAGAGTTGAACATTCCCTTTCATAGAGCAGGTTTGAAACACTCTTTTTGTAG

>cons_D2_AJ130752

TATCTGGAAGTGGACATTTGGAGCGCTTTGAGGCCTATGGTGAAAAAGGAAATATCTTCCCATAAAAACTAGACAGAAGCATTCTCAGAAACTTNTTTGTGATGTGTGTACTCAACTAACAGAGTTGAACCTTTCTTTTGATAGAGCAGTTTTGAAACACTCTTTTTGTNG

>cons_W1_AJ130758

AATCTGCAAGTGGATATTTGGACCTCTCTGAGGATTTCGTTGGAAACGGGATAAACTTCACATAA---CTAAACAGAAGCATTCTCAGAAACTTCTTTGTGATGTTTGCATTCAACTCACAGAGTTGAACCTTCCTTT-GATAGTTCAGGTTTGAAACACTCTTTTTGTAG

>cons_W2_AJ130759

AATCTGCAAGTGGATATTTGGACCACTTTGTGGCCTTCGTTCGAAACGGGTATATCTTCACATCAAACCTAGACAGAAGCATTCTCAGAANGTTTTCTGTGATGACTGCATTCAACTCACAGAGTTGAACAATCCTNTTGATGGAGCAGTTTTGAAACTCTCTTTCTTTGG

>cons_W3_AJ130760

AATCTGCAAGTGGATATGTGGACCTCTTTGAAGATTTCGTTGGAAACGGGATCATCTTCACATAAAAACTAAACAGAAGCATTCTCAGAAACTNCTTTGTGATGTTTGTGTTCAACTCCCAGAGTTGAACTTTCCTTTTGANAGAGCAGCTNTGAAACACTCTTTTTCTAG

>cons_W4_AJ130761

AATCTGCAAGTGGACATTTGGAGGGCTTTGAGGCCTGTGGTGGAAAAGGAAATATCTTCACATAAAAACTAGATAGAAGCATTCTCAGAAACTACTTTGTGATGATTGCATTCAACTCACAGAGTTGAACATTCCTTTTGATAGAGCAGTTTGGAAACACTCTTTTTGTAG

>cons_W5_AJ130762

AATCTGCAAGTGGAGATTTGGACCGCTTTGAGGCCTANGGTAGTAAAGGAAATAACTTCATATAAAAACTAGACAGAAGCATTCTCAGAAAATTCTTTGTGATGATTGAGTTTAACTCACAGAGCTGAACATTCCTTTNGATGGAGCAGTTTCNAAACACACTTTTTGTAG

>cons_R1_AJ130756

AATCTGCANGTGGATATTTGGAGCNCTTTGAGGCCTTCNTTGGAAACGGGAATATCTTCACATAAAAACTAGACAGAAGCATTCTCAGAAACTTCTTTGTGATGTGTGCATTCAACTCACAGAGTTGAACCTTCCTTTTGATAGAGCAGTTTTGAAACACTCTTTTTGTAG

>cons_R2_AJ130757

AATCTGCAAGTGGACATTTGGAGCGCTTTGAGGCCTATGGTGGAAAAGGAAATATCTTCACATAAAAACTAGACAGAAGCATTCTCAGAAACTTCTTTGTGATGTGTGCATTCAACTCACAGAGTTGAACCTTTCTTTTGATAGAGCAGTTTTGAAACACTCTTTTTGTAG

>cons_M1_AJ130755

AATCTGCAAGTGGATATTTGGAGCNCTTTGAGGCCTATNGTGGAAAAGGAAATATCTTCACATAAAAACTACACAGAAGCATTCTGAGAAACTTCTTTGTGATGTGTGCATTCATCTCACAGAGTTGAACCTTTCTTTTGATTGAGCAGTTTTGAAACACTCTTTTTGTAG

**Figure S2**


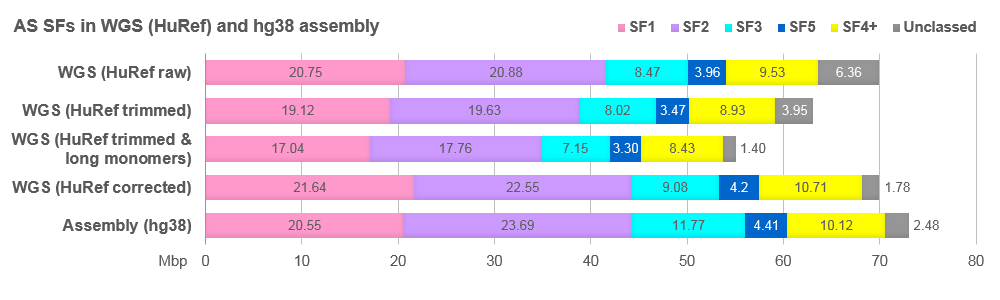


**Table S2. R1 density gradient in SF5 arrays of hg38 assembly.**

| **SF** | **Location** | **Position in hg38** | **Contig** | **Size (bp)*** | **R1%** | **R2 %** | **A-box %** | **B-box %** |
| --- | --- | --- | --- | --- | --- | --- | --- | --- |
| SF5 | 16p11.1 | [chr16:36001814-36022913](http://genome.ucsc.edu/cgi-bin/hgTracks?db=hg38&position=chr16:36001814-36022913) | AC109490.3 | 21,099 | 0 | 94 | 94 | 0 |
| SF5 | 7p11.2 | [chr7:57939175-57953728](http://genome.ucsc.edu/cgi-bin/hgTracks?db=hg38&position=chr7:57939175-57953728) | AC138789.1 | 10,294 | 0 | 94 | 90 | 0 |
| SF5 | 11p11.12 | [chr11:48806070-48814307](http://genome.ucsc.edu/cgi-bin/hgTracks?db=hg38&position=chr11:48806070-48814307) | AC127495.2 | 8,237 | 0 | 91 | 94 | 0 |
| SF5 | 20q11.1 | [chr20:30088752-30140826](http://genome.ucsc.edu/cgi-bin/hgTracks?db=hg38&position=chr20:30088752-30140826) | FP565326.9 | 51,870 | 0 | 83 | 98 | 0 |
| SF5 | 7q11.21 | [chr7:62536194-62564614](http://genome.ucsc.edu/cgi-bin/hgTracks?db=hg38&position=chr7:62536194-62564614) | AC019063.4 | 24,011 | 0 | 83 | 86 | 2 |
| SF5 | 20q11.1 | [chr20:29908640-30038347](http://genome.ucsc.edu/cgi-bin/hgTracks?db=hg38&position=chr20:29908640-30038347) | ABBA01018540.1 GJ212117.1 | 128,442 | 0 | 80 | 97 | 0 |
| SF5 | 12q11 | [chr12:37632794-37639361](http://genome.ucsc.edu/cgi-bin/hgTracks?db=hg38&position=chr12:37632794-37639361) | AC119042.9 | 6,567 | 0 | 75 | 93 | 0 |
| SF5 | 10p11.1 | [chr10:39432620-39442102](http://genome.ucsc.edu/cgi-bin/hgTracks?db=hg38&position=chr10:39432620-39442102) | ABBA01020709.1 | 6,981 | 0 | 70 | 92 | 0 |
| SF5 | 6q11.1 | [chr6:61326977-61336104](http://genome.ucsc.edu/cgi-bin/hgTracks?db=hg38&position=chr6:61326977-61336104) | AMYH02013791.1** | 9,127 | 2 | 78 | 94 | 2 |
| SF5 | 6q11.1 | [chr6:61428794-61437937](http://genome.ucsc.edu/cgi-bin/hgTracks?db=hg38&position=chr6:61428794-61437937) | FP325349.3** | 9,143 | 2 | 78 | 94 | 2 |
| SF5 | 16p11.1 | [chr16:36079689-36090000](http://genome.ucsc.edu/cgi-bin/hgTracks?db=hg38&position=chr16:36079689-36090000) | FP325312.10 | 10,311 | 3 | 93 | 86 | 0 |
| SF5 | Xq11.1 | [chrX:62611837-62642074](http://genome.ucsc.edu/cgi-bin/hgTracks?db=hg38&position=chrX:62611837-62642074) | BX544875.1 | 30,237 | 3 | 90 | 85 | 3 |
| SF5 | 5p11 | [chr5:46569260-46796649](http://genome.ucsc.edu/cgi-bin/hgTracks?db=hg38&position=chr5:46569260-46796649) | GJ211883.1 | 227,293 | 13 | 85 | 70 | 11 |
| SF5 | 8p11.1 | [chr8:43904622-43936350](http://genome.ucsc.edu/cgi-bin/hgTracks?db=hg38&position=chr8:43904622-43936350) | AC127507.4 | 29,276 | 17 | 74 | 75 | 16 |
| SF5 | 5p11 | [chr5:46485995-46568985](http://genome.ucsc.edu/cgi-bin/hgTracks?db=hg38&position=chr5:46485995-46568985) | GJ211882.1 | 82,036 | 24 | 72 | 61 | 12 |
| SF5 | Xq11.1 | [chrX:62593383-62608885](http://genome.ucsc.edu/cgi-bin/hgTracks?db=hg38&position=chrX:62593383-62608885) | BX544875.1 | 15,204 | 26 | 62 | 59 | 30 |
| SF5 | 12q11 | [chr12:37596084-37616677](http://genome.ucsc.edu/cgi-bin/hgTracks?db=hg38&position=chr12:37596084-37616677) | AC129805.7 | 20,593 | 27 | 67 | 63 | 25 |
| SF5 | 7p11.2 | [chr7:58003416-58029302](http://genome.ucsc.edu/cgi-bin/hgTracks?db=hg38&position=chr7:58003416-58029302) | AC110072.3 | 25,580 | 28 | 61 | 61 | 28 |
| SF5 | Xq11.1 | [chrX:62506814-62549062](http://genome.ucsc.edu/cgi-bin/hgTracks?db=hg38&position=chrX:62506814-62549062) | BX537339.3 BX544875.1 | 36,226 | 30 | 65 | 65 | 24 |
| SF5 | 17q11.1 | [chr17:26641687-26710610](http://genome.ucsc.edu/cgi-bin/hgTracks?db=hg38&position=chr17:26641687-26710610) | ABBA01004491.1 ABBA01004492.1 ABBA01004493.1 | 57,880 | 30 | 63 | 62 | 28 |
| SF5 | 5p11 | [chr5:47073733-47079727](http://genome.ucsc.edu/cgi-bin/hgTracks?db=hg38&position=chr5:47073733-47079727) | ABBA01000240.1 ABBA01000241.1 | 5,877 | 31 | 64 | 57 | 17 |
| SF5 | 5p11 | [chr5:46796922-47061211](http://genome.ucsc.edu/cgi-bin/hgTracks?db=hg38&position=chr5:46796922-47061211) | GJ211884.1 | 263,918 | 31 | 61 | 59 | 19 |
| SF5 | 1q11 | [chr1:124941274-125063297](http://genome.ucsc.edu/cgi-bin/hgTracks?db=hg38&position=chr1:124941274-125063297) | ABBA01017604.1 ABBA01017605.1 ABBA01017606.1 ABBA01017607.1 ABBA01017608.1 | 101,960 | 32 | 65 | 63 | 27 |
| SF5 | 10p11.1 | [chr10:39497337-39548228](http://genome.ucsc.edu/cgi-bin/hgTracks?db=hg38&position=chr10:39497337-39548228) | ABBA01020707.1 | 44,883 | 32 | 61 | 61 | 28 |
| SF5 | 20p11.1 | [chr20:26278148-26336297](http://genome.ucsc.edu/cgi-bin/hgTracks?db=hg38&position=chr20:26278148-26336297) | AL358116.7 | 53,407 | 32 | 53 | 54 | 35 |
| SF5 | 11p11.12 | [chr11:50799481-50813412](http://genome.ucsc.edu/cgi-bin/hgTracks?db=hg38&position=chr11:50799481-50813412) | AC126398.5 | 13,931 | 32 | 51 | 56 | 34 |
| SF5 | 5p11 | [chr5:46335504-46423806](http://genome.ucsc.edu/cgi-bin/hgTracks?db=hg38&position=chr5:46335504-46423806) | AC125790.2 AC125787.2 AUXG01000271.1 | 71,552 | 33 | 59 | 59 | 33 |
| SF5 | 5q11.1 | [chr5:49721570-50059730](http://genome.ucsc.edu/cgi-bin/hgTracks?db=hg38&position=chr5:49721570-50059730) | GJ211906.2 | 338,160 | 33 | 56 | 54 | 43 |
| SF5 | 12q11 | [chr12:37275866-37317872](http://genome.ucsc.edu/cgi-bin/hgTracks?db=hg38&position=chr12:37275866-37317872) | AEKP01211346.1 ABBA01049497.1 | 42,006 | 34 | 63 | 64 | 18 |
| SF5 | 5p11 | [chr5:47107090-47153262](http://genome.ucsc.edu/cgi-bin/hgTracks?db=hg38&position=chr5:47107090-47153262) | GJ211886.1 | 46,172 | 34 | 62 | 58 | 20 |
| SF5 | 19p11 | [chr19:24420597-24448980](http://genome.ucsc.edu/cgi-bin/hgTracks?db=hg38&position=chr19:24420597-24448980) | AC073541.4 | 28,383 | 34 | 59 | 53 | 44 |
| SF5 | 3q11.1 | [chr3:93731526-93759547](http://genome.ucsc.edu/cgi-bin/hgTracks?db=hg38&position=chr3:93731526-93759547) | ABBA01026974.1 | 24,891 | 35 | 60 | 64 | 29 |
| SF5 | 11q11 | [chr11:55247791-55260068](http://genome.ucsc.edu/cgi-bin/hgTracks?db=hg38&position=chr11:55247791-55260068) | AP005597.2 | 12,277 | 35 | 53 | 48 | 36 |
| SF5 | 22q11.1 | [chr22:15159872-15178976](http://genome.ucsc.edu/cgi-bin/hgTracks?db=hg38&position=chr22:15159872-15178976) | FP885535.8 | 19,104 | 35 | 50 | 53 | 40 |
| SF5 | 5q11.1 | [chr5:49667627-49721122](http://genome.ucsc.edu/cgi-bin/hgTracks?db=hg38&position=chr5:49667627-49721122) | GJ211904.2 | 53,495 | 37 | 57 | 53 | 44 |
| SF5 | 3p11.1 | [chr3:90398578-90483398](http://genome.ucsc.edu/cgi-bin/hgTracks?db=hg38&position=chr3:90398578-90483398) | AC144764.4 ABBA01004652.1 | 72,697 | 37 | 55 | 56 | 33 |
| SF5 | 14q11.2 | [chr14:18223609-18248178](http://genome.ucsc.edu/cgi-bin/hgTracks?db=hg38&position=chr14:18223609-18248178) | CR383659.1 | 24,569 | 38 | 45 | 46 | 46 |
| SF5 | 7q11.1 | [chr7:62029827-62040626](http://genome.ucsc.edu/cgi-bin/hgTracks?db=hg38&position=chr7:62029827-62040626) | AC123577.3 | 10,799 | 41 | 56 | 53 | 38 |
| SF5 | 7q11.1 | [chr7:60878274-60888901](http://genome.ucsc.edu/cgi-bin/hgTracks?db=hg38&position=chr7:60878274-60888901) | ABBA01017246.1 | 10,627 | 41 | 55 | 54 | 37 |
| SF5 | 21q11.1 | [chr21:12967006-12979569](http://genome.ucsc.edu/cgi-bin/hgTracks?db=hg38&position=chr21:12967006-12979569) | AP001464.1 | 12,563 | 42 | 51 | 49 | 41 |
| SF5 | 20q11.21 | [chr20:30965479-31001415](http://genome.ucsc.edu/cgi-bin/hgTracks?db=hg38&position=chr20:30965479-31001415) | AC104301.2 | 21,883 | 42 | 50 | 52 | 35 |
| SF5 | 7q11.1 | [chr7:61104950-61265720](http://genome.ucsc.edu/cgi-bin/hgTracks?db=hg38&position=chr7:61104950-61265720) | AC017075.8 | 159,316 | 43 | 57 | 57 | 41 |
| SF5 | 7q11.1 | [chr7:61377883-61527944](http://genome.ucsc.edu/cgi-bin/hgTracks?db=hg38&position=chr7:61377883-61527944) | GJ212194.1 | 150,061 | 43 | 56 | 55 | 42 |
| SF5 | 2q11.1 | [chr2:94549885-94559437](http://genome.ucsc.edu/cgi-bin/hgTracks?db=hg38&position=chr2:94549885-94559437) | ABBA01003589.1 | 9,552 | 43 | 53 | 53 | 44 |
| SF5 | 20q11.1 | [chr20:30145193-30167104](http://genome.ucsc.edu/cgi-bin/hgTracks?db=hg38&position=chr20:30145193-30167104) | FP565326.9 | 20,341 | 44 | 48 | 51 | 42 |
| SF5 | 22q11.1 | [chr22:16029129-16043988](http://genome.ucsc.edu/cgi-bin/hgTracks?db=hg38&position=chr22:16029129-16043988) | AC145543.3 | 14,859 | 44 | 45 | 48 | 42 |
| SF5 | 9q11 | [chr9:43191256-43201651](http://genome.ucsc.edu/cgi-bin/hgTracks?db=hg38&position=chr9:43191256-43201651) | FP325317.7 | 10,395 | 45 | 52 | 47 | 49 |
| SF5 | 20q11.1 | [chr20:29691107-29722974](http://genome.ucsc.edu/cgi-bin/hgTracks?db=hg38&position=chr20:29691107-29722974) | ABBA01031671.1 ABBA01031672.1 AC011850.12 | 31,472 | 46 | 49 | 51 | 42 |
| SF5 | 2q11.1 | [chr2:94513503-94541352](http://genome.ucsc.edu/cgi-bin/hgTracks?db=hg38&position=chr2:94513503-94541352) | FO624991.2 ABBA01003589.1 | 27,849 | 46 | 44 | 46 | 47 |
| SF5 | 11p11.11 | [chr11:51078444-51090240](http://genome.ucsc.edu/cgi-bin/hgTracks?db=hg38&position=chr11:51078444-51090240) | GJ211938.1 | 11,796 | 47 | 37 | 46 | 34 |
| SF5 | 22q11.1 | [chr22:15991000-16003447](http://genome.ucsc.edu/cgi-bin/hgTracks?db=hg38&position=chr22:15991000-16003447) | AC145543.3 | 12,447 | 48 | 46 | 48 | 48 |
| SF5 | 11p11.12 | [chr11:48841601-48879449](http://genome.ucsc.edu/cgi-bin/hgTracks?db=hg38&position=chr11:48841601-48879449) | AC027369.8 | 28,940 | 48 | 43 | 43 | 43 |
| SF5 | 15q11.1 | [chr15:19790550-19800262](http://genome.ucsc.edu/cgi-bin/hgTracks?db=hg38&position=chr15:19790550-19800262) | ABBA01004580.1 AC145435.3 | 9,712 | 49 | 47 | 50 | 42 |
| SF5 | 9q13 | [chr9:64963094-64982848](http://genome.ucsc.edu/cgi-bin/hgTracks?db=hg38&position=chr9:64963094-64982848) | AC125634.1 | 19,754 | 50 | 40 | 45 | 48 |
| SF5 | 9p11.2 | [chr9:40829846-40852330](http://genome.ucsc.edu/cgi-bin/hgTracks?db=hg38&position=chr9:40829846-40852330) | AL353626.5 | 22,484 | 50 | 38 | 44 | 48 |
| SF5 | 14q11.2 | [chr14:18258254-18267119](http://genome.ucsc.edu/cgi-bin/hgTracks?db=hg38&position=chr14:18258254-18267119) | CR383659.1 | 8,865 | 51 | 46 | 44 | 51 |
| SF5 | 22q11.1 | [chr22:15189051-15197911](http://genome.ucsc.edu/cgi-bin/hgTracks?db=hg38&position=chr22:15189051-15197911) | FP885535.8 | 8,860 | 52 | 46 | 44 | 52 |
| SF5 | 13p11.2 | [chr13:16228723-16249215](http://genome.ucsc.edu/cgi-bin/hgTracks?db=hg38&position=chr13:16228723-16249215) | GJ211965.2 | 20,492 | 55 | 44 | 41 | 55 |
| SF5 | 5p11 | [chr5:47073782-47106837](http://genome.ucsc.edu/cgi-bin/hgTracks?db=hg38&position=chr5:47073782-47106837) | ABBA01000240.1 ABBA01000241.1 ABBA01000242.1 | 28,464 | 59 | 34 | 35 | 52 |
| SF5 | 11p11.12 | [chr11:48861998-48877085](http://genome.ucsc.edu/cgi-bin/hgTracks?db=hg38&position=chr11:48861998-48877085) | AC027369.8 | 14,183 | 60 | 31 | 36 | 49 |
| SF5 | 19q11 | [chr19:27251855-27280389](http://genome.ucsc.edu/cgi-bin/hgTracks?db=hg38&position=chr19:27251855-27280389) | AC010517.3 | 24,900 | 61 | 34 | 36 | 55 |
| SF5 | 11p11.12 | [chr11:48909760-48942428](http://genome.ucsc.edu/cgi-bin/hgTracks?db=hg38&position=chr11:48909760-48942428) | AC027369.8 | 29,212 | 63 | 29 | 34 | 52 |

* Size has been corrected to exclude L1-repeats and gaps.

** These contigs are partially segment duplications of each other.

­­­**Table S3. Presence of SF4+ HORs in AS reference models and genomic contigs.**

|  |  | **Reference model** | | | | | **Genomic representative** | | | |
| --- | --- | --- | --- | --- | --- | --- | --- | --- | --- | --- |
| **#** | **SF** | **Chromosome** | **Name** | **Size (bp)** | **Main HOR**  **size (kb)** | **Id (%)^1^** | **Name** | **Location (size, bp)** | **Id to reference model (%)^1^** | **Comment^2^** |
| 1 | SF4+ | chr13,14,21,22**^3^** | GJ211955.2 | 22,537 | 5.6 | 97 | AC068673.2 | chr3 (134,319) | 97 | 81-86 kb, incomplete HOR copy |
|  |  |  |  |  |  |  | AC242390.1 | chr21 (39,976) | 96 | 1-5 kb, incomplete HOR copy |
|  |  |  |  |  |  |  | AF254982.4 | chr21:10,606,141-10,814,560 (211,345) | 96 | 20-25 kb, incomplete HOR copy |
| 2 | SF4+ | chr13,14,21,22**^3^** | GJ211961.2 | 88,022 | 3.9 | 97 | AADC01164618.1 | chrUn (92,432) | 97 | 22-40 kb, HOR 3.9 kb, id=99.2% |
|  |  |  |  |  |  |  | FP236243.10 | chr21:7,997,247-8,001,000 (3,754) | 97 | 55-134 kb, HOR 3.9 kb, id=98.3% |
|  |  |  |  |  |  |  | CT476838.7 | chr21 (129,466) | 97 | 1-69kb, HOR 3.9 kb, overlaps FP236243 |
| 3 | SF4+ | chr13,14,21,22**^3^** | GJ211962.2 | 54,133 | 3.4**^5^** | 97 | AC022192.3 | HTGS, chrUn (156,897) | 97 | 124-140 kb,153 kb-end, HOR 3.4 kb, id>99%, overlaps non-AS clone AC138776.2 on chr22 |
| 4 | SF4+ | chr13,14,21,22**^3^** | GJ211963.X**^4^** | 63,535 | 3.4**^5^** | 95 | JSAF02034270.1 | chrUn (25,083) | 98 | HOR 3.4 kb, id=99.9% |
|  |  |  |  |  |  |  | JSAF02012309.1 | chrUn (22,846) | 97 | HOR 3.4 kb, id=99.9% |
|  |  |  |  |  |  |  | JSAF02018645.1 | chrUn (13,430) | 97 | HOR 3.4 kb, id=99.4% |
| 5 | SF4+ | chr13,14,21,22**^3^** | GJ211967.2 | 6,670 | 2.7 | 96 | AC137488.2 | chr22:11,210,922-11,378,056 (167,135) | >99 | Single HOR copy, SD on chr20:29,836,830-29,864,984 |
|  |  |  |  |  |  |  | AC242688.1 | HTGS, chr16 (17,992) | >99 | 1-16kb: HOR 2.7 kb (variant 2.9 kb), id=99% |
| 6 | SF4+ | chr13,14,21,22**^3^** | GJ211968.2 | 3,245 | 1.4 | 95 | AC024039.4 | chr3 (160,502) | 95 | <2 HOR copies |
|  |  |  |  |  |  |  | AC022645.3 | HTGS, chrUn (155,380) | 95 | <2 HOR copies |
|  |  |  |  |  |  |  | AC068673.2 | chr3 (134,319) | 95 | <2 HOR copies |
|  |  |  |  |  |  |  | AC067769.2 | chr16 (161,855) | 95 | <2 HOR copies |
| 7 | SF4+ | chr13,14,21,22**^3^** | GJ211969.2 | 22,561 | 2.9 | 96 | CU638690.5 | chr21:7,199,528-7,327,865 (128,338) | low | Incomplete HOR copy. No continuous high identity. Small high identity regions, overall 92% - 94%. |
|  |  |  |  |  |  |  | JSAF02004742.1 | chrUn (46,248) | 96 | 17-34 kb, HOR 2.9 kb, id~100% |
|  |  |  |  |  |  |  | JSAF02029371.1 | chrUn (19,447) | 97 | 1-8 kb: HOR 2.9 kb, id>99% |
| 8 | SF4+ | chr13,14,21,22**^3^** | GJ211986.2 | 1,198 | -**^6^** | - | AC242688.1 | chr16 (17,992) | 94 | 0.9 kb 94% identical to GJ211986 |
|  |  |  |  |  |  |  | JSAF02036319.1 | chrUn (19,139) | 95 | HOR 2 kb, id=99%**^6^** |
|  |  |  |  |  |  |  | JSAF02034085.1 | chrUn (17,409) | 94 | 1-5 kb HOR 2kb, id=99%, 8 kb-end, variant of the same HOR 2.7 kb long, id=99%**^6^** |
|  |  |  |  |  |  |  | JSAF02032446.1 | chrUn (16,842) | 95 | HOR 2 kb, id=99%**^6^** |
| 9 | SF4+ | chr15 | GJ212036.1 | 415,278 | 4.3**^7^** | >99 | AC087459.4 | chr15 (96,245) | 98 | 26-33 kb, HOR 4.3 kb, id>99% |
|  |  |  |  |  |  |  | AC018668.5 | chr15 (78,595) | 98 | 64 kb-end, HOR 4.3 kb, id>99% |
| 10 | SF4+ | chr15 | GJ212042.1 | 855,957 | 3.4**^7^** | >99 | AC244148.2 | chr9 (39,894) | >99 | 9 kb-end, HOR 3.4 kb, id>99%  this HOR domain may belong to chr9 |
| 11 | SF4+ | chr20 | GJ212105.1 | 80,766 | 1.9 | >99 | ADDF02210401.1 | chrUn (27,457) | >99 | 13 kb-end, HOR 1.9 kb, id>99% |
|  |  |  |  |  |  |  | AADC01167544.1 | chrUn (15,216) | 99 | 2.5 kb-end, HOR 1.9 kb, id>99% |
|  |  |  |  |  |  |  | AL837517.14 | chr20:28,958,247-29,048,331 (90,085) | >99 | 2-55 kb, HOR 1.9 kb, id>99% |
| 12 | SF4+ | chr20 | GJ212107.X**^4^** | 78,875 | 1.1**^8^** | 95 | ADDF02210418.1 | chrUn (11,255) | 92 | HOR-like structure, 6- and 7-mers, id=93% |
|  |  |  |  |  |  |  | JSAF02027206.1 | chrUn (245,402) | 92 | 54-70 kb, HOR 3.8 kb, id>99%**^9^** |
|  |  |  |  |  |  |  |  |  | 96 | 70-85 kb, HOR-like structure, 6-and 7-mers |
|  |  |  |  |  |  |  | AC073483.4 | HTGS, chrUn (147,712) | 97 | 119-128 kb, HOR-like structure, 5- and 6-mers, id=96% |
|  |  |  |  |  |  |  | ABBA01015872.1 | chr20:28,820,664-28,843,401 (22,738) | 97 | <2 HOR copies |

**^1^** “Id” means inter-HOR identity obtained on a dot-matrix map in an alignment of a randomly chosen diagonal region larger or near the HOR size in reference model area with clear regular diagonals. The alignment length from which the id figure is obtained is larger or near the HOR size. “Id to reference model” shows identity in alignment between a genomic contig and a reference model obtained in a similar manner. Sometimes short regions of high identity are flanked by somewhat more diverged sequence, so in few cases the “Id to reference model” shows identity less than 95%.

**^2^** In “Comment,” if the region where the HOR domain is located in a contig is not specified, it means it occupies the whole sequence, and “id” means identity between copies of HOR in the same contig. The latter value refers to “typical” HOR arrays where dot matrix shows good diagonals, not to some less regular regions of “HOR-like structure” which might flank the HOR array.

**^3^** Only one representative member of a group of identical reference models is listed. For complete list see Supplementary Table S1.

**^4^** Corrected versions of these reference models were obtained from K. Miga and used for analysis.

**^5^** Entry #4 in this table refers to one of the HORs that were erroneously assembled. After it was extracted from the corrected version of the reference model, it appeared to be 94% identical to the HOR in entry #3. The length of both HORs is 3.4 kb. So, it is not clear whether they should be considered as one HOR or two slightly different HORs.

**^6^** These contigs have the same 2kb HOR, which has ~1kb region 94% - 95% identical to a part of GJ211986.2. The latter is 1.2kb long and does not have a HOR within. Thus, the HOR is longer than the reference model, which appears to be just one partial and permutated copy of a HOR present in JSAF02 sequences listed in this entry.

**^7^** The two HORs from chromosome 15 have a 1.5kb common region. The identity between the two HORs in this region is 96%.

**^8^** This reference model has areas of HOR-like structure composed of intermingled 6-mer and 7-mer. Id = 95% corresponds to alignment of regions 20,220-22,754 and 23,465-26,000 in the model (alignment length = 2,538 bp). This is the best diagonal in randomly chosen 3 kb x 3 kb square.

**^9^** Somewhat rearranged sequence 99% identical to whole ADDF02210418 is contained within JSAF02027206. The latter also has discontinuous regions of almost 100% identity to contig GL000198 in chromosome 20, of which ABBA01015872 is a part. The 6-mers (schematic structure ABDECG) and 7-mers (ABDDECG) which form the HOR-like structure are the same in these 3 contigs. The 3.8 kb HOR in JSAF02027206 resulted from amplification of somewhat rearranged piece of the same HOR-like structure. It has one intact 6-mer and pieces of several others. The HOR-like structure in AC073483 has the same 6-mer and a 5-mer, which may be an equivalent of ABDEG.

**Table S3.** ­­­**Presence of SF4+ HORs in AS reference models and genomic contigs.**

It is not known to what extent AS reference models are representative of the true genomic arrangement of respective sequences. Among others, we considered a possibility that some shorter reference models could be created artificially and represented not a tandem HOR region or regions, but a system of SDs one or several of which had an inner tandem duplication. Such tandem duplication would qualify a sequence as a HOR and could seed a reference model which brings together the dispersed copies of the SD and arranges their parts into an array. Therefore we wanted to make sure that SF4+ reference models really existed and sought to confirm them by genomic sequences. It appeared that a number of longer models could be confirmed by regular contigs (marked azure) and some, mostly shorter, could not (marked yellow), which left their nature open to investigation. Additionally, some models could be confirmed only by PacBio contigs (accession numbers starting with JSAF02, marked lilac) which also could possibly misrepresent genomic arrangement of tandem repeats, as their sequences were generated by SMRT technology using PacBio's Quiver algorithm to improve the base accuracy [1]. It is not known if this procedure could artificially decrease the divergence of neighboring HOR copies or even create false HORs, so the PacBio contigs were considered to be only a tentative confirmation. Note that artificial contraction of a non-AS HOR domain was demonstrated in clone CH17-41E14 as a result of using SMRT technology [2]. It is possible that an opposite process of artificial expansion could take place as well. Below, we present further details of Table S2 construction. Genomic contigs which contained regions highly identical to the sequences in AS reference models were identified by BLASTing HORs from a reference model to human contigs and HTGS databases. A copy of a HOR to use as a probe was picked up by finding a region in a reference model with regular diagonals and cutting out an appropriate sequence fragment. High-identity (95%-100%) hits were then searched for regular tandem HORs by dot-matrix using REVN program written by V.A. Shepelev [3]. If the sequences with high identity HORs were obtained, only such sequences were listed. If no contigs with regular HORs were found, the sequences with solitary or partial HOR copies were listed and an appropriate note was made. PacBio JSAF02 contigs were listed only if they contained tandem HORs and only if no regular contigs with tandem HORs were found. In some arrays, two or more kinds of variant HORs of different lengths were found intermingled and, therefore, only ragged diagonals were observed on the dot matrix. This was marked as “HOR-like structure”. Similar dot-matrix patterns are often found on the flanks of more regular HOR domains and they contain less perfect copies of the same HOR. As a rule, if good HOR arrays were present nearby, the imperfect HOR domains were not mentioned.

### Supplementary note 1. Analysis of mixed HORs.

In this note we investigated a proposition that unusual mixed AS regions could appear, at least in part, due to misclassification of some atypical monomers which were not adequately described by PERCON matrices. PERCON works best for AS classification only when all the groups of monomers that exist in the sample are represented by appropriate PERCON matrices. If this is not the case, some monomers that do not exactly fit any of the matrices could be classed approximately into classes to which they are more similar. Because this similarity is weaker than to the “own” class, such approximate classification could be strongly affected by random mutations, which may accidentally take a monomer closer to this group or the other. Also note that when a monomer appears to be equally similar to two different PERCON matrices it is not classed and appears as Um (unclassed).

With this in mind we have investigated if the R monomers in the two mixed HORs could in fact be atypical D or J monomers with only partial D- or J-class derived identity. We hypothesized that the monomers of the new family classes could have derived from parental R classes in a step-wise manner. Then, unlike live centromeres, the dead older ones could be formed by “early” generations of the new family classes with incomplete identity. Such monomers could be classed by PERCON into ancestral R classes. In this case, they would have some, but not all of characteristic substitutions which differentiate the parental R classes from the new family derivative classes (a class haplotype). These haplotypes for all new family classes are known [4]. If the D2 class, for instance, differs from parental R2 by 10 mutations (D2 haplotype) and the monomer in question has only 5 of these mutations (incomplete haplotype), PERCON could class it as Um, and if it has only 4 of them it could be classed as R2. However, 4 D2-specific diagnostic positions with high probability would indicate that the monomer belongs to the D2 branch, as only very rarely a typical R2 monomer in a true SF5 array would have 4 such positions in place. Our survey of about 700 R2 monomers in typical SF5 arrays showed that only one R2 monomer in this set had 4 haplotypic positions for D2 and three monomers had 3 such positions (data not shown). In no case were several such monomers found concentrated in a small locality. So, if the R monomers within D/R mixed HOR have some signs of the D identity, and the D monomers have incomplete haplotype, it would indicate an early SF2 array which was partially misclassed by PERCON. And if they are typical R monomers devoid of the D haplotypic positions joined to D monomers with complete haplotype, the mixed HOR would rather be interpreted as a true mix of SF2 and SF5 monomers joined by recombination. Such borderline cases where PERCON indicates an atypical situation should be resolved with full-fledged phylogenetic analysis. If incomplete early haplotypes are established, additional matrices could be introduced to enable PERCON to interpret the partial-identity arrays correctly.

Below we used 4 methods to class the monomers in question:

1. Consensus identity index (CII) calculated as described in [5], which expressed the similarity of two monomers as the percentage of non-consensus bases which were identical between them. It was evaluated separately for type A and type B monomers and, in each group, the monomers in question were compared to consensus monomers of all type A or type B classes (sequences shown in Fig. S1). The differences from the overall type A or type B consensus monomers were used for CII calculation. The “N” positions in consensus monomers were treated as ordinary letters. If the overall type consensus had N in certain position, any nucleotide in a class consensus was counted as a difference, and N in a class consensus was counted as a match. N in a class monomer matched to any nucleotide was counted as a difference. The higher the CII between the two monomers, the more closely related they were. In the tables, the CII figures were shown below the diagonal and above, the percent of identity was shown for comparison. The script used for CII calculation is available at: <https://github.com/enigene/considenti>
2. Rooted phylogenetic trees for sets of monomers, including the monomers in question and consensus monomers of all A- or B-type classes. Routinely, we used minimum evolution (ME) trees constructed in MEGA with default parameters and using the “partial deletion” option of processing deletions and “p-distance” option as substitution model. However, we also checked if neighbor joining (NJ) method used with the same options reproduced a similar tree, which was always the case. The trees were rooted in A- or B-type consensus monomers.
3. Unrooted phylogenetic trees for sets of monomers, including the monomers in question and collections of all human SF1 or SF2 HOR monomers known to us. These sets of HORs and their analysis will be presented and discussed in full elsewhere. All of the live HOR families and a number of dead or pseudo HORs listed in [6] were represented in these collections along with some previously unpublished HORs, the details of which are given in the text below.
4. Nucleotide-by-nucleotide manual analysis of class haplotypic positions, which took into consideration not only the number of differences, but also their direction i.e. the parental, derived, or neutral character of nucleotides in the positions diagnostic of a certain class. This analysis attempted an improved accuracy as the constituent nucleotides of the consensus N positions were taken into consideration as well. In this setting we also evaluated separately the diagnostic positions unique for a certain class i.e. positions not present in any other class of the same type (not counting M1 which chronologically precedes the relationships of the new families and their progenitor R1 and R2 classes).

We used these 4 methods to find out what class the monomers are more likely to belong to (methods 1 and 2), their relationships with the other known monomers of the same class (method 3), and whether they have complete or incomplete haplotype of this class (method 4).

For this analysis, we used the aligned monomers of the mixed HORs extracted from GJ212095.1 (HOR20-2; chr20:28,527,018-28,528,895) and GJ211866.1 (HOR3-2; chr3:90,877,726-90,879,423), and the class consensus monomers from [4] with some modifications as shown in Fig. S1. Since we published this set of AS consensus sequences (see accession numbers in Fig. S1) we have been periodically checking its accuracy using larger sets of classed monomers as they became available. In most cases the difference was insignificant and we continued to use the published sequences. However, the J2 consensus monomer was significantly updated, so the version shown in Fig. S1 should be used instead of AJ130754.1. Also, we found out that the type A consensus monomer AJ131207.1 in GenBank was entered with an error (a deletion of one letter and an insertion of one letter later) which causes an alignment shift. This error is corrected in the sequence shown in Fig.S1.

Our analysis using the methods outlined above revealed that: (i) both HORs had divergent internal ancestral repeats other than D1D2 or J1J2 dimers (tetrameric for HOR20-2 and pentameric for HOR3-2), so in fact only 4 and 5 different monomers formed HOR20-2 and HOR3-2, respectively; (ii) these HORs were not mixtures of typical new SF and SF5 monomers, instead, the new SF monomers had only partial new class haplotypes and most of the R monomers did have some positions which identified them as appropriate new classes; (iii) in each HOR, the ancestral repeat had one monomer which did not fit the same class identity with the others in a definite way, whether these were the accidents of divergence or these were alien monomers introduced into the ancestral arrays by recombination was not clear. Note that one or two monomers per HOR which are classed as Um or R, are typical of SF2 HORs (see SF2 reference models using PERCON track). Thus, overall the mixed HORs should be classed as SF1 (HOR3-2) and SF2 (HOR20-2) and appear to be recent re-amplifications of the divergent material of archaic dead centromeres composed by early new family monomers with partial haplotypes. In SF1, such early monomers with similar partial haplotype are also found in chromosome 6 live HOR (D6Z1) and in a small piece of archaic sequence embedded in chromosome 3 live HOR (D3Z1). The details of this analysis are given below.

### Analysis of monomers from ch20 mixed HOR 11-mer (HOR20-2).

The 11-mer HOR does not have clear internal periodicity other than AB alternation, which is perfectly observed within the HOR if it is taken as R1UmD1D2R1R2R1D2R1R2R1 (monomer classes are shown according to PERCON). As R1(B) monomers are on both ends of the amplification unit, the array has R1R1 sequence on each HOR border, which disturbs the AB periodicity throughout the array. With less clarity, one may see a divergent tetrameric internal structure within the HOR. In the last 8 monomers, the tetramer D2R1R2R1 is duplicated with 86% identity. The first trimer R1UmD1 directly aligns to the tetramer at 83%, but it may be rearranged hence the lower identity. Dimer UmD1 (mon2-3) aligns at 87% to tet1-2, and R1 (mon1) best aligns at 83% to tet4, but the differences to consensus A monomer in the first half of mon1 are shared with tet4 and in the second with tet2 (see below), hence it may be a 4/2 hybrid. So, the HOR structure in terms of the tetramer would be:

| **Mon** | 1 | 2 | 3 | 4 | 5 | 6 | 7 | 8 | 9 | 10 | 11 |
| --- | --- | --- | --- | --- | --- | --- | --- | --- | --- | --- | --- |
| **Type** | B | A | B | A | B | A | B | A | B | A | B |
| **Class** | R1 | Um | D1 | D2 | R1 | R2 | R1 | D2 | R1 | R2 | R1 |
| **Tet** | 4/2 | 1 | 2 | 1 | 2 | 3 | 4 | 1 | 2 | 3 | 4 |

Such HOR may have originated from amplification of a rearranged segment of a divergent tetrameric dead centromere. Mon1 could be created by a deletion in the tetrameric array involving 2 monomers taken out-of-register (half of tet4, complete tet1 and half of tet2). Two more monomers (tet3 and tet4) would have to be deleted after mon3 to obtain the 11-mer HOR unit.

A type monomers.

**Table 1. CII/identity of HOR20-2 type A monomers**

| A-type AS consensus | J1 | D2 | W4 | W5 | R2 | Mon2 Tet1 | Mon4 Tet1 | Mon6 Tet3 | Mon8 Tet1 | Mon10 Tet3 |
| --- | --- | --- | --- | --- | --- | --- | --- | --- | --- | --- |
| J1 AS consensus | - | 85 | 86 | 82 | 89 | 80 | 83 | 82 | 78 | 78 |
| D2 AS consensus | 0 | - | 89 | 83 | 95 | 90 | 94 | 89 | 88 | 85 |
| W4 AS consensus | 7 | 10 | - | 88 | 95 | 85 | 88 | 87 | 84 | 84 |
| W5 AS consensus | 21 | 0 | 26 | - | 88 | 79 | 82 | 80 | 78 | 75 |
| R2 AS consensus | 0 | 18 | 18 | 0 | - | 89 | 94 | 91 | 88 | 86 |
| HOR20-2 Mon2 Tet1 Um | 5 | **41** | 7 | 0 | 10 | - | 89 | 85 | 82 | 81 |
| HOR20-2 Mon4 Tet1 D2 | 0 | **45** | 9 | 0 | 15 | **39** | - | 87 | 90 | 85 |
| HOR20-2 Mon6 Tet3 R2 | 0 | **23** | 8 | 0 | 0 | 17 | 7 | - | 84 | 84 |
| HOR20-2 Mon8 Tet1 D2 | 0 | **31** | 6 | 5 | 9 | **24** | **47** | 11 | - | 81 |
| HOR20-2 Mon10 Tet3 R2 | 5 | **29** | 17 | 0 | 8 | 23 | 27 | **29** | 21 | - |

- CII calculated on type A consensus monomer is shown below the diagonal. Identity between the two monomers compared is shown above the diagonal.

At first glance, the HOR is more SF5 than SF2, but CII shows that all the A monomers have by far the highest scores with consensus D2, regardless of whether they are Um, R2, or D2 monomers by PERCON. Also tet1 monomers and tet3 monomers have the highest scores with their own kind. Of D2 haplotype substitutions (see below), all but one are present in the A monomers of this HOR. So, these monomers are clearly D2, and overall almost complete D2 haplotype seems to be present. Other tests are as follows.


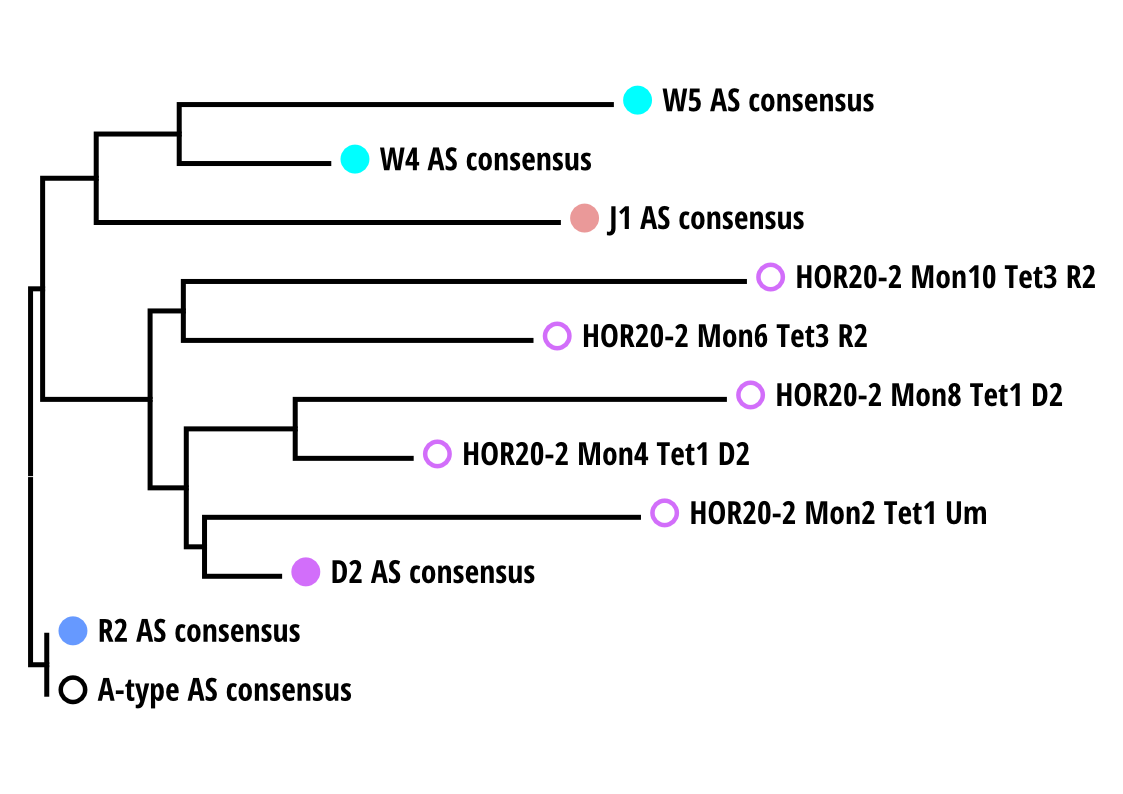


On the tree of consensus A monomers, all HOR monomers group with D2 and all other A classes are grouped in a separate branch.


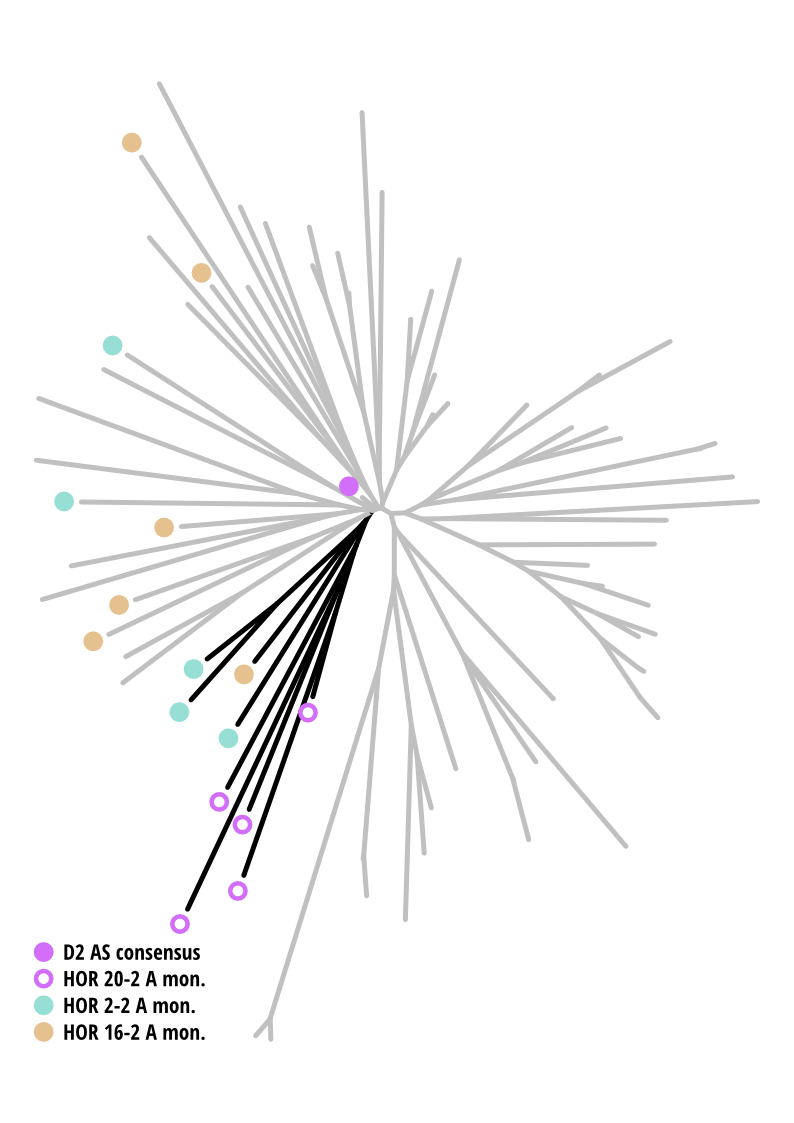


On the tree of all D2 HOR monomers, the A monomers from HOR20-2 all go in one branch (marked black) and group with one monomer from HOR16-2 and three monomers from HOR2-2. However, the other monomers from these HORs are present in other branches as well. HOR16-2 is a 12-mer present on the p-side of the live SF1 HOR on chromosome 16 (chr16:34,257,712-34,272,290) and in GJ212046. Our sample copy of this HOR matches chr16:34,263,280-34,265,318. HOR2-2 is an SF2 10-mer—it is not present among the SF2 reference models included in hg38 assembly. Genomic AS sequences related to this HOR are located on both sides of the live SF2 HOR on chromosome 2 (on the p-side at chr2:92,087,958-92,117,670 and on q-side at chr2:94,166,713-94,189,216). Our sample copy of this HOR best matches chr2:92,097,896-92,099,603. Both HOR16-2 and HOR2-2 are either dead SF2 HORs or pseudocentromeres which have amplified dead SF2 material. They possess a number of archaic features which are not present in any of the live SF2 HORs and also lack a number of features that are present in most or all live SF2 HORs (data will be presented in full elsewhere). One of the advanced features characteristic of the live HORs is the presence of D1 monomers with a 4 bp deletion in positions 55-58. In live SF2 HORs, this subclass of D1 makes up about a half of the D1 monomers and such monomers are present in all the live HORs. The non-alive HORs 2-2, 16-2, and 20-2 lack this D1 subclass, but another non-alive SF2 HOR18-2 (D18Z2) has this subclass. Thus, HOR20-2 seems to fit in the archaic group as a third member. Notably, the branch which has the HOR20-2 A monomers does not have a long stem and is not significantly removed from the center of the tree, so the possible group of archaic monomers is not that much different from the bulk of D2.

**Table 2. Summary of D2 haplotype analysis**

| Mon | Tet | Class | Dif from A | D2 shared | D2 unique |
| --- | --- | --- | --- | --- | --- |
| 2 | 1 | Um | 19 | 7/10 | 5/8 |
| 4 | 1 | D2 | 12 | 6/10 | 4/7 |
| 6 | 3 | R2 | 16 | 4/10 | 3/7 |
| 8 | 1 | D2 | 22 | 6/10 | 4/7 |
| 10 | 3 | R2 | 25 | 7/10 | 5/8 |

Manual analysis of D2 haplotype positions is summarized above and the full data are shown in Table 3 below. Table 2 shows a total number of differences with consensus monomer A for each HOR20-2 type A monomer, as well as two indices based on these differences. The “D2 shared” index shows a number of differences shared with D2 (out of 10 positions in D2 haplotype). This index shows a generous estimate of affinity to D2 class. The “D2 unique” index shows a number of shared unique positions in D2 haplotype (which occur only in D2, but not in any other class of type A) related to a number of haplotype positions which were informative in such comparison. This index shows the most conservative estimate of D2 affinity. Even tet3 monomers with the least number of D2 diagnostic positions in mon6 share at least 3 unique D2 positions informative in this comparison. Thus, it is considerably removed from progenitor A type towards D2 having about a half-complete haplotype. For comparison, the next closest runner for tet3 is W4, with a unique score of 1/9.

**Table 3. Complete D2 haplotype analysis of HOR20-2 A monomers**

| **Consensus/ Monomer name** | **D2 haplotype positions** | | | | | | | | | |
| --- | --- | --- | --- | --- | --- | --- | --- | --- | --- | --- |
|  | **1** | **7** | **15** | **43** | **60** | **95** | **109** | **111** | **118** | **170** |
| **A cons.** | A | C | G/C | G | A | C | C | T | C | A |
| **D2 cons.** | T | G | C | A | C | G/A | T | C | A | G/A |
| **Mon2 Tet1** | T | G | C | A | A | C | T | C | C | A |
| **Mon4 Tet1** | T | G | C | A | A | C | T | G | C | A |
| **Mon6 Tet3** | A | T | A | G | C | C | T | C | C | A |
| **Mon8 Tet1** | T | G | C | A | A | C | T | G | C | A |
| **Mon10 Tet3** | A | C | C | A | C | G | T | C | C | A |
| **HOR2-2 Mon2** | A | G | C | A | G | G | T | C | A | A |
| **HOR2-2 Mon4** | A | G | C | A | G | G | T | C | A | A |
| **HOR2-2 Mon6** | T | G | C | A | C | C | T | C | A | A |
| **HOR2-2 Mon8** | T | G | C | A | G | G | T | C | A | A |
| **HOR2-2 Mon10** | T | G | C | A | C | G | T | C | A | A |
| **HOR16-2 Mon2** | A | G | C | A | C | G | T | C | A | A |
| **HOR16-2 Mon4** | T | G | C | - | C | G | T | C | A | A |
| **HOR16-2 Mon6** | T | G | C | A | C | G | T | C | A | A |
| **HOR16-2 Mon8** | T | G | C | A | C | G | T | C | A | G |
| **HOR16-2 Mon10** | T | G | C | A | A | G | T | C | A | A |
| **HOR16-2 Mon12** | T | G | C | A | C | G | T | C | A | A |

- The haplotype includes all positions different or partially different between consensus monomers A and D2. Consensus monomer A was derived from all class consensus monomers including M1 with 50% threshold. The letters in N positions of monomer A (where more than one letter is present) are shown only if that letter comes from a consensus monomer other than D2 or M1 which is not included in this comparison.
- D2 shared positions are marked lilac, ancestral positions are marked grey, and non-informative positions are white. Nucleotides unique for D2 are underlined.
- The names of the monomers of HOR2-2 and HOR16-2 which group on the same branch of D2 tree with HOR20-2 monomers are underlined.
- The same rules are used in Tables 7, 10, and 14 below.

Overall, of the D2 substitutions only one (A in 118) is not present in any of the A monomers. Among SF2 HORs it is a unique feature. Only solitary monomers with A in position 118 are found in other HORs (data not shown). All other D2 substitutions are present in at least 2 monomers. As a control, 14 of 18 J1 substitutions, 7 out of 10 W4 substitutions, and 17 out of 21 W5 substitutions are completely absent in the set. So, overall the HOR has almost complete set of D2 diagnostic positions. In the same way, HORs 2-2 and 16-2 have complete D2 haplotypes with no positions dominated by ancestral configuration, as is typical of all other human SF2 HORs (data not shown). Note that some deterioration of the haplotype in individual monomers would be expected due to accumulation of random mutations during the divergence process, which made copies of the tetramer only ~86% identical. Thus, the monomers which have had a complete haplotype when the tetramer array was alive and homogeneous would lose some of their diagnostic positions upon divergence. However, only a fraction of these would revert to ancestral configuration. A high number of ancestral positions (grey color in the table) would indicate that, even before the divergence of tetramers, their haplotype was not complete.

Conclusion on HOR20-2 A monomers: All A monomers should be regarded as D2 and the D2 haplotype in individual HOR20-2 monomers is about half-complete, but overall all but one D2-diagnostic substitutions are present.

B type monomers

**Table 4. CII/identity of HOR20-2 type B monomers**

| B-type AS consensus | J2 | D1 | W1 | W2 | W3 | R1 | Mon1 Tet4/2 | Mon3 Tet2 | Mon5 Tet2 | Mon7 Tet4 | Mon9 Tet2 | Mon11 Tet4 |
| --- | --- | --- | --- | --- | --- | --- | --- | --- | --- | --- | --- | --- |
| J2 AS consensus | - | 85 | 86 | 81 | 85 | 89 | 81 | 81 | 78 | 84 | 81 | 80 |
| D1 AS consensus | 6 | - | 88 | 83 | 86 | 91 | 85 | 90 | 86 | 85 | 87 | 83 |
| W1 AS consensus | 17 | 20 | - | 81 | 88 | 89 | 82 | 84 | 80 | 83 | 83 | 81 |
| W2 AS consensus | 5 | 6 | 0 | - | 80 | 87 | 81 | 80 | 80 | 81 | 80 | 77 |
| W3 AS consensus | 16 | 12 | 33 | 0 | - | 88 | 81 | 83 | 81 | 82 | 82 | 79 |
| R1 AS consensus | 16 | 0 | 8 | 7 | 8 | - | 87 | 88 | 86 | 89 | 88 | 85 |
| HOR20-2 Mon1 Tet4/2 R1 | 0 | **23** | 5 | 14 | 10 | 14 | - | 84 | 82 | 82 | 82 | 80 |
| HOR20-2 Mon3 Tet2 D1 | 5 | **41** | 16 | 10 | 20 | 14 | 33 | - | 85 | 82 | 87 | 80 |
| HOR20-2 Mon5 Tet2 R1 | 0 | **26** | 10 | 13 | 23 | 12 | 30 | **39** | - | 83 | 88 | 79 |
| HOR20-2 Mon7 Tet4 R1 | 22 | 6 | 11 | 10 | 5 | 23 | 15 | 10 | 18 | - | 83 | 82 |
| HOR20-2 Mon9 Tet2 R1 | 5 | **29** | 15 | 9 | 15 | 21 | 27 | **42** | **55** | 20 | - | 81 |
| HOR20-2 Mon11 Tet4 R1 | 18 | 15 | 14 | 4 | 4 | 12 | 17 | 13 | 16 | **27** | 21 | - |

The CII shows that tet4/2 and tet2 monomers are definitely classed as D1, while tet4 are not. Of the latter, mon11 has relatively high D1 score and for mon7 it is very low. Thus it is possible that tet4 partially shared D2 haplotype, but in mon7 it was accidentally ruined by mutations.


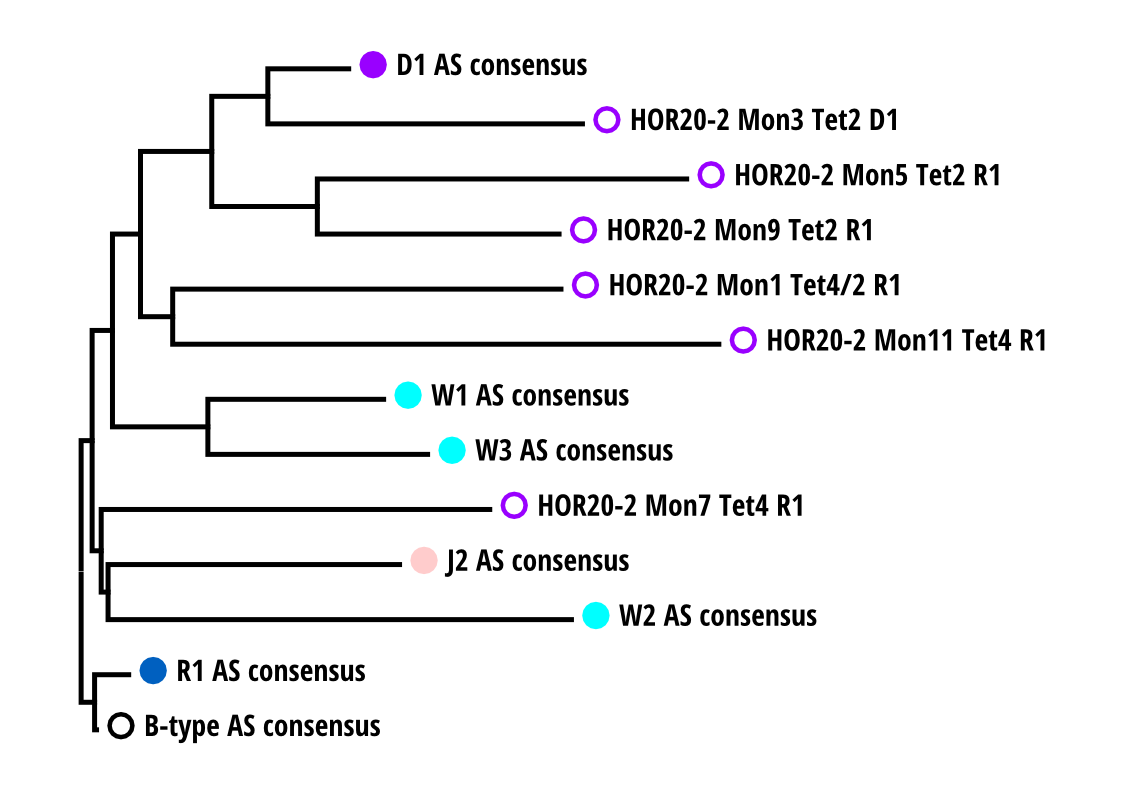


On the tree of consensus B monomers there are two main branches with the HOR monomers. In one branch, mon7 (tet4) groups with J2 and W2. In the other, one tet4 monomer is grouped with D1 and all the other HOR monomers. Therefore the tree may support the notion that mon7 is a wayward tet4 which lost its partial D1 identity.


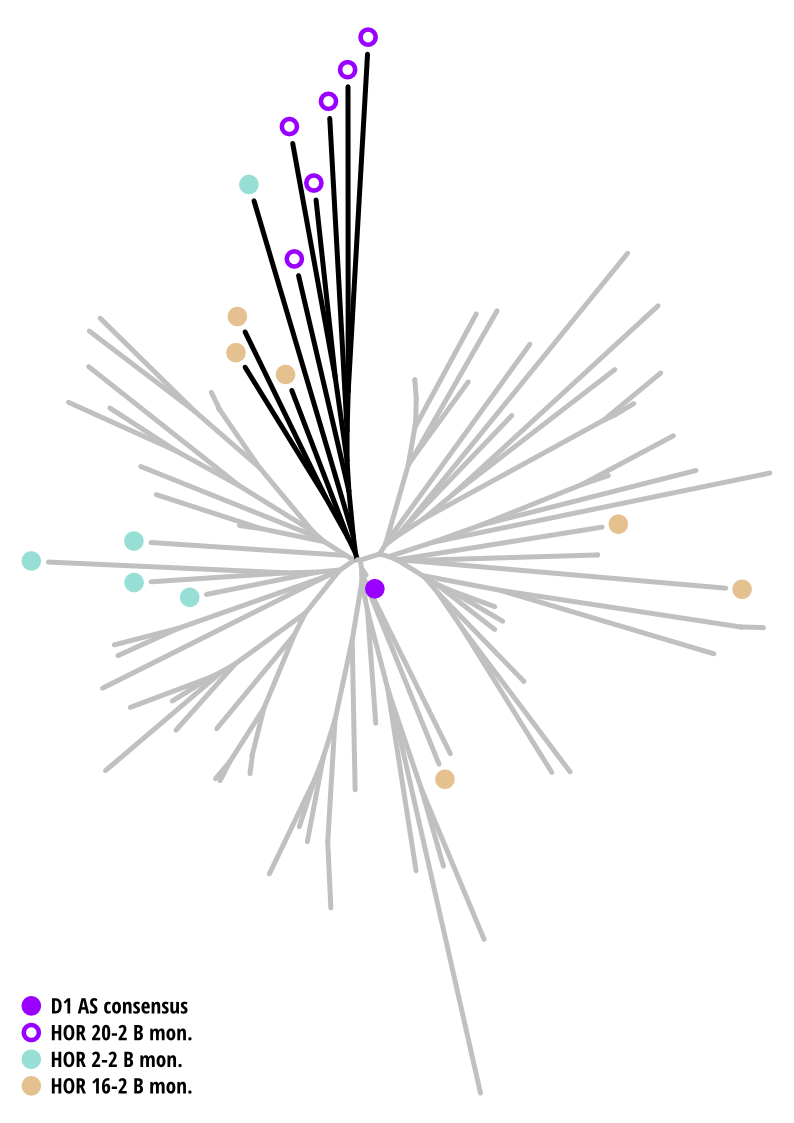


On the tree of all HOR D1 monomers, the B monomers of HOR20-2 all go in one branch and group with one monomer from HOR2-2 and 3 monomers from HOR16-2. Similar features were observed for the A monomers (see the comments above). As discussed above, HOR20-2 lacks D1 monomers with 4 bp deletion in positions 55-58, a feature shared with HOR2-2 and HOR16-2.

**Table 5. Summary of D1 haplotype analysis**

| Mon | Tet | Class | Dif from B | D1 shared | D1 unique |
| --- | --- | --- | --- | --- | --- |
| 1 | 4/2 | R1 | 22 | 6/14 | 2/10 |
| 3 | 2 | D1 | 21 | 9/14 | 4/10 |
| 5 | 2 | R1 | 25 | 7/14 | 3/10 |
| 7 | 4 | R1 | 19 | 2/14 | 0/11 |
| 9 | 2 | R1 | 22 | 7/14 | 3/11 |
| 11 | 4 | R1 | 26 | 4/14 | 1/10 |

Substitutions in tet4.

Non-consensus tet4 positions shared with all type B classes are shown in Table 6. Tet4 have deletions reminiscent of J2—both mon7 and mon11 have one in position 68, and mon11 has one more in position 50 which can be moved to 52 to match J2 at the cost of one mismatch, however as optimal alignment goes it is a different deletion. Thus, tet4 may be an alien early J2 introduced by recombination (mon7 rather looks like it) or an early D1 with incomplete haplotype, which lost most of D1 diagnostic positions by chance (mon11 is compatible with it). Note that mon7 and mon11 are both tet4 and most likely are just diverged copies of the same progenitor tet4.

**Table 6. Shared substitutions in tet4.**

| **Consensus/ Monomer name** | **Position** | | | | | | | | | | | **Shared substitutions** |
| --- | --- | --- | --- | --- | --- | --- | --- | --- | --- | --- | --- | --- |
|  | **24** | **25** | **34** | **68** | **92** | **106** | **119** | **131** | **150** | **152** | **170** |  |
| **Tet4** | A | G | A | - | G | G | C | C | A/T | C | G | - |
| **R1 consensus** |  | G/T |  |  |  | G |  |  | T |  |  | 3/8 |
| **D1 consensus** | A | G | A |  |  |  |  |  |  |  |  | 3/14 |
| **W1 consensus** |  |  | A |  |  |  |  | C |  |  |  | 2/17 |
| **W2 consensus** |  |  |  |  | G |  |  |  | T |  | G | 3/21 |
| **W3 consensus** |  |  | A |  |  |  | C |  |  | C/T |  | 3/19 |
| **J2 consensus** |  |  |  | - |  | G |  | C | A |  | G | 5/18 |

- Matching substitution may be present in only one of tet4 monomers.

Substitutions in mon1.

Mon1 best aligns at 83% to tet4, but some substitutions are shared with tet4 (positions 4, 23, 35, 134, 144) and some with tet2 (positions 68, 95, 105, 106, 130, 131, 140), hence it is of the mixed identity and may be a hybrid with positions 1-35 coming from tet4 and positions 68-171 coming from tet2 with the breakpoint somewhere in 36-67 region (see alignment of haplotypic positions in these monomers in Table 7; marked by **).

Absence of 4 bp deletion.

HOR20-2 lacks D1 monomers with 4 bp deletion in positions 55-58, a feature shared with HOR2-2 and HOR16-2, but not with another non-alive HOR18-2 (D18Z2). The latter seemingly represents a more recent generation of SF2 HORs, the one shared by all live SF2 centromeres.

In the D1 haplotype, 4 out of 14 positions (61, 68, 116 and 150) are not present in any of the B monomers. All others are present in 1 (position 136) or more monomers. As a control, 11 of 18 J2 and 13 out of 21 W2 substitutions are completely absent. So, even if tet4 is excluded as alien, the D1 haplotype in the set is incomplete and perhaps reflects some early stage of D1 evolution. In other SF2 HORs, these positions are well conserved, A in 68 and C in 116 are shared in some HORs while C in 61 and T in 150 are almost unique to HOR20-2. Note that even archaic HORs 2-2 and 16-2 have complete D1 haplotypes with no positions dominated by ancestral configuration, as is typical of all other human SF2 HORs (data not shown).

**Table 7. Complete D1 haplotype analysis of HOR20-2 B monomers**

| **Consensus/ Monomer name** | **D1 haplotype positions** | | | | | | | | | | | | | |
| --- | --- | --- | --- | --- | --- | --- | --- | --- | --- | --- | --- | --- | --- | --- |
|  | **23** | **24** | **25** | **34** | **35** | **57** | **61** | **68** | **106** | **116** | **131** | **136** | **140** | **150** |
| **B cons.** | C | C | T/A/G | C | C | T | C | C/A/-* | T/C/G | C | C/A/T | T | G | G/T/C |
| **D1 cons.** | T | A | G | A | T | T/- | T | T | T/C | G | A | C | C | G |
| **Mon1 Tet4/2** | C | A | G | C | C | T | C | A | T | C | A | T | C | T |
| **Mon3 Tet2** | T | A | C | A | T | T | C | A | T | C | A | C | C | T |
| **Mon5 Tet2** | T | A | G | C | T | T | C | A | T | C | T | G | C | T |
| **Mon7 Tet4** | C | T | G | C | C | T | C | - | G | C | C | T | G | T |
| **Mon9 Tet2** | T | A | G | C | T | T | C | A | T | C | C | T | C | T |
| **Mon11 Tet4** | C | A | G | A | A | T | C | - | G | C | C | T | G | A |
| **Tet4**** | C | A | G | C | C | T | C | - | G | C | C | T | G | T |
| **Tet4/2**** | C | A | G | C | C | T | C | A | T | C | A | T | C | T |
| **Tet2**** | T | A | G | C | T | T | C | A | T | C | A | T | C | T |
| **HOR2-2 Mon1** | C | A | G | A | T | T | T | T | T | G | A | C | C | G |
| **HOR2-2 Mon3** | A | A | G | A | T | T | C | T | T | G | G | T | C | G |
| **HOR2-2 Mon5** | C | A | G | A | T | T | T | T | T | G | A | C | C | G |
| **HOR2-2 Mon7** | T | A | G | A | T | T | T | T | T | G | A | C | C | C |
| **HOR2-2 Mon9** | T | A | G | A | T | T | T | C | T | G | A | C | C | G |
| **HOR16-2 Mon1** | T | T | G | A | T | T | T | -* | T | C | A | C | T | G |
| **HOR16-2 Mon3** | T | A | G | A | T | C | T | T | T | G | A | C | C | G |
| **HOR16-2 Mon5** | C | A | G | A | T | T | T | A | T | G | A | C | C | G |
| **HOR16-2 Mon7** | T | T | G | A | T | T | T | -* | T | G | A | G | C | G |
| **HOR16-2 Mon9** | T | A | G | A | T | T | T | T | T | G | A | C | C | G |
| **HOR16-2 Mon11** | T | A | G | A | T | T | T | A | A | G | A | C | C | G |

*Unlike single nucleotide deletion in position 68 in tet4 monomers of HOR20-2, which is shared with J2, other deletions that overlap this position seem to be different and independent events. In HOR16-2, the deletion is a part of a 3 bp deletion in positions 68-70, which is followed by a single nucleotide deletion in position 74. This combination of deletions is found only in two D1 monomers of HOR16-2. This deletion is likely distinct and unrelated to 3 bp W1 deletion in positions 66-68, which contributes to position 68 in type B consensus monomer. So we considered all 3 deletions as separate mutations.

**This section shows that monomer tet4/2 with mixed identity may be a hybrid with positions 1-35 coming from tet4 and positions 68-171 coming from tet2 with the breakpoint somewhere in the 36-67 region.

Conclusion on HOR20-2 B monomers: tet2 monomers should be regarded as D1 with a partial haplotype. Tet4 may be an early J2 or some atypical B type monomer introduced by recombination.

Overall conclusion on HOR20-2. The HOR is a product of sequential amplification of early SF2 tetramer in which one monomer could have been an atypical B monomer (tet4 i.e. mon7 and mon11) possibly introduced by recombination into an early SF2 array while three other monomers are recognizable SF2. Both D2 monomers in the tetramer have incomplete haplotype, but between themselves they have all but one D2 mutations. The only definite D1 monomer has incomplete D1 haplotype, and the second B monomer may be an alien early J2 or progenitor R1. Note that D2 is a minimalistic haplotype of 8 substitutions, which possibly does not have early and mature stages. On the trees, both A and B monomers of HOR20-2 group with HOR2-2 and HOR16-2 monomers, both non-alive SF2 HORs. Additionally, HORs 20-2, 2-2, and 16-2 all lack D1 with 4 bp deletion, which seems to be a late development in SF2 evolution. So, HOR20-2 represents an early generation of SF2. It seems likely that it is a pseudocentromere which amplified the divergent repeats of an ancient dead centromere (tetramers). Such divergent tetramers (86% identity) could have formed only if a dead early SF2 centromere had underwent hypermutability, as we previously reported for freshly dead centromeres [7].

### Analysis of monomers from chromosome 3 mixed HOR 10-mer (HOR3-2).

The HOR is a 10-mer composed of two 84% identical pentamers.

Pentamer is R2J2J1R1(Um)J1 = ABABA.

10mer is R2J2J1R1J1R2J2J1UmJ1 = ABABAABABA.

| **Mon** | 1 | 2 | 3 | 4 | 5 | 6 | 7 | 8 | 9 | 10 |
| --- | --- | --- | --- | --- | --- | --- | --- | --- | --- | --- |
| **Type** | A | B | A | B | A | A | B | A | B | A |
| **Class** | R2 | J2 | J1 | R1 | J1 | R2 | J2 | J1 | Um | J1 |
| **Pen** | 1 | 2 | 3 | 4 | 5 | 1 | 2 | 3 | 4 | 5 |

Presumably the AB alternation is intact within the HOR, but on the pentamer and 10-mer borders the AA sequence reiterates due to amplification register. The pentameric array might have been an early SF1 centromere with partial J haplotypes before becoming a dead centromere after it was replaced by the current live SF1 centromere of chromosome 3 (D3Z1 which is also termed HOR3-1 here). The pentamers in the dead HOR3-2 have diverged considerably, and recently two such diverged pentamers were amplified, yielding a pseudocentromere with homogeneous 10-mer HOR.

A type monomers

**Table 8. CII/identity of HOR3-2 type A monomers**

| A-type AS consensus | J1 | D2 | W4 | W5 | R2 | Mon1 Pen1 | Mon3 Pen3 | Mon5 Pen5 | Mon6 Pen1 | Mon8 Pen3 | Mon10 Pen5 |
| --- | --- | --- | --- | --- | --- | --- | --- | --- | --- | --- | --- |
| J1 AS consensus | - | 85 | 86 | 82 | 89 | 76 | 85 | 77 | 80 | 83 | 75 |
| D2 AS consensus | 0 | - | 89 | 83 | 95 | 78 | 83 | 74 | 82 | 80 | 75 |
| W4 AS consensus | 7 | 10 | - | 88 | 95 | 83 | 84 | 77 | 85 | 79 | 75 |
| W5 AS consensus | 21 | 0 | 26 | - | 88 | 77 | 79 | 72 | 82 | 75 | 70 |
| R2 AS consensus | 0 | 18 | 18 | 0 | - | 82 | 88 | 78 | 87 | 83 | 80 |
| HOR3-2 Mon1 Pen1 R2 | 8 | 0 | **20** | 15 | 0 | - | 78 | 71 | 82 | 73 | 70 |
| HOR3-2 Mon3 Pen3 J1 | **21** | 0 | 7 | 5 | 0 | 24 | - | 77 | 80 | 88 | 80 |
| HOR3-2 Mon5 Pen5 J1 | **21** | 0 | 12 | 10 | 0 | 20 | 28 | - | 74 | 76 | 74 |
| HOR3-2 Mon6 Pen1 R2 | 10 | 0 | 19 | **23** | 0 | **42** | 14 | 23 | - | 76 | 74 |
| HOR3-2 Mon8 Pen3 J1 | **30** | 0 | 5 | 12 | 0 | 17 | **57** | 33 | 16 | - | 79 |
| HOR3-2 Mon10 Pen5 J1 | **15** | 0 | 4 | 4 | 0 | 18 | 36 | **36** | 18 | 38 | - |


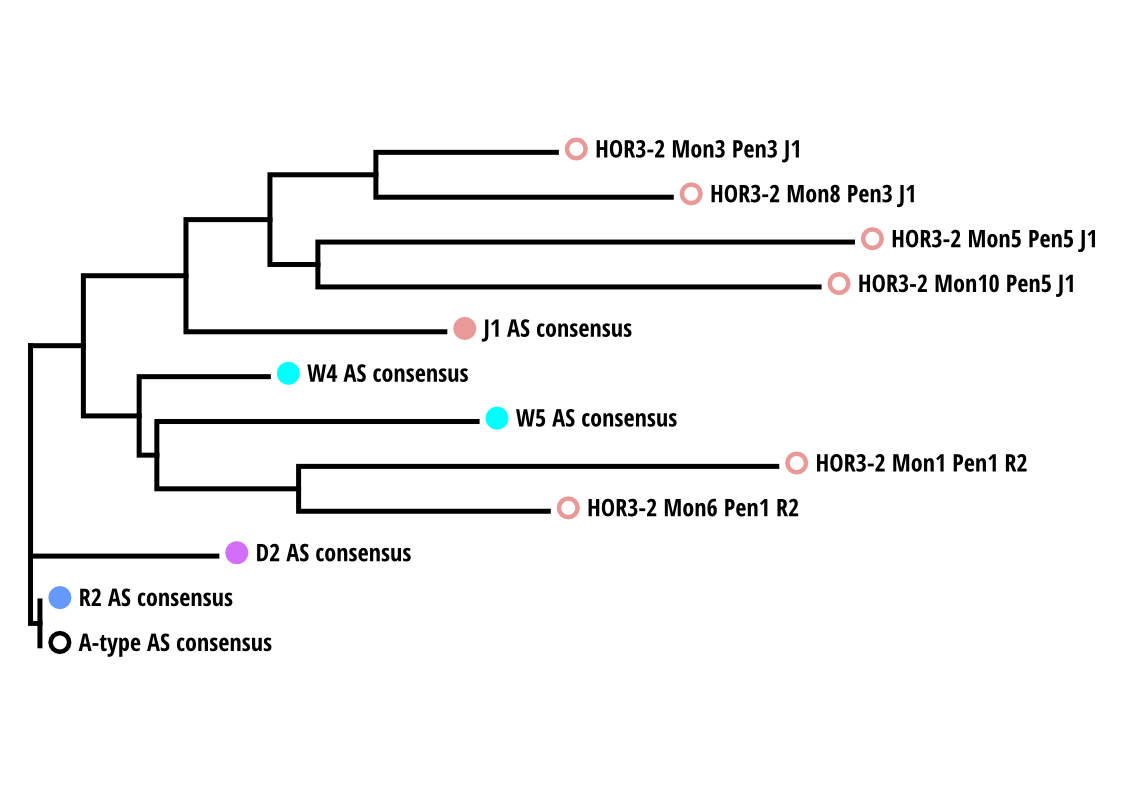


On the tree of type A and on CII, Pen1 (PERCON R2) monomers group with W4 and W5 while Pen3 and 5 (PERCON J1) group with J1, making the origin of Pen1 questionable. Analysis of relationships between the A classes (not shown) reveals that W4 and W5 are especially close (they share 6 substitutions) and J1 and W4/5 definitely have a common ancestor, but their origin cannot be derived in a linear fashion. It seems there was a recombination event or some complex rearrangement (data not shown), which means that Pen1, which is similar to W4/5, may be a type A monomer close to the common ancestor of J1W4/5 group. Alternatively, it may be an early SF3 monomer introduced into SF1 array by recombination.


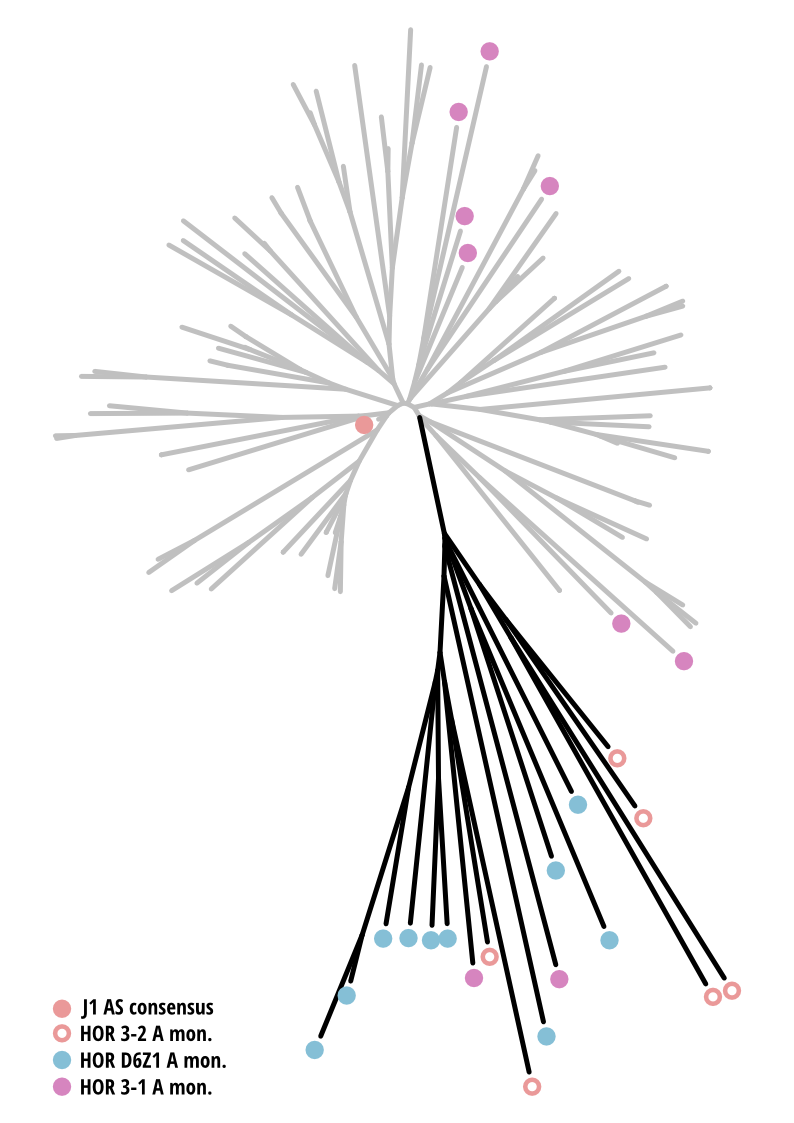


On the tree of all HOR J1 monomers, the HOR3-2 monomers form a separate branch with a long stem that they share with the monomers from D6Z1, the live HOR from chromosome 6 (AB005791) [8]. Two more monomers in this branch come from D3Z1, the live HOR from chromosome 3 (Z12006) [9]. Only one of them is classed Um by PERCON—the other one, as well as all monomers from chromosome 6, are classed as J1. A number of other monomers from D3Z1 are present in other branches as well.

**Table 9. Summary of J1 haplotype analysis**

| Mon | Pen | Class | Dif from A | J1 shared | J1 unique |
| --- | --- | --- | --- | --- | --- |
| 1 | 1 | R2 | 31 | 4/18 | 1/14 |
| 3 | 3 | J1 | 20 | 6/18 | 3/14 |
| 5 | 5 | J1 | 38 | 8/18 | 5/14 |
| 6 | 1 | R2 | 22 | 3/18 | 1/15 |
| 8 | 3 | J1 | 29 | 9/18 | 4/16 |
| 10 | 5 | J1 | 35 | 6/18 | 3/15 |

Analysis of haplotype positions shows only partial J1 identity in pen3 and pen5 and hardly any in pen1. Of 18 J1 haplotype positions, 5 unique positions have ancestral configuration in all HOR monomers. So, even if pen1 is excluded as alien, the J1 haplotype is only partial. However, the J1-unique T in position 7 is present in all monomers, including pen1.

**Table 10. Complete J1 haplotype analysis of HOR3-2 A monomers**

| **Consensus/ Monomer name** | **J1 haplotype positions** | | | | | | | | | | | | | | | | | |
| --- | --- | --- | --- | --- | --- | --- | --- | --- | --- | --- | --- | --- | --- | --- | --- | --- | --- | --- |
|  | **4** | **15** | **20** | **21** | **34** | **36** | **42** | **60** | **61** | **79** | **94** | **110** | **127** | **140** | **152** | **153** | **164** | **171** |
| **A cons.** | C | C/G | G | G | C | T | G | A | C | G | T | A | G | G | T | T | T | G |
| **J1 cons.** | T | G | C | A | T | A | A | G | T | T | A/G | G | T | C | T/A | G | G | A |
| **Mon1 Pen1** | T | T | A | G | C | T | A | A | C | G | A | C | G | G | T | T | T | G |
| **Mon3 Pen3** | T | T | A | G | C | T | G | A | T | T | A | C | T | G | T | T | T | G |
| **Mon5 Pen5** | T | T | A | C | T | T | A | A | C | T | G | A | T | G | T | G | T | G |
| **Mon6 Pen1** | T | T | A | G | C | T | A | A | C | G | T | A | G | G | T | T | T | G |
| **Mon8 Pen3** | T | T | A | G | C | T | A | G | T | T | A | G | T | G | A | T | T | G |
| **Mon10 Pen5** | T | T | A | G | C | T | A | A | C | T | A | A | T | G | T | T | T | G |
| **D6Z1 Mon1** | T | T | A | G | T | T | A | A | T | T | G | G | T | G | C | T | T | G |
| **D6Z1 Mon3** | T | T | A | T | C | T | A | C | T | T | G | G | A | G | T | T | T | G |
| **D6Z1 Mon4** | T | T | A | G | C | C | A | A | C | G | A | A | G | T | T | T | T | G |
| **D6Z1 Mon6** | T | T | A | G | C | T | A | A | T | T | A | G | T | G | T | T | C | A |
| **D6Z1 Mon7** | C | T | T | G | C | T | G | A | C | G | G | G | T | G | T | A | C | G |
| **D6Z1 Mon8** | T | T | A | G | C | T | A | C | C | G | C | A | G | G | T | G | T | G |
| **D6Z1 Mon10** | T | C | A | G | C | T | A | C | C | G | A | A | C | G | T | G | T | G |
| **D6Z1 Mon12** | T | T | G | G | C | T | A | C | C | C | A | A | G | G | T | T | T | G |
| **D6Z1 Mon14** | T | T | A | G | C | T | A | C | C | G | A | A | G | G | T | T | T | G |
| **D6Z1 Mon17** | T | T | A | G | C | T | A | C | C | G | A | A | G | G | T | T | T | G |
| **D3Z1 Mon1** | T | G | C | A | C | A | A | G | T | T | G | A | T | C | T | G | G | A |
| **D3Z1 Mon3** | T | G | C | A | C | A | G | G | T | T | A | A | T | C | T | G | G | A |
| **D3Z1 Mon5** | T | A | C | T | C | A | A | G | T | T | G | A | G | C | T | G | G | C |
| **D3Z1 Mon7** | T | G | C | T | C | A | A | G | T | T | A | G | T | C | T | G | G | A |
| **D3Z1 Mon9** | T | T | T | G | C | T | T | C | T | T | A | A | C | T | A | T | C | G |
| **D3Z1 Mon10** | T | T | A | G | C | T | A | C | C | G | G | A | C | A | T | T | T | G |
| **D3Z1 Mon12** | T | G | C | A | C | A | A | G | T | T | G | G | T | C | T | C | T | G |
| **D3Z1 Mon14** | T | G | C | - | C | A | A | G | T | T | A | G | T | C | T | G | G | A |
| **D3Z1 Mon16** | T | G | C | A | T | A | A | G | T | T | A | G | T | C | T | G | T | G |

- The names of D3Z1 monomers that group with HOR3-2 on the J1 tree are underlined.
- Note that, in positions 15 and 20, all the A monomers have unique nucleotides not present in any of the A classes.
- Note that typical “mature” J1 monomers (mon1, 3, 5, 7, 12, 14 and 16) from D3Z1 have 1-3 ancestral positions (grey) while early J1 monomers from HOR3-2 and D6Z1 have 7-13.

**Table 11. Shared substitutions in pen1.**

| **Consensus/ Monomer name** | **Position** | | | | | | | | | | **Shared substitutions** |
| --- | --- | --- | --- | --- | --- | --- | --- | --- | --- | --- | --- |
|  | **4** | **37** | **38** | **42** | **94** | **95** | **106** | **131** | **134** | **143** |  |
| **Pen1** | T | G | C | A | A | A | T | A | C | G | - |
| **R2 consensus** |  |  |  |  |  |  |  |  |  |  | 0/1 |
| **D2 consensus** |  |  |  |  |  | A/G |  |  |  |  | 1/10 |
| **W4 consensus** |  | G |  |  | A |  | T | A | C |  | 5/10 |
| **W5 consensus** |  |  | C/T | A |  |  | T | A | C | G | 6/21 |
| **J1 consensus** | T |  |  | A | A/G |  |  |  |  |  | 3/18 |

- Matching substitution may be present in only one of pen1 monomers.
- Each new family class shares 1 unique substitution with pen1 and W5 shares two.

Of 18 J1 haplotype positions, 7 are completely absent in HOR3-2 and 5 of them are dominated by parental configuration (21, 36, 140, 164, 171), so J1 haplotype is incomplete. Most of these absences are shared with D6Z1 and only sporadically with other HORs. As a control, in typical “mature” J1 monomers of D3Z1 (all, but mon8 and 9), all J1 haplotype positions are present in at least half of the monomers. Overall, the “mature” part of D3Z1 has a complete J1 haplotype, as is typical of all other live human SF1 HORs (data not shown).

Conclusion on HOR3-2 A monomers: Pen3 and pen5 are early J1 monomers with incomplete haplotype. Pen1 may be an unusual monomer close to J1W4/5 ancestor (i.e. very early J1) or a very early W4/5 introduced by recombination into an early SF1 array and then amplified.

B type monomers


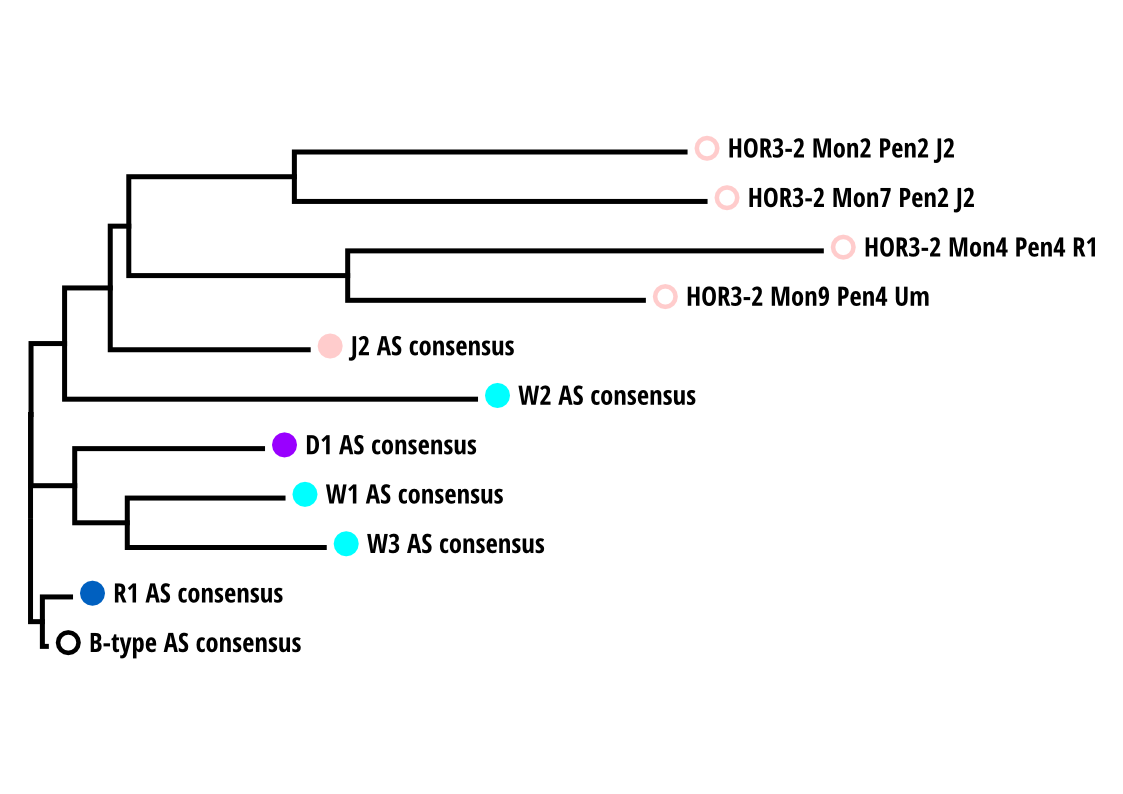


**Table 12. CII/identity of HOR3-2 type B monomers**

| B-type AS consensus | J2 | D1 | W1 | W2 | W3 | R1 | Mon2 Pen2 | Mon4 Pen4 | Mon7 Pen2 | Mon9 Pen4 |
| --- | --- | --- | --- | --- | --- | --- | --- | --- | --- | --- |
| J2 AS consensus | - | 85 | 86 | 81 | 85 | 89 | 84 | 81 | 82 | 82 |
| D1 AS consensus | 6 | - | 88 | 83 | 86 | 91 | 80 | 77 | 79 | 80 |
| W1 AS consensus | 17 | 20 | - | 81 | 88 | 89 | 80 | 78 | 78 | 80 |
| W2 AS consensus | 5 | 6 | 0 | - | 80 | 87 | 78 | 73 | 77 | 78 |
| W3 AS consensus | 16 | 12 | 33 | 0 | - | 88 | 79 | 75 | 77 | 79 |
| R1 AS consensus | 16 | 0 | 8 | 7 | 8 | - | 84 | 81 | 83 | 84 |
| HOR3-2 Mon2 Pen2 J2 | **35** | 5 | 13 | 12 | 9 | 17 | - | 77 | 84 | 78 |
| HOR3-2 Mon4 Pen4 R1 | **31** | 4 | 16 | 4 | 8 | 15 | 33 | - | 76 | 84 |
| HOR3-2 Mon7 Pen2 J2 | **33** | 5 | 13 | 12 | 4 | 16 | **52** | 32 | - | 76 |
| HOR3-2 Mon9 Pen4 Um | **27** | 5 | 9 | 12 | 9 | 12 | 25 | **50** | 25 | - |

According to CII, all HOR3-2 B monomers are clear J2 and, on the tree of consensus B monomers, they all sit on one branch with J2. All other class consensus monomers are on other branches.


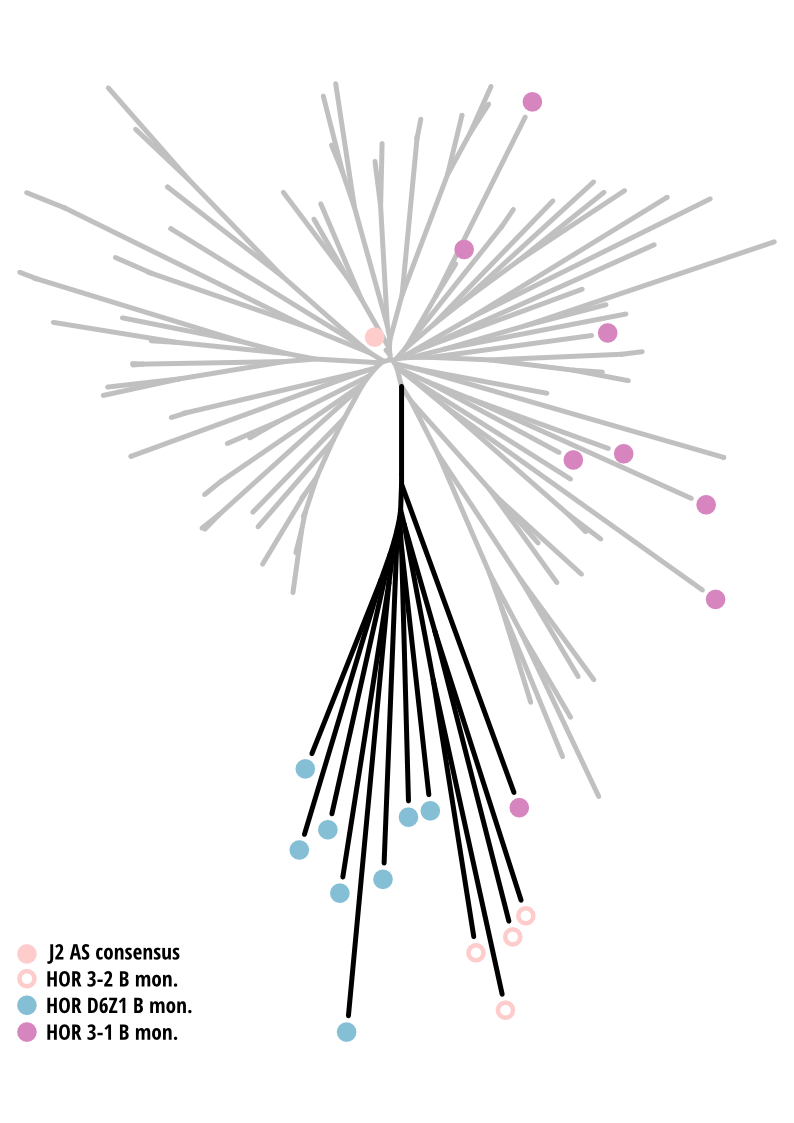


On the tree of all J2 monomers, the ones from HOR3-2 sit on a very divergent branch with D6Z1 J2 monomers, which is the only such protruding branch in J2 tree. One monomer from D3Z1 is also in this branch. All monomers in the branch except HOR3-2 pen4 monomers are classed as J2 by PERCON. This tree is very similar to the tree of all J1 HOR monomers (see comments above).

**Table 13. Summary of J2 haplotype analysis**

| Mon | Pen | Class | Dif from B | J2 shared | J2 unique |
| --- | --- | --- | --- | --- | --- |
| 2 | 2 | J2 | 28 | 9/18 | 4/12 |
| 4 | 4 | R1 | 33 | 9/18 | 4/12 |
| 7 | 2 | J2 | 30 | 9/18 | 4/12 |
| 9 | 4 | Um | 27 | 7/18 | 4/13 |

**Table 14. Complete J2 haplotype analysis of HOR3-2 B monomers**

| **Consensus/ Monomer name** | **J2 haplotype positions** | | | | | | | | | | | | | | | | | |
| --- | --- | --- | --- | --- | --- | --- | --- | --- | --- | --- | --- | --- | --- | --- | --- | --- | --- | --- |
|  | **2** | **25** | **52** | **54** | **61** | **66** | **67** | **68** | **80** | **89** | **102** | **106** | **109** | **131** | **139** | **142** | **150** | **170** |
| **B cons.** | A | T/G /A | A | A | C | A | A | A/-*C/T | C | A | A | G/T C | C | C/A T | T | T | G/T /C | A |
| **J2 cons.** | G | T | - | T | T | T | G | - | A | T | T | G | T | C | A | C/T | A | G |
| **Mon2 Pen2** | A | T | - | T | T | A | A | - | A | A | A | G | C | C | C | T | T | A |
| **Mon4 Pen4** | A | T | - | T | T | A | A | - | A | A | A | G | C | C | C | T | T | A |
| **Mon7 Pen2** | A | T | - | T | T | A | A | - | A | A | A | G | C | C | C | T | T | A |
| **Mon9 Pen4** | A | T | - | T | C | A | C | - | A | A | A | G | C | A | T | T | T | A |
| **D6Z1 Mon2** | A | T | - | T | T | C | T | - | A | A | A | G | C | C | T | C | T | A |
| **D6Z1 Mon5** | A | T | - | T | T | C | C | - | A | A | A | G | C | C | T | T | T | A |
| **D6Z1 Mon9** | A | T | - | T | T | A | C | - | A | A | A | G | C | A | T | T | T | A |
| **D6Z1 Mon11** | A | G | - | T | T | A | C | - | A | A | A | G | A | C | T | T | T | A |
| **D6Z1 Mon13** | A | T | - | T | T | A | T | - | A | A | T | G | C | C | G | T | T | A |
| **D6Z1 Mon15** | A | G | - | T | T | C | T | - | A | A | A | T | C | A | T | T | T | A |
| **D6Z1 Mon16** | A | T | - | T | T | A | T | - | A | A | A | G | C | C | G | T | T | A |
| **D6Z1 Mon18** | A | T | - | T | T | A | C | - | A | T | A | G | C | A | T | T | T | A |
| **D3Z1 Mon2** | G | T | - | T | T | T | G | - | A | T | - | G | T | C | A | C | A | G |
| **D3Z1 Mon4** | T | T | - | T | C | T | G | - | A | T | A | A | T | C | A | C | A | G |
| **D3Z1 Mon6** | G | T | - | T | T | T | T | T | A | T | - | G | T | C | A | C | A | G |
| **D3Z1 Mon8** | G | T | - | T | T | A | C | - | A | A | A | G | C | C | G | A | T | A |
| **D3Z1 Mon11** | A | T | - | T | T | T | G | - | A | T | G | G | T | G | A | C | A | G |
| **D3Z1 Mon13** | A | T | - | T | T | T | G | - | A | T | T | G | T | T | A | A | A | G |
| **D3Z1 Mon15** | G | T | - | T | T | T | G | - | A | T | T | G | T | C | A | - | - | G |
| **D3Z1 Mon17** | A | T | - | T | T | T | G | - | A | T | T | G | T | C | A | C | A | G |

*Deletion in position 68 of J2 overlaps a 3 bp deletion in positions 66-68 of W1. These two deletions were counted as two independent and different mutation events. Note that positions of these deletions may vary a little bit depending on alignment.

- The name of D3Z1 monomer which groups with HOR3-2 on J2 tree is underlined.
- Note that typical “mature” J2 monomers (mon2, 4, 6, 11, 13, 15 and 17) from D3Z1 have 1-2 ancestral positions (grey) while early J2 monomers from HOR3-2 and D6Z1 have 7-10.

The J2 monomers are remarkably homogeneous and all have the same incomplete J2 haplotype. Nine J2 positions (2, 66, 67, 89, 102, 109, 139, 150 and 170) out of 18 are completely absent, so the J2 haplotype is about half complete. Most of these absences are shared with D6Z1. Also, J2 has 11 unique substitutions, of which 4 are present in the sequence (counting deletion in position 68 as unique and independent of deletion 66-68 in W3). As a control, in typical “mature” J2 monomers of D3Z1 (all, but mon8), all J2 haplotypic positions are present in at least half of the informative monomers. Overall, the “mature” part of D3Z1 has a complete J2 haplotype, as is typical of all other live human SF1 HORs (data not shown).

Conclusion on HOR3-2 B monomers: All J2 monomers are clearly early J2 with half complete haplotype.

Overall conclusion on HOR3-2: The HOR is made mostly by clear, but early, J monomers with only partial J haplotypes, along with one A monomer that belongs to an even earlier type, perhaps closer to the ancestor of J1W4/5 group or to an alien early W4/5 monomer introduced by recombination. Similar early SF1 monomers form the live chromosome 6 HOR and a three-monomer piece of archaic SF1 sequence embedded in chromosome 3 live HOR. Note that divergent inner pentamers in HOR3-2 (84% identity) could have formed only if a dead early SF1 centromere had undergone hypermutability, as we previously reported for freshly dead centromeres [7].

Concluding remarks

It is possible that the first generations of SF1 and SF2 HORs were formed by monomers with widely varying degrees of class identity, having both featureless ancestral monomers and more mature new family monomers with half-complete haplotypes. Further generations of SF2 and SF1 HORs have acquired and fixed yet more mutations, which resulted in the formation of haplotype and subclasses structure present in the majority of contemporary live HORs. This confirms the idea of a step-wise formation of contemporary new family haplotypes. In most cases, featureless ancestral monomers did not survive further evolution and were not propagated in contemporary live HORs.

[1] C.S.Chin, D.H.Alexander, P.Marks, A.A.Klammer, J.Drake, C.Heiner, A.Clum, A.Copeland, J.Huddleston, E.E.Eichler, S.W.Turner, J.Korlach, Nonhybrid, finished microbial genome assemblies from long-read SMRT sequencing data, Nat. Methods 10 (6), 2013 Jun, 563-569, http://dx.doi.org/10.1038/nmeth.2474, (Epub 2013 May 5. PubMed PMID: 23644548).

[2] J.Huddleston, S.Ranade, M.Malig, F.Antonacci, M.Chaisson, L.Hon, P.H.Sudmant, T.A.Graves, C.Alkan, M.Y.Dennis, R.K.Wilson, S.W.Turner, J.Korlach, E.E.Eichler, Reconstructing complex regions of genomes using long-read sequencing technology, Genome Res.24 (4) 2014 Apr, 688-696.

[3] V.A.Shepelev, N.V.Yanishevsky, Multidimensional dot-matrices, Comput. Appl. Biosci. 10 (6), 1994 Dec, 605-611, (Supplement 2).

[4] L.Y.Romanova, G.V.Deriagin, T.D.Mashkova, I.G.Tumeneva, A.R.Mushegian, L.L.Kisselev, I.A.Alexandrov. Evidence for selection in evolution of alpha satellite DNA: the central role of CENP-B/pJ alpha binding region. J Mol Biol. 261(3), 1996 Aug 23; 334-340, (PubMed PMID: 8780776).

[5] J.S.Waye, H.F.Willard. Chromosome-specific alpha satellite DNA: nucleotide sequence analysis of the 2.0 kilobasepair repeat from the human X chromosome. Nucleic Acids Res. 13(8), 1985 Apr 25, 2731-2743, (PubMed PMID: 2987865; PubMed Central PMCID: PMC341190).

[6] I.Alexandrov, A.Kazakov, I.Tumeneva, V.Shepelev, Y.Yurov. Alpha-satellite DNA of primates: old and new families. Chromosoma. 110(4), 2001 Aug, 253-266, (PubMed PMID: 11534817).

[7] V.A.Shepelev, A.A.Alexandrov, Y.B.Yurov, I.A.Alexandrov. The evolutionary origin of man can be traced in the layers of defunct ancestral alpha satellites flanking the active centromeres of human chromosomes. PLoS Genet. 5 (9), 2009 Sep; e1000641, http://dx.doi.org/10.1371/journal.pgen.1000641, (Epub 2009 Sep 11. PubMed PMID: 19749981; PubMed Central PMCID: PMC2729386).

[8] K.Sugimoto, K.Furukawa, K.Kusumi, M.Himeno, The distribution of binding sites for centromere protein B (CENP-B) is partly conserved among diverged higher order repeating units of human chromosome 6-specific alphoid DNA. Chromosome Res 5, 1997, 395-405

[9] I.A.Alexandrov, T.D.Mashkova, L.Y.Romanova, Y.B.Yurov, L.L.Kisselev. Segment

substitutions in alpha satellite DNA. Unusual structure of human chromosome 3-specific alpha satellite repeat unit. J Mol Biol. 231(2), 1993 May 20, 516-520, (PubMed PMID: 8510162).
